# Supplementary material for: A cross–sectional study on the prevalence and associated risk factors for workplace violence against Chinese nurses
Source: BMJ Open. 2017 Jun 24;7(6):e013105. doi: 10.1136/bmjopen-2016-013105 (PMC5623406; doi:10.1136/bmjopen-2016-013105)
Supplement: Research Checklist [file bmjopen-2016-013105supp001.doc]

***Checklist of cross-sectional studies***

|  | Page number, line number |
| --- | --- |
| **Title** | 1, 4-6 |
| **Abstract** | 2, 4-58 |
| **Strengths and limitations of this study** | 3, 4-27 |
| **Introduction** |  |
| Background | 4, 4-57; 5, 4-14 |
| Objectives | 5, 16-24 |
| **Methods** |  |
| Study design | 5, 37-52 |
| Setting | 5, 52-57; 6, 4-19 |
| Participants | 6, 21-27 |
| Questionnaire | 6, 41-57; 7, 4-29 |
| Data measurement | 7, 36-57; 8, 4 |
| Study size | 6, 28-32 |
| Quantitative variables | 7, 36-42 |
| Ethical considerations | 8, 11-19 |
| **Results** |  |
| Participants | 8, 26-34 |
| Descriptive data | 8, 36-58; 9, 3-48 |
| Outcome data | 9, 52-57 |
| Main results | 10; 11; 12;14 |
| Other analyses | 10, 8-15; 12 |
| Discussion |  |
| Key results | 13,32-57; 15, 4-56 |
| Interpretation | 16, 3-32; 17, 9-57 |
| Discuss the external validity of the study results | 17, 9-27 |
| **Conclusions** | 18, 51-57; 19, 4-7 |
| Other information |  |
| Acknowledgements | 18, 14-19 |
| Contributors | 18, 24-27 |
| Funding | 18, 34-36 |
| Competing interests | 18, 34 |
| Ethical approval | 18, 47-52 |
| **References** | 19; 20; 21; 22; 23; 24 |
