# Supplementary material for: A cross–sectional study on the prevalence and associated risk factors for workplace violence against Chinese nurses
Source: BMJ Open. 2017 Jun 24;7(6):e013105. doi: 10.1136/bmjopen-2016-013105 (PMC5623406; doi:10.1136/bmjopen-2016-013105)
Supplement: Author's manuscript [file bmjopen-2016-013105.draft_revisions.pdf]

BMJ Open

# A cross-sectional study on the prevalence and associated risk factors of workplace violence against Chinese nurses

| | |
|---|---|
| Journal: | *BMJ Open* |
| Manuscript ID | bmjopen-2016-013105 |
| Article Type: | Research |
| Date Submitted by the Author: | 20-Jun-2016 |
| Complete List of Authors: | Shi, Lei; School of Public Health, Harbin Medical University, Department of Health Management<br>Zhang, Danyang; The First Affiliated Hospital of Harbin Medical University, Operating Section<br>Zhou, Chenyu; School of Public Health, Harbin Medical University, Department of Health Management<br>Yang, Libin; School of Public Health, Harbin Medical University, Department of Medical Education<br>Sun, Tao; School of Public Health, Harbin Medical University, Department of Health Management<br>Hao, Tianjun; The Second Affiliated Hospital of Harbin Medical University, Department of Scientific Research<br>Peng, Xiangwen ; The First Affiliated Hospital of Harbin Medical University, Harbin, Operating Section<br>Gao, Lei; School of Public Health, Harbin Medical University, Department of Health Management<br>Liu, Wenhui; School of Public Health, Harbin Medical University, Department of Medical Education<br>Mu, Yi; Beijing Children's Hospital, Capital Medical University, Department of Medical Matters<br>Han, Yuzhen; The Fourth Affiliated Hospital of Harbin Medical University, Department of Medical Matters<br>Fan, Lihua; School of Public Health, Harbin Medical University, Department of Health Management |
| <b>Primary Subject Heading</b>: | Health services research |
| Secondary Subject Heading: | Health policy, Medical management, Nursing |
| Keywords: | workplace violence (WPV), nurses, general public hospitals, risk factors |
| | |

SCHOLARONE™
Manuscripts

# A cross-sectional study on the prevalence and associated risk factors of workplace violence against Chinese nurses

Lei Shi, [1] Danyang Zhang,[2] Chenyu Zhou,[1] Libin Yang,[3] Tao Sun,[1] Tianjun Hao,[4] Xiangwen Peng,[2] Lei Gao,[1] Wenhui Liu,[3] Yi Mu,[5] Yuzhen Han, [6] Lihua Fan[1]

**Correspondence to:** Lihua Fan, School of Public Health, Harbin Medical University, 157 Baojian Road, Nangang District, Harbin 150081, China; E-mail: lihuafan@126.com;

[1]Department of Health Management, School of Public Health, Harbin Medical University, Harbin, China

[2] Operating Section, The First Affiliated Hospital of Harbin Medical University, Harbin, China

[3]Department of Medical Education, School of Public Health, Harbin Medical University, Harbin, China

[4]Department of Scientific Research, The Second Affiliated Hospital of Harbin Medical University, Harbin, China

[5]Department of Medical Matters, Beijing Children's Hospital, Capital Medical University, Beijing, China

[6] Department of Medical Matters, The Fourth Affiliated Hospital of Harbin Medical University, Harbin, China

**Keywords:** workplace violence (WPV); nurses; general public hospitals; risk factors

For peer review only

**Abstract**

**Objectives:** The purpose of the present study was to explore the epidemic characteristics of workplace violence that Chinese nurses of tertiary hospitals and county-level hospitals have encountered in the past 12 months, to identify and to analyze the risk factors, thus providing the basis for future preventive strategies.

**Design:** A cross-sectional sampling survey was conducted to collect the data.

**Participants:** A total of 9142 nurses from 44 tertiary hospitals and 6828 nurses from 90 county-level hospitals located in 16 provinces of China.

**Results:** The total prevalence of workplace violence was 65.8% (10502/15970), of which the verbal violence accounted for 64.9%, physical violence and sexual harassment accounted for 11.8% and 3.9% respectively. Frequent workplace violence mainly occurred in the emergency department and the pediatrics department. Respondents claimed that patients' relatives were the main perpetrators in tertiary hospitals (83.1%) and county-level hospitals (85.2%). Logistic regression analysis showed that participants' age, department, years of experience and the nurses direct contact with patients were common risk factors in different levels of hospitals.

**Conclusions**: The high incidence of workplace violence is the main feature. Workplace violence among nurses is an important issue that cannot be overlooked. A series of laws, policies, and interventions should be implemented to cope with workplace violence.

**Strengths and limitations of this study**

First, we collected data over the past 12 months about whether nurses had experienced violence, so the recall bias may exist. Second, we conducted a study of tertiary hospitals and county-level hospitals, but did not study specialized hospitals. Third, further researches on the psychological harm of WPV are

For peer review only

necessary to be carried out.

**BACKGROUND**

Workplace violence (WPV) that towards health professionals in the health service systems has been recognized as a global public health issue and caused broad attention in the worldwide.[1-4] Previous studies have indicated that health professionals are at the highest risk for experiencing violence in all work environments.[2] Besides, the incidence rate of WPV among healthcare workers in various countries are different, for instance, it was 67.2% in Australia, 46.7% in Brazil, 61% in South Africa, and 37% in Portugal.[5]

On this basis, nurses are more likely to experience WPV than other staff in hospitals. During the past 12 months, incidence rate of physical violence in Ethiopia,[6] South Korea[7] and Jordan[8] ranged from 18.22% to 52.8%, verbal abuse from 63.8% to 89.58% and sexual harassment from 13.02% to 19.7%. The main concentration of WPV events is in the emergency ward and psychiatric department of hospitals. [9-10] Research on the nurses of these departments in the US,[11] Switzerland[12] and Taiwan[2] has demonstrated a higher prevalence of WPV.

In China, WPV in hospitals has been gradually increasing over the past years. From 2000 to 2012, the physician-patient conflicts presented a growing trend, the number of doctors who were killed by patients went up to 14, and more people were injured. [13] According to the report of Chinese Hospital Association, the proportion of hospitals experiencing violence has risen from 90% (2008) to 96% (2012); and the prevalence of sexual harassment also increased year by year.[14]

Although the violent incidents in hospitals directly harm the healthcare workers and hospitals, the ultimate victims are still patients.[15] WPV affects the normal order and reputation of the hospitals, and threatens the personal safety of healthcare workers and patients.[16] Moreover, violent incidents also have

For peer review only

a negative impact on the psychological aspect of the healthcare workers, which may lead to the phenomenon that healthcare workers do not hope their children to be engaged in medical work.

The purpose of the present study was to explore the distribution characteristics of WPV that Chinese nurses of tertiary hospitals and county-level hospitals have encountered in the last 12 months, to further identify and to analyze the risk factors, thus providing the basis for future preventive strategies.

**METHODS**

A cross-sectional survey was designed based on the geographical location and the level of economic development in the East (Beijing, Tianjin, Hebei, Shandong, Guangdong, Heilongjiang, Liaoning), Middle (Shanxi, Henan, Anhui, Hunan) and West (Ningxia, Shannxi, Gansu, Sichuan, Chongqing) regions of China. According to a certain proportion, 120 nurses were extracted from department of Internal medicine, Surgery, 80 nurses were extracted from department of Emergency, Neurology, Obstetrics and Gynecology, Pediatrics, and 40 nurses were selected from department of Stomatology, Ophthalmology, Ear, Nose and Throat in each of tertiary hospital. A total of 10560 questionnaires were distributed in 44 tertiary hospitals. By the same method, the nurses were chosen by the 60, 40 and 20 from those departments at the county-level hospital. A total of 10800 questionnaires were distributed in 90 county-level hospitals.

The inclusion criteria for participants are as follows: the nurses engaged in clinical work should be with at least one year professional experience in hospitals and participated voluntarily. The permission was acquired from all 134 hospitals throughout the study.

The survey was conducted from December 2014 to January 2016. We obtained permission from

For peer review only

the managers and human resources departments of the hospitals. The researchers used a stratified sampling method to collect the data. Each participant had 2 days to accomplish the self-administered questionnaire. These data were kept confidential and used only for academic research. A total of 21360 questionnaires were handed out, and 15970 valid questionnaires were recovered (effective response rate=74.77%).

**QUESTIONNAIRE**

We developed the questionnaire based on the following three aspects. First, we used the revised SOVES-G-R about the Survey of Violence Experienced by Staff (SOVES) from Hahn and colleagues to develop the questionnaire.[17] Then, we referenced a literature about a comparative study of workplace violence in psychiatric hospitals and general hospitals in China.[18] In addition, we also referenced the questionnaires of the ILO, ICN, WHO, and PSI joint program for measuring WPV. [19] We obtained permission to use these questionnaires. Futhermore, we invited 18 healthcare-related experts to assess the accuracy, comprehensiveness and sensitivity about all of the items in the questionnaire. The questionnaire was pre-tested among a total of 367 nurses from 3 public tertiary hospitals and 2 public county-level hospitals. Then, the questionnaire was further revised. After repeated measurements, the reliability and validity of questionnaires were 0.86 and 0.84, respectively.

**DATA ANALYSIS**

EpiData version 3.1 was used to establish a database. After finishing all the surveys, data was checked by trained personnel to ensure data accuracy. IBM SPSS19.0 and Excel were used for statistical analysis of the relevant quantitative data. Univariate analysis and chi-square tests ($\chi^2$) were used to

For peer review only

analyze the relationship between the demographic characteristics of respondents and the prevalence of WPV. We test whether the nurses suffering from hospital WPV as the Dependent Variable, and the variables with statistical and clinical practice significance as the Covariates in logistic regression analysis. Binary logistic regression analyses on the risk factors of WPV were conducted among the nurses in public tertiary hospitals and county-level hospitals. Odds ratios (OR) and 95% confidence intervals (CI) were calculated; α=0.05 was the level of a test; and $P$<0.05 was considered statistically significant.

**ETHICAL CONSIDERATIONS**

We obtained consent from each hospital in the entire research processes. All of the participants were given the informed consent before the survey, and the participants' personal information was kept confidential.

**RESULTS**

**Demographic characteristics of the participants**

A total of 15970 participants accord with our inclusion criteria. Of the 15970 respondents, most of them had finished their undergraduate studies, and the age group was concentrated on less than 30 years old, but there was a large difference in the proportion of gender (Table 1).

**Table 1** Demographic characteristics of the participants(N=15970)

| Characteristics | Tertiary hospitals (n=9142) | | County-level hospitals (n=6828) | | Total (n=15970) | |
|---|---|---|---|---|---|---|
| | n | % | n | % | n | % |
| Gender | | | | | | |
| Male | 234 | 2.6 | 143 | 2.1 | 377 | 2.4 |
| Female | 8908 | 97.4 | 6685 | 97.9 | 15593 | 97.6 |
| Age group (years) | | | | | | |
| ≤30 | 5131 | 56.1 | 4033 | 59.1 | 9164 | 57.4 |
| 31-50 | 3785 | 41.4 | 2611 | 38.2 | 6396 | 40.0 |

For peer review only

| | | | | | | |
|---|---|---|---|---|---|---|
| ≥51 | 226 | 2.5 | 184 | 2.7 | 410 | 2.6 |
| Education status | | | | | | |
| Below undergraduate | 3129 | 34.2 | 4161 | 60.9 | 7290 | 45.6 |
| Undergraduate | 5910 | 64.6 | 2663 | 39.0 | 8573 | 53.7 |
| Master or above | 103 | 1.1 | 4 | 0.1 | 107 | 0.7 |
| Marital status | | | | | | |
| Married | 5678 | 62.1 | 4293 | 62.9 | 9971 | 62.4 |
| Unmarried | 3342 | 36.6 | 2415 | 35.4 | 5757 | 36.1 |
| Divorced or Widowed | 122 | 1.3 | 120 | 1.7 | 242 | 1.5 |
| Professional title | | | | | | |
| Junior | 6249 | 68.4 | 4845 | 71.0 | 11094 | 69.5 |
| Intermediate | 2242 | 24.5 | 1587 | 23.2 | 3829 | 24.0 |
| Senior | 651 | 7.1 | 396 | 5.8 | 1047 | 6.5 |
| Employment form | | | | | | |
| Regular staff | 5077 | 55.5 | 3339 | 48.9 | 8416 | 52.7 |
| Temporary employee | 4065 | 44.5 | 3489 | 51.1 | 7554 | 47.3 |
| Average monthly income (RMB) | | | | | | |
| ≤3000 | 3747 | 41.0 | 4916 | 72.0 | 8663 | 54.3 |
| 3000-5000 | 4495 | 49.2 | 1867 | 27.3 | 6362 | 39.8 |
| 5000-10000 | 872 | 9.5 | 42 | 0.6 | 914 | 5.7 |
| >10000 | 28 | 0.3 | 3 | 0.1 | 31 | 0.2 |
| Department | | | | | | |
| Emergency | 514 | 5.6 | 522 | 7.6 | 1036 | 6.5 |
| Internal medicine | 2850 | 31.2 | 1962 | 28.7 | 4812 | 30.1 |
| Surgery | 1903 | 20.8 | 1386 | 20.3 | 3289 | 20.6 |
| Gynaecology and Obstetrics | 426 | 4.7 | 610 | 8.9 | 1036 | 6.5 |
| Paediatrics | 516 | 5.6 | 627 | 9.2 | 1143 | 7.2 |
| Others | 2933 | 32.1 | 1721 | 25.3 | 4654 | 29.1 |
| Years of experience | | | | | | |
| 1-4 | 3511 | 38.4 | 3124 | 45.7 | 6635 | 41.5 |
| 5-10 | 2803 | 30.7 | 1597 | 23.4 | 4400 | 27.6 |
| 11-20 | 1534 | 16.8 | 1099 | 16.1 | 2633 | 16.5 |
| ≥21 | 1294 | 14.1 | 1008 | 14.8 | 2302 | 14.4 |
| Working time | | | | | | |
| 0-2 h | 164 | 1.8 | 52 | 0.8 | 216 | 1.3 |
| 2-4 h | 252 | 2.8 | 120 | 1.8 | 372 | 2.3 |
| 4-6 h | 264 | 2.9 | 113 | 1.6 | 377 | 2.4 |
| 6-8 h | 3713 | 40.6 | 3087 | 45.2 | 6800 | 42.6 |
| >8 h | 4749 | 51.9 | 3456 | 50.6 | 8205 | 51.4 |
| Direct contact with patients | | | | | | |
| 0-2 h | 180 | 2.0 | 106 | 1.6 | 286 | 1.8 |
| 2-4 h | 237 | 2.6 | 251 | 3.7 | 488 | 3.0 |
| 4-6 h | 747 | 8.2 | 575 | 8.4 | 1322 | 8.3 |
| 6-8 h | 7978 | 87.2 | 5896 | 86.3 | 13874 | 86.9 |

For peer review only

**Frequency of WPV exposure in the last 12 months**

In the last 12 months, 10502 nurses have experienced WPV in the hospitals. The total prevalence of WPV was 65.8%, of which the verbal violence accounted for 64.9% (10369/15970), physical violence accounted for 11.8% (1892/15970), and sexual harassment accounted for 3.9% (630/15970). The prevalence of WPV in different kinds of hospitals is shown in Table 2.

**Table 2** Prevalence (%) of WPV exposure

| Type | Tertiary Hospitals | | | | | | County-level hospitals | | | | | |
|---|---|---|---|---|---|---|---|---|---|---|---|---|
| | Verbal violence | | Physical violence | | Sexual harassment | | Verbal violence | | Physical violence | | Sexual harassment | |
| | n | % | n | % | n | % | n | % | n | % | n | % |
| | 5875 | 64.3 | 1047 | 11.5 | 402 | 4.4 | 4494 | 65.8 | 845 | 12.4 | 228 | 3.3 |

**Prevalence of WPV in different demographic variables**

The nurses who have frequent contact with patients were more likely to experience WPV in the tertiary hospitals and county-level hospitals (Table 3). In the tertiary hospitals, there was a significant difference in the incidence rate of WPV among different professional titles ($\chi^2$=6.6, $p$<0.05). On the contrary, there was no significant difference in the county-level hospitals ($\chi^2$=1.5, $p$>0.05).

**Table 3** Characteristics and frequency distributions for WPV

| Characteristics | Tertiary hospitals (n=9142) | | | | County-level hospitals (n=6828) | | | |
|---|---|---|---|---|---|---|---|---|
| | n | % | $\chi^2$ | $p$ | n | % | $\chi^2$ | $p$ |
| Gender | | | | | | | | |
| Male | 143 | 61.1 | 1.8 | 0.181 | 85 | 59.4 | 3.2 | 0.072 |
| Female | 5820 | 65.3 | | | 4454 | 66.6 | | |
| Age | | | | | | | | |
| ≤30 | 3283 | 64.0 | 15.4 | <0.001 | 2679 | 66.4 | 15.6 | <0.001 |
| 31-50 | 2548 | 67.3 | | | 1762 | 67.5 | | |
| ≥51 | 132 | 58.4 | | | 98 | 53.3 | | |
| Education | | | | | | | | |
| Below undergraduate | 1958 | 62.6 | 15.7 | <0.001 | 2707 | 65.1 | 9.7 | 0.008 |
| Undergraduate | 3941 | 66.7 | | | 1829 | 68.7 | | |
| Master or above | 64 | 62.1 | | | 3 | 75.0 | | |
| Marital status | | | | | | | | |
| Married | 3813 | 67.2 | 27.8 | <0.001 | 2884 | 67.2 | 3.3 | 0.192 |
| Unmarried | 2065 | 61.8 | | | 1581 | 65.5 | | |
| Divorced or Widowed | 85 | 69.7 | | | 74 | 61.7 | | |

For peer review only

| | | | | | | | | |
|---|---|---|---|---|---|---|---|---|
| Professional title | | | | | | | | |
| Junior | 4022 | 64.4 | 6.6 | 0.037 | 3217 | 66.4 | 1.5 | 0.482 |
| Intermediate | 1607 | 71.7 | | | 1068 | 67.3 | | |
| Senior | 434 | 66.7 | | | 254 | 64.1 | | |
| Employment form | | | | | | | | |
| Regular staff | 3290 | 64.8 | 0.9 | 0.341 | 2213 | 66.3 | 0.1 | 0.733 |
| Temporary employee | 2673 | 65.8 | | | 2326 | 66.7 | | |
| Average monthly income (RMB) | | | | | | | | |
| ≤3000 | 2418 | 64.5 | 1.4 | 0.710 | 3275 | 66.6 | 0.3 | 0.955 |
| 3000-5000 | 2953 | 65.7 | | | 1233 | 66.0 | | |
| 5000-10000 | 574 | 65.8 | | | 29 | 69.0 | | |
| >10000 | 18 | 64.3 | | | 2 | 66.7 | | |
| Department | | | | | | | | |
| Emergency | 418 | 81.3 | 101.2 | <0.001 | 429 | 82.2 | 158.9 | <0.001 |
| Internal medicine | 1889 | 66.3 | | | 1294 | 66.0 | | |
| Surgery | 1266 | 66.5 | | | 955 | 68.9 | | |
| Gynaecology & Obstetrics | 284 | 66.7 | | | 391 | 64.1 | | |
| Paediatrics | 351 | 68.0 | | | 483 | 77.0 | | |
| Other | 1755 | 59.8 | | | 987 | 57.4 | | |
| Years of experience | | | | | | | | |
| 1-4 | 2141 | 61.0 | 55.0 | <0.001 | 2006 | 64.2 | 35.2 | <0.001 |
| 5-10 | 1920 | 68.5 | | | 1148 | 71.9 | | |
| 11-20 | 1069 | 69.7 | | | 750 | 68.2 | | |
| ≥21 | 833 | 64.4 | | | 635 | 63.0 | | |
| Working time | | | | | | | | |
| 0-2 h | 87 | 53.0 | 26.1 | <0.001 | 32 | 61.5 | 67.9 | <0.001 |
| 2-4 h | 152 | 60.3 | | | 71 | 59.2 | | |
| 4-6 h | 165 | 62.5 | | | 53 | 46.9 | | |
| 6-8 h | 2367 | 63.7 | | | 1941 | 62.9 | | |
| >8 h | 3192 | 67.2 | | | 2442 | 70.7 | | |
| Direct contact with patients | | | | | | | | |
| 0-2 h | 72 | 40.0 | 69.8 | <0.001 | 52 | 49.1 | 51.7 | <0.001 |
| 2-4 h | 127 | 53.6 | | | 144 | 57.4 | | |
| 4-6 h | 474 | 63.5 | | | 330 | 57.4 | | |
| 6-8 h | 5290 | 66.3 | | | 4013 | 68.1 | | |

**Characteristics of perpetrators and behavioral response of victims**

Patients' relatives were the main perpetrators of WPV in tertiary hospitals (83.1%) and county-level hospitals (85.2%). Most of the nurses were attacked in the ward, and in the shift during the day. More than 60% of the victims from tertiary hospitals and county-level hospitals coped with incidents of violence with tolerance, patience and understanding (Table 4).

For peer review only

**Table 4** Characteristics of perpetrators and behavioral response of victims

| | Tertiary hospitals(n=5963) | | County-level hospitals(n=4539) | |
|---|---|---|---|---|
| | n | % | n | % |
| Attack time | | | | |
| Day shift | 3558 | 59.7 | 2920 | 64.3 |
| Night shift | 1402 | 23.5 | 1195 | 26.4 |
| After work | 1003 | 16.8 | 424 | 9.3 |
| Attack site | | | | |
| Outpatient clinic | 388 | 6.5 | 351 | 7.7 |
| Ward | 2754 | 46.2 | 1933 | 42.6 |
| Doctor's office | 198 | 3.3 | 251 | 5.5 |
| Nurse's office or station | 1372 | 23.0 | 1291 | 28.5 |
| Treatment room | 127 | 2.1 | 162 | 3.6 |
| Others | 1124 | 18.9 | 551 | 12.1 |
| When the violent incident took place: | | | | |
| All alone | 1698 | 28.5 | 1319 | 29.1 |
| Other colleagues on the scene | 4265 | 71.5 | 3220 | 70.9 |
| Perpetrators * | | | | |
| Patients | 1784 | 35.9 | 1212 | 26.7 |
| Patients' relatives | 4131 | 83.1 | 3862 | 85.2 |
| Visitors | 748 | 15.0 | 573 | 12.6 |
| Others | 99 | 2.0 | 96 | 2.1 |
| Gender of the perpetrators* | | | | |
| Male | 3988 | 81.2 | 3380 | 83.1 |
| Female | 2461 | 50.1 | 1917 | 47.1 |
| Age group of the perpetrators (years)* | | | | |
| ≤20 | 258 | 5.1 | 258 | 5.7 |
| 21-30 | 1454 | 28.8 | 1624 | 35.9 |
| 31-40 | 2693 | 53.3 | 2263 | 50.0 |
| 41-50 | 2220 | 44.0 | 1529 | 33.8 |
| 51-60 | 917 | 18.2 | 1011 | 22.3 |
| ≥61 | 445 | 8.8 | 668 | 14.8 |
| Behavioral response to WPV * | | | | |
| Tolerance and avoidance | 3167 | 64.1 | 2601 | 63.3 |
| Patience and understanding | 2748 | 55.7 | 2415 | 58.8 |
| Give tit for tat | 80 | 1.6 | 36 | 0.9 |
| Try to explain before resorting to force | 438 | 8.9 | 310 | 7.5 |
| Ask colleagues for help | 940 | 19.0 | 537 | 13.1 |
| Turn to the leader or security staff for help | 1899 | 38.5 | 1334 | 32.5 |
| Ask for help from other patients and relatives | 287 | 5.8 | 165 | 4.0 |
| Call the police | 754 | 15.3 | 573 | 14.0 |
| Others | 166 | 3.4 | 72 | 1.8 |

* represents multiple choice

For peer review only

**Risk factors associated with WPV against nurses**

Participants' age, department, years of experience and length of time in direct contact with patients were risk factors associated with WPV against nurses in different levels of hospitals. Binary logistic regression analysis stated clearly that less than 8 hours of working time is a protective factor against WPV for nurses in the county-level hospitals. In contrast, the nurses of emergency department were 2.993 (95%CI, 2.364 to 3.789) times more likely to experience WPV in the tertiary hospitals and 3.387 times (95% CI, 2.648 to 4.332) in the county-level hospitals compared with the nurses who worked in other departments. It is surprising that the nurses with 5-10 years of experience were at the highest risk of violence in the county-level hospitals, while those with 11-20 years of experience were at the highest risk of violence in the tertiary hospitals in our study. Furthermore, the results also showed that, the longer the treatment time is, the greater the likelihood of violence is in the county-level hospitals (Table 5).

## DISCUSSION

Previous studies have found that WPV exists in hospitals with common epidemic regularity, and there are also differences in some aspects. [20-24] In the last 12 months, the total incidence rate of WPV was 65.8%, higher than 64.48% which has been reported.[25] This study demonstrated that WPV in China, compared with other countries, is relatively high. [26-29] Moreover, we found that WPV towards nurses is more frequent, including physical violence (11.8%), verbal violence (64.9%) and sexual harassment (3.9%). This may be related to the fact that the nurses often directly contact with patients and their relatives in their daily work. The finding also indicated that the nurses of the county-level hospitals are more likely to experience WPV than the tertiary hospitals, which may be related to the level of

For peer review only

**Table 5** Risk factors associated with WPV against nurses in hospitals, binary logistic results [*]

| Variable name | | Tertiary hospitals (n=9142) | | | County-level hospitals(n=6828) | | |
|---|---|---|---|---|---|---|---|
| | | Adjusted OR | 95%CI | *P*-value | Adjusted OR | 95%CI | *P*-value |
| Age group (years) | ≥51 | 1.0 | reference | 0.044 | 1.0 | reference | 0.027 |
| | ≤30 | 1.500 | (1.092-2.061) | 0.012 | 1.606 | (1.115-2.314) | 0.011 |
| | 31-50 | 1.387 | (1.036-1.855) | 0.028 | 1.551 | (1.120-2.146) | 0.008 |
| Department | Other | 1.0 | reference | <0.001 | 1.0 | reference | <0.001 |
| | Emergency department | 2.993 | (2.364-3.789) | <0.001 | 3.387 | (2.648-4.332) | <0.001 |
| | Internal medicine | 1.313 | (1.178-1.463) | <0.001 | 1.408 | (1.227-1.615) | <0.001 |
| | Surgery | 1.341 | (1.187-1.514) | <0.001 | 1.644 | (1.414-1.912) | <0.001 |
| | Gynaecology and Obstetrics | 1.322 | (1.065-1.641) | <0.001 | 1.268 | (1.044-1.539) | 0.016 |
| | Paediatrics department | 1.433 | (1.172-1.753) | <0.001 | 2.391 | (1.934-2.956) | <0.001 |
| Years of experience | 1-4 | 1.0 | reference | <0.001 | 1.0 | reference | <0.001 |
| | 5-10 | 1.426 | (1.268-1.604) | <0.001 | 1.479 | (1.277-1.712) | <0.001 |
| | 11-20 | 1.627 | (1.358-1.951) | <0.001 | 1.300 | (1.045-1.618) | 0.018 |
| | ≥21 | 1.368 | (1.131-1.656) | 0.001 | 1.192 | (0.949-1.498) | 0.131 |
| Working time (hours) | >8h | | | | 1.0 | reference | <0.001 |
| | 0-2h | | | | 0.852 | (0.469-1.548) | 0.599 |
| | 2-4h | | | | 0.762 | (0.507-1.145) | 0.191 |
| | 4-6h | | | | 0.492 | (0.329-0.735) | 0.001 |
| | 6-8h | | | | 0.720 | (0.647-0.800) | <0.001 |
| Direct contact with patients (hours) | 0-2h | 1.0 | reference | <0.001 | 1.0 | reference | <0.001 |
| | 2-4h | 1.832 | (1.230-2.728) | 0.003 | 1.201 | (0.744-1.938) | 0.454 |
| | 4-6h | 2.722 | (1.939-3.819) | <0.001 | 1.220 | (0.785-1.895) | 0.377 |
| | 6-8h | 3.054 | (2.247-4.152) | <0.001 | 1.710 | (1.134-2.580) | 0.011 |

[*] This analysis used data from 44 public tertiary hospitals and 90 county-level public hospitals in China.

For peer review only

education of patients and their relatives in the county-level hospitals. In addition, the proportion of agricultural population is significant in China which is convenient for these people to go to the county-level hospitals for medical treatment, increasing the likelihood of WPV. In short, nurses are more likely to suffer a high risk of WPV in the healthcare sector. [30-36]

Our surveys also revealed that the respondents of 30 years old or younger have a higher risk of WPV in the tertiary hospitals and the county-level hospitals.This phenomenon may attribute to the fact that they do their job not long enough, lacking work experience and communication skills. Therefore, they need to continuously practice their skills to decrease the odds of making mistakes. Or because most of the nurses were single children, when being criticized by the patients and their relatives, they are very easy to get angry, increasing the possibility of WPV. In contrast, the nurses who are 51 years old or older experienced a lower risk of WPV, which may be related with the Chinese traditional culture and inherent cognition and respect for the elderly, a traditional virtue of the Chinese nation for thousands of years.[37]

As shown in our surveys, the levels of risk of WPV is not the same in different departments. Substantial studies have found that emergency department was more likely to have a greater risk of violence with high incidence, followed by pediatrics department. [17, 38] This increased risk may be due to the fact that the emergency department has been facing the most serious patients in complex situations, such as traffic accidents, food poisoning, and even patients with alcoholism. Meanwhile, patients' relatives may be too worried about the patients, once the information between nurses and patients is not equally shared, and the communication and expression ability of nurses is insufficient, some unnecessary conflicts may emerge. In addition, pediatrics is another high-risk violence as a special department. In China, under the age of 14 all belong to the scope of pediatrics as the pediatric age

For peer review only

range is different in countries around the world. The high risk of violence in pediatrics department may be that the kids are mostly the only child so that the parents overindulge their children. This situation may make nurses' job more difficult and increase the possibility of WPV. Therefore, in order to reduce violent incidents, hospitals and patients should make joint efforts. On the one hand, hospitals need to improve the quality of nurses, who should show empathy and can understand each other. On the other hand, patients and their relatives should also show respect to others and understand the nurses' work is complex and professional. Medical staff and patients with mutual understanding can help alleviate the physician-patient relationship and achieve harmony.

This study also demonstrated that years of experience and the long-time direct contact with patients are risk factors of WPV. The process of medical treatment, the waiting time for patients, the service attitude of nurses and other aspects need to be improved, so as to enhance the satisfaction of patients and reduce the incidence of conflicts.

The characteristics of the perpetrators are equally noteworthy. In the present study, it is shown that the perpetrators are mainly family members of patients, followed by patients. The perpetrators are males dominately, concentrated in the age group from 31 to 50, with an inverted "U" shape distribution. The ward is the main attack site in hospitals, accounting for 46.2% and 42.6% in the tertiary hospitals and the county-level hospitals, respectively. It is very necessary to strengthen the management of the ward. For example, surveillance camera and alarm equipment are installed in the ward corridor of hospitals, and the lights are bright enough in the work area during the night, and so on.

Although most of patients and patients' relatives are very virtuous, there are also a few who have impure motives. In order to seek economic compensation, these people intentionally disrupt the normal medical order, and even pose threat to the personal safety of medical staff and other patients. In China,

For peer review only

a few years ago, the cost of " Yi Nao " was very low. The patients and their relatives may use some illegal organizations to ask the hospitals for compensation directly, rather than take the normal legal procedure. In order to ensure the normal medical order and their reputation, the hospitals often make concessions by meeting some unreasonable requirements to avoid troubles, which has further contributed to the arrogance of those resorting to "Yi Nao". Fortunately, " Yi Nao" was officially passed into the criminal law in November 2015. It means that " Yi Nao " has hazarded the normal medical order and gives a clear legal support in the conviction and sentencing. It is known that the negative media coverage has a huge impact on the physician-patient relationship. Therefore, we suggest that the government should supervise the media to ensure the accuracy and authenticity of the media coverage. We also advise hospitals to strengthen the training and management of nurses to decrease the physical and psychological damages to them.

The coping styles against WPV for victims are mainly tolerance, avoidance, patience and understanding in the tertiary hospitals and the county-level hospitals. This may be due to a culture of tolerance toward violence in China. Most of nurses turned to leaders or security personnel of the hospitals for help when they experienced WPV. Thus, the organization’s support and care may have a potential significance for reducing the harm and influence of WPV against nurses.

In general, WPV can be prevented, according to the World Health Organization's report on violence and health.[18] Hereby, we recommend that tertiary hospitals and county-level hospitals develop training against violence for employees according to their actual situation, to reduce the occurrence of violence.

For peer review only

## CONCLUSIONS

This study is a large sample of WPV for tertiary hospitals and county-level hospitals in China. The study demonstrated that the high incidence rate of violence is the main feature, and occupational safety is insufficient. Frequent occurrence of WPV in the emergency department and the pediatrics department is also a remarkable feature. WPV of hospitals, as an occupational health hazard, has a great threat to the wellbeing of healthcare workers.

**Acknowledgements**

The authors thank nurses, managers and Chinese Hospital Association for their assistance and support for this project. They also acknowledgements Xiaoli Jia and Min Ha for their assistance in the survey.

**Contributors**

Lei Shi and Lihua Fan designed the study. Danyang Zhang, Chenyu Zhou, Tianjun Hao, Xiangwen Peng , Yi Mu and Yuzhen Han collected data. Lei Shi, Libin Yang, Tao sun, Lei Gao and Wenhui Liu analysed the data. Lei Shi, Danyang Zhang, Chenyu Zhou and Lihua Fan drafted the manuscript. Lei Shi, Libin Yang, and Lihua Fan revised the manuscript.

**Funding**

This study was founded by the Natural Science Foundation of China, grant number 71473063.

**Competing interests** The authors declare no conflict of interest exist.

**Ethical approval**

Ethical approval to undertake this study was granted by the Research Ethics Committee of Harbin Medical University in March 2014.

**Data sharing statement** No additional data are available.

For peer review only

**REFERENCES**

1 Ramacciati N, Ceccagnoli A, Addey B. Violence against nurses in the triage area: An Italian qualitative study. *Int Emerg Nurs* 2015;23(4):274-280.

2 Lin YH, Liu HE. The impact of workplace violence on nurses in South Taiwan. *Int J Nurs Stud* 2005; 42:773–778.

3 Atawneh FA, Zahid MA, al-Sahlawi KS, Shahid, *et al*. Violence against nurses in hospitals: Prevalence and effects. *Brit J Nurs* 2003;12:102–107.

4 Pai DD, Lautert L, Souza SBCD, *et al*. Violence, Burnout and Minor Psychiatric Disorders in Hospital Work. *Rev Esc Enferm USP* 2015;49(3):457-64.

5 Martino VD. Workplace violence in the health sector.Country case studies Brazil, Bulgaria, Lebanon

Portugal, South Africa, Thailand and an additional Australian study. *Brasil* 2002.

6 Fute M, Mengesha ZB, Wakgari N, *et al*. High prevalence of workplace violence among nurses working at public health facilities in Southern Ethiopia. *BMC Nurs* 2015;14:9.

7 Mihyun P, Sung-Hyun C, Hyun-Ja H. Prevalence and Perpetrators of Workplace Violence by Nursing Unit and the Relationship Between Violence and the Perceived Work Environment. *J Nurs Scholarsh* 2014;47(1):87-95.

8 Al-Omari H. Physical and verbal workplace violence against nurses in Jordan. *Int Nurs Rev* 2015;62(1):111–118.

9 Hogarth KM, Beattie J, Morphet J. Nurses' attitudes towards the reporting of violence in the emergency department. *Australas Emerg Nurs J* Published Online First: 23 May 2015. doi: 10.1016/j.aenj.2015.03.006.

For peer review only

10 Shen HC, Cheng, Y, Tsai PJ, *et al*. Occupational stress in nurses in psychiatric institutions in Taiwan. *J Occup Health* 2005;47:218–225.

11 Pompeii LA, Schoenfisch AL, Lipscomb HJ, *et al*. Physical assault, physical threat, and verbal abuse perpetrated against hospital workers by patients or visitors in six U.S. hospitals. *Am J Ind Med* 2015;58(11):194-204.

12 Hahn S, Muller M, Hantikainen V, *et al*. Risk factors associated with patient and visitor violence in general hospitals: results of a multiple regression analysis. *Int J Nurs Stud* 2013;50(3):374-85.

13 Pan Y, Yang XH, He JP, *et al*. To be or not to be a doctor, that is the question: a review of serious incidents of violence against doctors in China from 2003–2013. *J public health* 2015;23(2):111-116.

14 Jia XL, Zhou HZ, Zhao Y, *et al*. Investigation on hospital violence during 2003 to 2012 in China. *Chinese Hospitals* 2014;18(3):1-3.

15 Liu L, Yang YL, Wang ZY, *et al*. Prevalence and Positive Correlates of Posttraumatic Stress Symptoms among Chinese Patients with Hematological Malignancies: A Cross-Sectional Study[. *Plos One* 2015;10(12).

16 Wang X, Jing ZR, Zhao BL, *et al*. Survey on workplace violence and job burnout among nurses. *J Nurs Sci* 2015;(12):15-17.

17 Hahn SZA, Needham IEA. Patient and visitor violence in general hospitals: a systematic review of the literature. *Aggression Violent Behav* 2008;13:431–41.

18 Chen ZH, Wang SY. Psychiatric hospital and general hospital workplace violence study comparison. *Chinese Journal of Public Health* 2004;20:1316–1317.

19 ILO/ICN/WHO/PSI. Framework Guidelines for Addressing Workplace Violence in the Health Sector.Geneva: ILO/ICN/WHO/PSI Joint Programme on Workplace Violence in the Health Sector

For peer review only

2002:4-132.

20 Laschinger HK, Grau AL. The influence of personal dispositional factors and organizational resources on workplace violence, burnout, and health outcomes in new graduate nurses: A cross-sectional study. *Int J Nurs Stud* 2012;49:282–291.

21 Kitaneh M, Hamdan M. Workplace violence against physicians and nurses in Palestinian public hospitals: A cross-sectional study. *BMC Health Serv Res* 2012;12:469.

22 Carter R. High risk of violence against nurses. *Nurs Manag* 2000;6:5.

23 Merecz D, Rymaszewska J, Moscicka A, *et al*. Violence at the workplace-a questionnaire survey of nurses. *Eur Psychiatry* 2006;21:442–450.

24 Ferns T. Under-reporting of violent incidents against nursing staff. *Nurs Stand* 2006;20(40) :41–45.

25 Chen ZH, Wang SY, Jing CX. Investigation on workplace violence phenomenon of two hospitals in Guangzhou. *Chinese Journal of Preventive Medicine* 2003;37:358–360.

26 Zampieron A, Galeazzo M, Turra S, et al. Perceived aggression towards nurses: study in two Italian health institutions. *J Clin Nurs* 2010;19:2329–41.

27 Gerberich SG, Church TR, McGovern PM, *et al.* An epidemiological study of the magnitude and consequences of work related violence: the Minnesota Nurses' Study. *Occup Environ Med* 2004;61: 495–503.

28 Franz S, Zeh A, Schablon A, *et al*. Aggression and violence against health care workers in Germany–a cross sectional retrospective survey. *BMC Health Serv Res* 2010;10:51.

29 Hahn S, Muller M, Needham I, *et al.* Factors associated with patient and visitor violence

For peer review only

experienced by nurses in general hospitals in Switzerland: a cross-sectional survey. *J Clin Nurs* 2010; 19:3535–46.

30 Hesketh KL, Duncan SM, Estabrooks CA, *et al*. Workplace violence in Alberta and British Columbia hospitals. *Health Policy* 2003;63:311-21.

31 Findorff MJ, McGovern PM, Wall M, *et al*. Risk factors for work related Violence in a health care organization. *Inj Prev* 2004;10(5):296–302.

32 Magin PJ, May J, McElduff P. Occupational violence in general practice: a whole-of-practice problem. Results of a cross-sectional study. *Australian Health Review* 2011;35(1):75–80.

33 Hegney D, Plank A, Parker V. Workplace violence in nursing in Queensland, Australia: A self-reported study. *Int J Nurs Pract* 2003;9:261–268.

34 Kwok RP, Law YK, Li KE, *et al*. Prevalence of workplace violence against nurses in Hong Kong. *Hong Kong Med J* 2006;12:6–9.

35 Hegney D, Plank A, Parker V. Workplace violence in nursing in Queensland, Australia: A self-reported study. *Int J Nurs Pract* 2003;9:261–268.

36 Whittington R, Wykes T. An evaluation of staff training in psychological techniques for the management of patient aggression. *J Clin Nurs* 1996;5(4):257–261.

37 Sun T, Wang S, Wang N, *et al*. Epidemiological characteristics and variance analysis of workplace violence in public hospitals. *Practical Preventive Medicine* 2014;21(4):478-480.

38 Hodgson MJ, Reed R, Craig T, *et al*. Violence in healthcare facilities:lessons from the Veterans Health Administration. *J Occup Environ Med* 2004;46(11):1158–1165.

For peer review only

Checklist of ***cross-sectional studies***

| | Page number, line number |
|---|---|
| **Title** | 1, 4-6 |
| **Keywords** | 1, 44 |
| **Abstract** | 2, 4-45 |
| **Strengths and limitations of this study** | 2, 48-58 |
| **Introduction** | |
| Background | 3, 4-57 |
| Objectives | 4, 6-12 |
| **Methods** | |
| Study design | 4, 19-49 |
| Setting | 4, 27-34 |
| Participants | 4,44-49 |
| Questionnaire | 5, 14-39 |
| Data measurement | 5, 44 |
| Study size | 5,4-9 |
| Quantitative variables | 5,46-58 |
| Ethical considerations | 6, 16-22 |
| **Results** | |
| Participants | 6, 29-58; 7, 3-50 |
| Descriptive data | 7, 55-57; 8-17 |
| Outcome data | 9,22-58;10,4-42 |
| Main results | 11; 12 |
| Other analyses | 9, 45-57; 10 |
| **Discussion** | |
| Key results | 11,40-58; 13,4-14 |
| Interpretation | 13, 16-57; 14, 4-34 |
| Discuss the external validity of the study results | 14, 37-57; 15 |
| **Conclusions** | 16, 4-16 |
| **Other information** | |
| Acknowledgements | 16, 21-26 |
| Contributors | 16, 29-39 |
| Funding | 16, 41-44 |
| Competing interests | 16,47 |
| Ethical approval | 16, 49-54 |
| | 16, 57 |

For peer review only

BMJ Open

# A cross-sectional study on the prevalence and associated risk factors for workplace violence against Chinese nurses

| | |
|---|---|
| Journal: | *BMJ Open* |
| Manuscript ID | bmjopen-2016-013105.R1 |
| Article Type: | Research |
| Date Submitted by the Author: | 21-Dec-2016 |
| Complete List of Authors: | Shi, Lei; School of Public Health, Harbin Medical University, Department of Health Management<br>Zhang, Danyang; The First Affiliated Hospital of Harbin Medical University, Operating Section<br>Zhou, Chenyu; School of Public Health, Harbin Medical University, Department of Health Management<br>Yang, Libin; School of Public Health, Harbin Medical University, Department of Medical Education<br>Sun, Tao; School of Public Health, Harbin Medical University, Department of Health Management<br>Hao, Tianjun; The Second Affiliated Hospital of Harbin Medical University, Department of Scientific Research<br>Peng, Xiangwen ; The First Affiliated Hospital of Harbin Medical University, Harbin, Operating Section<br>Gao, Lei; School of Public Health, Harbin Medical University, Department of Health Management<br>Liu, Wenhui; School of Public Health, Harbin Medical University, Department of Medical Education<br>Mu, Yi; Beijing Children's Hospital, Capital Medical University, Department of Customer Service<br>Han, Yuzhen; The Fourth Affiliated Hospital of Harbin Medical University, Department of Medical Disputes<br>Fan, Lihua; School of Public Health, Harbin Medical University, Department of Health Management |
| <b>Primary Subject Heading</b>: | Health services research |
| Secondary Subject Heading: | Health policy, Medical management, Nursing |
| Keywords: | workplace violence (WPV), nurses, general and public hospitals, risk factors |
| | |

SCHOLARONE™
Manuscripts

# A cross-sectional study on the prevalence and associated risk factors of workplace violence against Chinese nurses

Lei Shi, [1] Danyang Zhang,[2] Chenyu Zhou,[1] Libin Yang,[3] Tao Sun,[1] Tianjun Hao,[4] Xiangwen Peng,[2] Lei Gao,[1] Wenhui Liu,[3] Yi Mu,[5] Yuzhen Han, [6] Lihua Fan[1]

**Correspondence to:** Lihua Fan, School of Public Health, Harbin Medical University, 157 Baojian Road, Nangang District, Harbin 150081, China; E-mail: lihuafan@126.com;

[1]Department of Health Management, School of Public Health, Harbin Medical University, Harbin, China

[2] Operating Section, The First Affiliated Hospital of Harbin Medical University, Harbin, China

[3]Department of Medical Education, School of Public Health, Harbin Medical University, Harbin, China

[4]Department of Scientific Research, The Second Affiliated Hospital of Harbin Medical University, Harbin, China

[5]Department of Medical Matters, Beijing Children's Hospital, Capital Medical University, Beijing, China

[6] Department of Medical Matters, The Fourth Affiliated Hospital of Harbin Medical University, Harbin, China

**Keywords:** workplace violence (WPV); nurses; general public hospitals; risk factors

For peer review only

**Abstract**

**Objectives:** The purpose of the present study was to explore the epidemic characteristics of workplace violence that Chinese nurses of tertiary hospitals and county-level hospitals have encountered in the past 12 months, to identify and to analyze the risk factors, thus providing the basis for future preventive strategies.

**Design:** A cross-sectional sampling survey was conducted to collect the data.

**Participants:** A total of 9142 nurses from 44 tertiary hospitals and 6828 nurses from 90 county-level hospitals located in 16 provinces of China.

**Results:** The total prevalence of workplace violence was 65.8% (10502/15970), of which the verbal violence accounted for 64.9%, physical violence and sexual harassment accounted for 11.8% and 3.9% respectively. Frequent workplace violence mainly occurred in the emergency department and the pediatrics department. Respondents claimed that patients' relatives were the main perpetrators in tertiary hospitals (83.1%) and county-level hospitals (85.2%). Logistic regression analysis showed that participants' age, department, years of experience and the nurses direct contact with patients were common risk factors in different levels of hospitals.

**Conclusions**: The high incidence of workplace violence is the main feature. Workplace violence among nurses is an important issue that cannot be overlooked. A series of laws, policies, and interventions should be implemented to cope with workplace violence.

**Strengths and limitations of this study**

First, we collected data over the past 12 months about whether nurses had experienced violence, so the recall bias may exist. Second, we conducted a study of tertiary hospitals and county-level hospitals, but did not study specialized hospitals. Third, further researches on the psychological harm of WPV are

For peer review only

necessary to be carried out.

**BACKGROUND**

Workplace violence (WPV) that towards health professionals in the health service systems has been recognized as a global public health issue and caused broad attention in the worldwide.[1-4] Previous studies have indicated that health professionals are at the highest risk for experiencing violence in all work environments.[2] Besides, the incidence rate of WPV among healthcare workers in various countries are different, for instance, it was 67.2% in Australia, 46.7% in Brazil, 61% in South Africa, and 37% in Portugal.[5]

On this basis, nurses are more likely to experience WPV than other staff in hospitals. During the past 12 months, incidence rate of physical violence in Ethiopia,[6] South Korea[7] and Jordan[8] ranged from 18.22% to 52.8%, verbal abuse from 63.8% to 89.58% and sexual harassment from 13.02% to 19.7%. The main concentration of WPV events is in the emergency ward and psychiatric department of hospitals. [9-10] Research on the nurses of these departments in the US,[11] Switzerland[12] and Taiwan[2] has demonstrated a higher prevalence of WPV.

In China, WPV in hospitals has been gradually increasing over the past years. From 2000 to 2012, the physician-patient conflicts presented a growing trend, the number of doctors who were killed by patients went up to 14, and more people were injured. [13] According to the report of Chinese Hospital Association, the proportion of hospitals experiencing violence has risen from 90% (2008) to 96% (2012); and the prevalence of sexual harassment also increased year by year.[14]

Although the violent incidents in hospitals directly harm the healthcare workers and hospitals, the ultimate victims are still patients.[15] WPV affects the normal order and reputation of the hospitals, and threatens the personal safety of healthcare workers and patients.[16] Moreover, violent incidents also have

For peer review only

a negative impact on the psychological aspect of the healthcare workers, which may lead to the phenomenon that healthcare workers do not hope their children to be engaged in medical work.

The purpose of the present study was to explore the distribution characteristics of WPV that Chinese nurses of tertiary hospitals and county-level hospitals have encountered in the last 12 months, to further identify and to analyze the risk factors, thus providing the basis for future preventive strategies.

## METHODS

A cross-sectional survey was designed based on the geographical location and the level of economic development in the East (Beijing, Tianjin, Hebei, Shandong, Guangdong, Heilongjiang, Liaoning), Middle (Shanxi, Henan, Anhui, Hunan) and West (Ningxia, Shannxi, Gansu, Sichuan, Chongqing) regions of China. According to a certain proportion, 120 nurses were extracted from department of Internal medicine, Surgery, 80 nurses were extracted from department of Emergency, Neurology, Obstetrics and Gynecology, Pediatrics, and 40 nurses were selected from department of Stomatology, Ophthalmology, Ear, Nose and Throat in each of tertiary hospital. A total of 10560 questionnaires were distributed in 44 tertiary hospitals. By the same method, the nurses were chosen by the 60, 40 and 20 from those departments at the county-level hospital. A total of 10800 questionnaires were distributed in 90 county-level hospitals.

The inclusion criteria for participants are as follows: the nurses engaged in clinical work should be with at least one year professional experience in hospitals and participated voluntarily. The permission was acquired from all 134 hospitals throughout the study.

The survey was conducted from December 2014 to January 2016. We obtained permission from

For peer review only

the managers and human resources departments of the hospitals. The researchers used a stratified sampling method to collect the data. Each participant had 2 days to accomplish the self-administered questionnaire. These data were kept confidential and used only for academic research. A total of 21360 questionnaires were handed out, and 15970 valid questionnaires were recovered (effective response rate=74.77%).

## QUESTIONNAIRE

We developed the questionnaire based on the following three aspects. First, we used the revised SOVES-G-R about the Survey of Violence Experienced by Staff (SOVES) from Hahn and colleagues to develop the questionnaire.[17] Then, we referenced a literature about a comparative study of workplace violence in psychiatric hospitals and general hospitals in China.[18] In addition, we also referenced the questionnaires of the ILO, ICN, WHO, and PSI joint program for measuring WPV. [19] We obtained permission to use these questionnaires. Futhermore, we invited 18 healthcare-related experts to assess the accuracy, comprehensiveness and sensitivity about all of the items in the questionnaire. The questionnaire was pre-tested among a total of 367 nurses from 3 public tertiary hospitals and 2 public county-level hospitals. Then, the questionnaire was further revised. After repeated measurements, the reliability and validity of questionnaires were 0.86 and 0.84, respectively.

## DATA ANALYSIS

EpiData version 3.1 was used to establish a database. After finishing all the surveys, data was checked by trained personnel to ensure data accuracy. IBM SPSS19.0 and Excel were used for statistical analysis of the relevant quantitative data. Univariate analysis and chi-square tests ($\chi^2$) were used to

For peer review only

analyze the relationship between the demographic characteristics of respondents and the prevalence of WPV. We test whether the nurses suffering from hospital WPV as the Dependent Variable, and the variables with statistical and clinical practice significance as the Covariates in logistic regression analysis. Binary logistic regression analyses on the risk factors of WPV were conducted among the nurses in public tertiary hospitals and county-level hospitals. Odds ratios (OR) and 95% confidence intervals (CI) were calculated; $\alpha=0.05$ was the level of a test; and $P<0.05$ was considered statistically significant.

**ETHICAL CONSIDERATIONS**

We obtained consent from each hospital in the entire research processes. All of the participants were given the informed consent before the survey, and the participants' personal information was kept confidential.

**RESULTS**

**Demographic characteristics of the participants**

A total of 15970 participants accord with our inclusion criteria. Of the 15970 respondents, most of them had finished their undergraduate studies, and the age group was concentrated on less than 30 years old, but there was a large difference in the proportion of gender (Table 1).

**Table 1** Demographic characteristics of the participants(N=15970)

| Characteristics | Tertiary hospitals (n=9142) | | County-level hospitals (n=6828) | | Total (n=15970) | |
|---|---|---|---|---|---|---|
| | n | % | n | % | n | % |
| Gender | | | | | | |
| Male | 234 | 2.6 | 143 | 2.1 | 377 | 2.4 |
| Female | 8908 | 97.4 | 6685 | 97.9 | 15593 | 97.6 |
| Age group (years) | | | | | | |
| ≤30 | 5131 | 56.1 | 4033 | 59.1 | 9164 | 57.4 |
| 31-50 | 3785 | 41.4 | 2611 | 38.2 | 6396 | 40.0 |

For peer review only

| | | | | | | |
|---|---|---|---|---|---|---|
| ≥51 | 226 | 2.5 | 184 | 2.7 | 410 | 2.6 |
| Education status | | | | | | |
| Below undergraduate | 3129 | 34.2 | 4161 | 60.9 | 7290 | 45.6 |
| Undergraduate | 5910 | 64.6 | 2663 | 39.0 | 8573 | 53.7 |
| Master or above | 103 | 1.1 | 4 | 0.1 | 107 | 0.7 |
| Marital status | | | | | | |
| Married | 5678 | 62.1 | 4293 | 62.9 | 9971 | 62.4 |
| Unmarried | 3342 | 36.6 | 2415 | 35.4 | 5757 | 36.1 |
| Divorced or Widowed | 122 | 1.3 | 120 | 1.7 | 242 | 1.5 |
| Professional title | | | | | | |
| Junior | 6249 | 68.4 | 4845 | 71.0 | 11094 | 69.5 |
| Intermediate | 2242 | 24.5 | 1587 | 23.2 | 3829 | 24.0 |
| Senior | 651 | 7.1 | 396 | 5.8 | 1047 | 6.5 |
| Employment form | | | | | | |
| Regular staff | 5077 | 55.5 | 3339 | 48.9 | 8416 | 52.7 |
| Temporary employee | 4065 | 44.5 | 3489 | 51.1 | 7554 | 47.3 |
| Average monthly income (RMB) | | | | | | |
| ≤3000 | 3747 | 41.0 | 4916 | 72.0 | 8663 | 54.3 |
| 3000-5000 | 4495 | 49.2 | 1867 | 27.3 | 6362 | 39.8 |
| 5000-10000 | 872 | 9.5 | 42 | 0.6 | 914 | 5.7 |
| >10000 | 28 | 0.3 | 3 | 0.1 | 31 | 0.2 |
| Department | | | | | | |
| Emergency | 514 | 5.6 | 522 | 7.6 | 1036 | 6.5 |
| Internal medicine | 2850 | 31.2 | 1962 | 28.7 | 4812 | 30.1 |
| Surgery | 1903 | 20.8 | 1386 | 20.3 | 3289 | 20.6 |
| Gynaecology and Obstetrics | 426 | 4.7 | 610 | 8.9 | 1036 | 6.5 |
| Paediatrics | 516 | 5.6 | 627 | 9.2 | 1143 | 7.2 |
| Others | 2933 | 32.1 | 1721 | 25.3 | 4654 | 29.1 |
| Years of experience | | | | | | |
| 1-4 | 3511 | 38.4 | 3124 | 45.7 | 6635 | 41.5 |
| 5-10 | 2803 | 30.7 | 1597 | 23.4 | 4400 | 27.6 |
| 11-20 | 1534 | 16.8 | 1099 | 16.1 | 2633 | 16.5 |
| ≥21 | 1294 | 14.1 | 1008 | 14.8 | 2302 | 14.4 |
| Working time | | | | | | |
| 0-2 h | 164 | 1.8 | 52 | 0.8 | 216 | 1.3 |
| 2-4 h | 252 | 2.8 | 120 | 1.8 | 372 | 2.3 |
| 4-6 h | 264 | 2.9 | 113 | 1.6 | 377 | 2.4 |
| 6-8 h | 3713 | 40.6 | 3087 | 45.2 | 6800 | 42.6 |
| >8 h | 4749 | 51.9 | 3456 | 50.6 | 8205 | 51.4 |
| Direct contact with patients | | | | | | |
| 0-2 h | 180 | 2.0 | 106 | 1.6 | 286 | 1.8 |
| 2-4 h | 237 | 2.6 | 251 | 3.7 | 488 | 3.0 |
| 4-6 h | 747 | 8.2 | 575 | 8.4 | 1322 | 8.3 |
| 6-8 h | 7978 | 87.2 | 5896 | 86.3 | 13874 | 86.9 |

For peer review only

**Frequency of WPV exposure in the last 12 months**

In the last 12 months, 10502 nurses have experienced WPV in the hospitals. The total prevalence of WPV was 65.8%, of which the verbal violence accounted for 64.9% (10369/15970), physical violence accounted for 11.8% (1892/15970), and sexual harassment accounted for 3.9% (630/15970). The prevalence of WPV in different kinds of hospitals is shown in Table 2.

**Table 2** Prevalence (%) of WPV exposure

| Type | Tertiary Hospitals | | | | | | County-level hospitals | | | | | |
|---|---|---|---|---|---|---|---|---|---|---|---|---|
| | Verbal violence | | Physical violence | | Sexual harassment | | Verbal violence | | Physical violence | | Sexual harassment | |
| | n | % | n | % | n | % | n | % | n | % | n | % |
| | 5875 | 64.3 | 1047 | 11.5 | 402 | 4.4 | 4494 | 65.8 | 845 | 12.4 | 228 | 3.3 |

**Prevalence of WPV in different demographic variables**

The nurses who have frequent contact with patients were more likely to experience WPV in the tertiary hospitals and county-level hospitals (Table 3). In the tertiary hospitals, there was a significant difference in the incidence rate of WPV among different professional titles ($\chi^2$=6.6, $p$<0.05). On the contrary, there was no significant difference in the county-level hospitals ($\chi^2$=1.5, $p$>0.05).

**Table 3** Characteristics and frequency distributions for WPV

| Characteristics | Tertiary hospitals (n=9142) | | | | County-level hospitals (n=6828) | | | |
|---|---|---|---|---|---|---|---|---|
| | n | % | $\chi^2$ | $p$ | n | % | $\chi^2$ | $p$ |
| Gender | | | | | | | | |
| Male | 143 | 61.1 | 1.8 | 0.181 | 85 | 59.4 | 3.2 | 0.072 |
| Female | 5820 | 65.3 | | | 4454 | 66.6 | | |
| Age | | | | | | | | |
| ≤30 | 3283 | 64.0 | 15.4 | <0.001 | 2679 | 66.4 | 15.6 | <0.001 |
| 31-50 | 2548 | 67.3 | | | 1762 | 67.5 | | |
| ≥51 | 132 | 58.4 | | | 98 | 53.3 | | |
| Education | | | | | | | | |
| Below undergraduate | 1958 | 62.6 | 15.7 | <0.001 | 2707 | 65.1 | 9.7 | 0.008 |
| Undergraduate | 3941 | 66.7 | | | 1829 | 68.7 | | |
| Master or above | 64 | 62.1 | | | 3 | 75.0 | | |
| Marital status | | | | | | | | |
| Married | 3813 | 67.2 | 27.8 | <0.001 | 2884 | 67.2 | 3.3 | 0.192 |
| Unmarried | 2065 | 61.8 | | | 1581 | 65.5 | | |
| Divorced or Widowed | 85 | 69.7 | | | 74 | 61.7 | | |

| | | | | | | | | |
|---|---|---|---|---|---|---|---|---|
| Professional title | | | | | | | | |
| Junior | 4022 | 64.4 | 6.6 | 0.037 | 3217 | 66.4 | 1.5 | 0.482 |
| Intermediate | 1607 | 71.7 | | | 1068 | 67.3 | | |
| Senior | 434 | 66.7 | | | 254 | 64.1 | | |
| Employment form | | | | | | | | |
| Regular staff | 3290 | 64.8 | 0.9 | 0.341 | 2213 | 66.3 | 0.1 | 0.733 |
| Temporary employee | 2673 | 65.8 | | | 2326 | 66.7 | | |
| Average monthly income (RMB) | | | | | | | | |
| ≤3000 | 2418 | 64.5 | 1.4 | 0.710 | 3275 | 66.6 | 0.3 | 0.955 |
| 3000-5000 | 2953 | 65.7 | | | 1233 | 66.0 | | |
| 5000-10000 | 574 | 65.8 | | | 29 | 69.0 | | |
| >10000 | 18 | 64.3 | | | 2 | 66.7 | | |
| Department | | | | | | | | |
| Emergency | 418 | 81.3 | 101.2 | <0.001 | 429 | 82.2 | 158.9 | <0.001 |
| Internal medicine | 1889 | 66.3 | | | 1294 | 66.0 | | |
| Surgery | 1266 | 66.5 | | | 955 | 68.9 | | |
| Gynaecology & Obstetrics | 284 | 66.7 | | | 391 | 64.1 | | |
| Paediatrics | 351 | 68.0 | | | 483 | 77.0 | | |
| Other | 1755 | 59.8 | | | 987 | 57.4 | | |
| Years of experience | | | | | | | | |
| 1-4 | 2141 | 61.0 | 55.0 | <0.001 | 2006 | 64.2 | 35.2 | <0.001 |
| 5-10 | 1920 | 68.5 | | | 1148 | 71.9 | | |
| 11-20 | 1069 | 69.7 | | | 750 | 68.2 | | |
| ≥21 | 833 | 64.4 | | | 635 | 63.0 | | |
| Working time | | | | | | | | |
| 0-2 h | 87 | 53.0 | 26.1 | <0.001 | 32 | 61.5 | 67.9 | <0.001 |
| 2-4 h | 152 | 60.3 | | | 71 | 59.2 | | |
| 4-6 h | 165 | 62.5 | | | 53 | 46.9 | | |
| 6-8 h | 2367 | 63.7 | | | 1941 | 62.9 | | |
| >8 h | 3192 | 67.2 | | | 2442 | 70.7 | | |
| Direct contact with patients | | | | | | | | |
| 0-2 h | 72 | 40.0 | 69.8 | <0.001 | 52 | 49.1 | 51.7 | <0.001 |
| 2-4 h | 127 | 53.6 | | | 144 | 57.4 | | |
| 4-6 h | 474 | 63.5 | | | 330 | 57.4 | | |
| 6-8 h | 5290 | 66.3 | | | 4013 | 68.1 | | |

**Characteristics of perpetrators and behavioral response of victims**

Patients' relatives were the main perpetrators of WPV in tertiary hospitals (83.1%) and county-level hospitals (85.2%). Most of the nurses were attacked in the ward, and in the shift during the day. More than 60% of the victims from tertiary hospitals and county-level hospitals coped with incidents of violence with tolerance, patience and understanding (Table 4).

For peer review only

**Table 4** Characteristics of perpetrators and behavioral response of victims

| | Tertiary hospitals(n=5963) | | County-level hospitals(n=4539) | |
|---|---|---|---|---|
| | n | % | n | % |
| Attack time | | | | |
| Day shift | 3558 | 59.7 | 2920 | 64.3 |
| Night shift | 1402 | 23.5 | 1195 | 26.4 |
| After work | 1003 | 16.8 | 424 | 9.3 |
| Attack site | | | | |
| Outpatient clinic | 388 | 6.5 | 351 | 7.7 |
| Ward | 2754 | 46.2 | 1933 | 42.6 |
| Doctor's office | 198 | 3.3 | 251 | 5.5 |
| Nurse's office or station | 1372 | 23.0 | 1291 | 28.5 |
| Treatment room | 127 | 2.1 | 162 | 3.6 |
| Others | 1124 | 18.9 | 551 | 12.1 |
| When the violent incident took place: | | | | |
| All alone | 1698 | 28.5 | 1319 | 29.1 |
| Other colleagues on the scene | 4265 | 71.5 | 3220 | 70.9 |
| Perpetrators * | | | | |
| Patients | 1784 | 35.9 | 1212 | 26.7 |
| Patients' relatives | 4131 | 83.1 | 3862 | 85.2 |
| Visitors | 748 | 15.0 | 573 | 12.6 |
| Others | 99 | 2.0 | 96 | 2.1 |
| Gender of the perpetrators* | | | | |
| Male | 3988 | 81.2 | 3380 | 83.1 |
| Female | 2461 | 50.1 | 1917 | 47.1 |
| Age group of the perpetrators (years)* | | | | |
| ≤20 | 258 | 5.1 | 258 | 5.7 |
| 21-30 | 1454 | 28.8 | 1624 | 35.9 |
| 31-40 | 2693 | 53.3 | 2263 | 50.0 |
| 41-50 | 2220 | 44.0 | 1529 | 33.8 |
| 51-60 | 917 | 18.2 | 1011 | 22.3 |
| ≥61 | 445 | 8.8 | 668 | 14.8 |
| Behavioral response to WPV * | | | | |
| Tolerance and avoidance | 3167 | 64.1 | 2601 | 63.3 |
| Patience and understanding | 2748 | 55.7 | 2415 | 58.8 |
| Give tit for tat | 80 | 1.6 | 36 | 0.9 |
| Try to explain before resorting to force | 438 | 8.9 | 310 | 7.5 |
| Ask colleagues for help | 940 | 19.0 | 537 | 13.1 |
| Turn to the leader or security staff for help | 1899 | 38.5 | 1334 | 32.5 |
| Ask for help from other patients and relatives | 287 | 5.8 | 165 | 4.0 |
| Call the police | 754 | 15.3 | 573 | 14.0 |
| Others | 166 | 3.4 | 72 | 1.8 |

* represents multiple choice

For peer review only

**Risk factors associated with WPV against nurses**

Participants' age, department, years of experience and length of time in direct contact with patients were risk factors associated with WPV against nurses in different levels of hospitals. Binary logistic regression analysis stated clearly that less than 8 hours of working time is a protective factor against WPV for nurses in the county-level hospitals. In contrast, the nurses of emergency department were 2.993 (95%CI, 2.364 to 3.789) times more likely to experience WPV in the tertiary hospitals and 3.387 times (95% CI, 2.648 to 4.332) in the county-level hospitals compared with the nurses who worked in other departments. It is surprising that the nurses with 5-10 years of experience were at the highest risk of violence in the county-level hospitals, while those with 11-20 years of experience were at the highest risk of violence in the tertiary hospitals in our study. Furthermore, the results also showed that, the longer the treatment time is, the greater the likelihood of violence is in the county-level hospitals (Table 5).

## DISCUSSION

Previous studies have found that WPV exists in hospitals with common epidemic regularity, and there are also differences in some aspects. [20-24] In the last 12 months, the total incidence rate of WPV was 65.8%, higher than 64.48% which has been reported.[25] This study demonstrated that WPV in China, compared with other countries, is relatively high. [26-29] Moreover, we found that WPV towards nurses is more frequent, including physical violence (11.8%), verbal violence (64.9%) and sexual harassment (3.9%). This may be related to the fact that the nurses often directly contact with patients and their relatives in their daily work. The finding also indicated that the nurses of the county-level hospitals are more likely to experience WPV than the tertiary hospitals, which may be related to the level of

For peer review only

**Table 5** Risk factors associated with WPV against nurses in hospitals, binary logistic results *

| Variable name | | Tertiary hospitals (n=9142) | | | County-level hospitals(n=6828) | | |
|---|---|---|---|---|---|---|---|
| | | Adjusted OR | 95%CI | *P*-value | Adjusted OR | 95%CI | *P*-value |
| Age group (years) | ≥51 | 1.0 | reference | 0.044 | 1.0 | reference | 0.027 |
| | ≤30 | 1.500 | (1.092-2.061) | 0.012 | 1.606 | (1.115-2.314) | 0.011 |
| | 31-50 | 1.387 | (1.036-1.855) | 0.028 | 1.551 | (1.120-2.146) | 0.008 |
| Department | Other | 1.0 | reference | <0.001 | 1.0 | reference | <0.001 |
| | Emergency department | 2.993 | (2.364-3.789) | <0.001 | 3.387 | (2.648-4.332) | <0.001 |
| | Internal medicine | 1.313 | (1.178-1.463) | <0.001 | 1.408 | (1.227-1.615) | <0.001 |
| | Surgery | 1.341 | (1.187-1.514) | <0.001 | 1.644 | (1.414-1.912) | <0.001 |
| | Gynaecology and Obstetrics | 1.322 | (1.065-1.641) | <0.001 | 1.268 | (1.044-1.539) | 0.016 |
| | Paediatrics department | 1.433 | (1.172-1.753) | <0.001 | 2.391 | (1.934-2.956) | <0.001 |
| Years of experience | 1-4 | 1.0 | reference | <0.001 | 1.0 | reference | <0.001 |
| | 5-10 | 1.426 | (1.268-1.604) | <0.001 | 1.479 | (1.277-1.712) | <0.001 |
| | 11-20 | 1.627 | (1.358-1.951) | <0.001 | 1.300 | (1.045-1.618) | 0.018 |
| | ≥21 | 1.368 | (1.131-1.656) | 0.001 | 1.192 | (0.949-1.498) | 0.131 |
| Working time (hours) | >8h | | | | 1.0 | reference | <0.001 |
| | 0-2h | | | | 0.852 | (0.469-1.548) | 0.599 |
| | 2-4h | | | | 0.762 | (0.507-1.145) | 0.191 |
| | 4-6h | | | | 0.492 | (0.329-0.735) | 0.001 |
| | 6-8h | | | | 0.720 | (0.647-0.800) | <0.001 |
| Direct contact with patients (hours) | 0-2h | 1.0 | reference | <0.001 | 1.0 | reference | <0.001 |
| | 2-4h | 1.832 | (1.230-2.728) | 0.003 | 1.201 | (0.744-1.938) | 0.454 |
| | 4-6h | 2.722 | (1.939-3.819) | <0.001 | 1.220 | (0.785-1.895) | 0.377 |
| | 6-8h | 3.054 | (2.247-4.152) | <0.001 | 1.710 | (1.134-2.580) | 0.011 |

* This analysis used data from 44 public tertiary hospitals and 90 county-level public hospitals in China.

For peer review only

education of patients and their relatives in the county-level hospitals. In addition, the proportion of agricultural population is significant in China which is convenient for these people to go to the county-level hospitals for medical treatment, increasing the likelihood of WPV. In short, nurses are more likely to suffer a high risk of WPV in the healthcare sector. [30-36]

Our surveys also revealed that the respondents of 30 years old or younger have a higher risk of WPV in the tertiary hospitals and the county-level hospitals.This phenomenon may attribute to the fact that they do their job not long enough, lacking work experience and communication skills. Therefore, they need to continuously practice their skills to decrease the odds of making mistakes. Or because most of the nurses were single children, when being criticized by the patients and their relatives, they are very easy to get angry, increasing the possibility of WPV. In contrast, the nurses who are 51 years old or older experienced a lower risk of WPV, which may be related with the Chinese traditional culture and inherent cognition and respect for the elderly, a traditional virtue of the Chinese nation for thousands of years.[37]

As shown in our surveys, the levels of risk of WPV is not the same in different departments. Substantial studies have found that emergency department was more likely to have a greater risk of violence with high incidence, followed by pediatrics department. [17, 38] This increased risk may be due to the fact that the emergency department has been facing the most serious patients in complex situations, such as traffic accidents, food poisoning, and even patients with alcoholism. Meanwhile, patients' relatives may be too worried about the patients, once the information between nurses and patients is not equally shared, and the communication and expression ability of nurses is insufficient, some unnecessary conflicts may emerge. In addition, pediatrics is another high-risk violence as a special department. In China, under the age of 14 all belong to the scope of pediatrics as the pediatric age

For peer review only

range is different in countries around the world. The high risk of violence in pediatrics department may be that the kids are mostly the only child so that the parents overindulge their children. This situation may make nurses' job more difficult and increase the possibility of WPV. Therefore, in order to reduce violent incidents, hospitals and patients should make joint efforts. On the one hand, hospitals need to improve the quality of nurses, who should show empathy and can understand each other. On the other hand, patients and their relatives should also show respect to others and understand the nurses' work is complex and professional. Medical staff and patients with mutual understanding can help alleviate the physician-patient relationship and achieve harmony.

This study also demonstrated that years of experience and the long-time direct contact with patients are risk factors of WPV. The process of medical treatment, the waiting time for patients, the service attitude of nurses and other aspects need to be improved, so as to enhance the satisfaction of patients and reduce the incidence of conflicts.

The characteristics of the perpetrators are equally noteworthy. In the present study, it is shown that the perpetrators are mainly family members of patients, followed by patients. The perpetrators are males dominately, concentrated in the age group from 31 to 50, with an inverted "U" shape distribution. The ward is the main attack site in hospitals, accounting for 46.2% and 42.6% in the tertiary hospitals and the county-level hospitals, respectively. It is very necessary to strengthen the management of the ward. For example, surveillance camera and alarm equipment are installed in the ward corridor of hospitals, and the lights are bright enough in the work area during the night, and so on.

Although most of patients and patients' relatives are very virtuous, there are also a few who have impure motives. In order to seek economic compensation, these people intentionally disrupt the normal medical order, and even pose threat to the personal safety of medical staff and other patients. In China,

For peer review only

a few years ago, the cost of " Yi Nao " was very low. The patients and their relatives may use some illegal organizations to ask the hospitals for compensation directly, rather than take the normal legal procedure. In order to ensure the normal medical order and their reputation, the hospitals often make concessions by meeting some unreasonable requirements to avoid troubles, which has further contributed to the arrogance of those resorting to "Yi Nao". Fortunately, " Yi Nao" was officially passed into the criminal law in November 2015. It means that " Yi Nao " has hazarded the normal medical order and gives a clear legal support in the conviction and sentencing. It is known that the negative media coverage has a huge impact on the physician-patient relationship. Therefore, we suggest that the government should supervise the media to ensure the accuracy and authenticity of the media coverage. We also advise hospitals to strengthen the training and management of nurses to decrease the physical and psychological damages to them.

The coping styles against WPV for victims are mainly tolerance, avoidance, patience and understanding in the tertiary hospitals and the county-level hospitals. This may be due to a culture of tolerance toward violence in China. Most of nurses turned to leaders or security personnel of the hospitals for help when they experienced WPV. Thus, the organization's support and care may have a potential significance for reducing the harm and influence of WPV against nurses.

In general, WPV can be prevented, according to the World Health Organization's report on violence and health.[18] Hereby, we recommend that tertiary hospitals and county-level hospitals develop training against violence for employees according to their actual situation, to reduce the occurrence of violence.

For peer review only

## CONCLUSIONS

This study is a large sample of WPV for tertiary hospitals and county-level hospitals in China. The study demonstrated that the high incidence rate of violence is the main feature, and occupational safety is insufficient. Frequent occurrence of WPV in the emergency department and the pediatrics department is also a remarkable feature. WPV of hospitals, as an occupational health hazard, has a great threat to the wellbeing of healthcare workers.

**Acknowledgements**

The authors thank nurses, managers and Chinese Hospital Association for their assistance and support for this project. They also acknowledgements Xiaoli Jia and Min Ha for their assistance in the survey.

**Contributors**

Lei Shi and Lihua Fan designed the study. Danyang Zhang, Chenyu Zhou, Tianjun Hao, Xiangwen Peng , Yi Mu and Yuzhen Han collected data. Lei Shi, Libin Yang, Tao sun, Lei Gao and Wenhui Liu analysed the data. Lei Shi, Danyang Zhang, Chenyu Zhou and Lihua Fan drafted the manuscript. Lei Shi, Libin Yang, and Lihua Fan revised the manuscript.

**Funding**

This study was founded by the Natural Science Foundation of China, grant number 71473063.

**Competing interests** The authors declare no conflict of interest exist.

**Ethical approval**

Ethical approval to undertake this study was granted by the Research Ethics Committee of Harbin Medical University in March 2014.

**Data sharing statement** No additional data are available.

For peer review only

## REFERENCES


1 Ramacciati N, Ceccagnoli A, Addey B. Violence against nurses in the triage area: An Italian qualitative study. *Int Emerg Nurs* 2015;23(4):274-280.

2 Lin YH, Liu HE. The impact of workplace violence on nurses in South Taiwan. *Int J Nurs Stud* 2005; 42:773–778.

3 Atawneh FA, Zahid MA, al-Sahlawi KS, Shahid, *et al*. Violence against nurses in hospitals: Prevalence and effects. *Brit J Nurs* 2003;12:102–107.

4 Pai DD, Lautert L, Souza SBCD, *et al*. Violence, Burnout and Minor Psychiatric Disorders in Hospital Work. *Rev Esc Enferm USP* 2015;49(3):457-64.

5 Martino VD. Workplace violence in the health sector.Country case studies Brazil, Bulgaria, Lebanon

Portugal, South Africa, Thailand and an additional Australian study. *Brasil* 2002.

6 Fute M, Mengesha ZB, Wakgari N, *et al*. High prevalence of workplace violence among nurses working at public health facilities in Southern Ethiopia. *BMC Nurs* 2015;14:9.

7 Mihyun P, Sung-Hyun C, Hyun-Ja H. Prevalence and Perpetrators of Workplace Violence by Nursing Unit and the Relationship Between Violence and the Perceived Work Environment. *J Nurs Scholarsh* 2014;47(1):87-95.

8 Al-Omari H. Physical and verbal workplace violence against nurses in Jordan. *Int Nurs Rev* 2015;62(1):111–118.

9 Hogarth KM, Beattie J, Morphet J. Nurses' attitudes towards the reporting of violence in the emergency department. *Australas Emerg Nurs J* Published Online First: 23 May 2015. doi: 10.1016/j.aenj.2015.03.006.


For peer review only

10 Shen HC, Cheng, Y, Tsai PJ, *et al*. Occupational stress in nurses in psychiatric institutions in Taiwan. *J Occup Health* 2005;47:218–225.

11 Pompeii LA, Schoenfisch AL, Lipscomb HJ, *et al*. Physical assault, physical threat, and verbal abuse perpetrated against hospital workers by patients or visitors in six U.S. hospitals. *Am J Ind Med* 2015;58(11):194-204.

12 Hahn S, Muller M, Hantikainen V, *et al*. Risk factors associated with patient and visitor violence in general hospitals: results of a multiple regression analysis. *Int J Nurs Stud* 2013;50(3):374-85.

13 Pan Y, Yang XH, He JP, *et al.* To be or not to be a doctor, that is the question: a review of serious incidents of violence against doctors in China from 2003–2013. *J public health* 2015;23(2):111-116.

14 Jia XL, Zhou HZ, Zhao Y, *et al*. Investigation on hospital violence during 2003 to 2012 in China. *Chinese Hospitals* 2014;18(3):1-3.

15 Liu L, Yang YL, Wang ZY, *et al*. Prevalence and Positive Correlates of Posttraumatic Stress Symptoms among Chinese Patients with Hematological Malignancies: A Cross-Sectional Study[. *Plos One* 2015;10(12).

16 Wang X, Jing ZR, Zhao BL, *et al*. Survey on workplace violence and job burnout among nurses. *J Nurs Sci* 2015;(12):15-17.

17 Hahn SZA, Needham IEA. Patient and visitor violence in general hospitals: a systematic review of the literature. *Aggression Violent Behav* 2008;13:431–41.

18 Chen ZH, Wang SY. Psychiatric hospital and general hospital workplace violence study comparison. *Chinese Journal of Public Health* 2004;20:1316–1317.

19 ILO/ICN/WHO/PSI. Framework Guidelines for Addressing Workplace Violence in the Health Sector.Geneva: ILO/ICN/WHO/PSI Joint Programme on Workplace Violence in the Health Sector

2002:4-132.

20 Laschinger HK, Grau AL. The influence of personal dispositional factors and organizational resources on workplace violence, burnout, and health outcomes in new graduate nurses: A cross-sectional study. *Int J Nurs Stud* 2012;49:282–291.

21 Kitaneh M, Hamdan M. Workplace violence against physicians and nurses in Palestinian public hospitals: A cross-sectional study. *BMC Health Serv Res* 2012;12:469.

22 Carter R. High risk of violence against nurses. *Nurs Manag* 2000;6:5.

23 Merecz D, Rymaszewska J, Moscicka A, *et al*. Violence at the workplace-a questionnaire survey of nurses. *Eur Psychiatry* 2006;21:442–450.

24 Ferns T. Under-reporting of violent incidents against nursing staff. *Nurs Stand* 2006;20(40) :41–45.

25 Chen ZH, Wang SY, Jing CX. Investigation on workplace violence phenomenon of two hospitals in Guangzhou. *Chinese Journal of Preventive Medicine* 2003;37:358–360.

26 Zampieron A, Galeazzo M, Turra S, et al. Perceived aggression towards nurses: study in two Italian health institutions. *J Clin Nurs* 2010;19:2329–41.

27 Gerberich SG, Church TR, McGovern PM, *et al.* An epidemiological study of the magnitude and consequences of work related violence: the Minnesota Nurses' Study. *Occup Environ Med* 2004;61: 495–503.

28 Franz S, Zeh A, Schablon A, *et al*. Aggression and violence against health care workers in Germany–a cross sectional retrospective survey. *BMC Health Serv Res* 2010;10:51.

29 Hahn S, Muller M, Needham I, *et al.* Factors associated with patient and visitor violence

For peer review only

experienced by nurses in general hospitals in Switzerland: a cross-sectional survey. *J Clin Nurs* 2010; 19:3535–46.

30 Hesketh KL, Duncan SM, Estabrooks CA, *et al*. Workplace violence in Alberta and British Columbia hospitals. *Health Policy* 2003;63:311-21.

31 Findorff MJ, McGovern PM, Wall M, *et al*. Risk factors for work related Violence in a health care organization. *Inj Prev* 2004;10(5):296–302.

32 Magin PJ, May J, McElduff P. Occupational violence in general practice: a whole-of-practice problem. Results of a cross-sectional study. *Australian Health Review* 2011;35(1):75–80.

33 Hegney D, Plank A, Parker V. Workplace violence in nursing in Queensland, Australia: A self-reported study. *Int J Nurs Pract* 2003;9:261–268.

34 Kwok RP, Law YK, Li KE, *et al*. Prevalence of workplace violence against nurses in Hong Kong. *Hong Kong Med J* 2006;12:6–9.

35 Hegney D, Plank A, Parker V. Workplace violence in nursing in Queensland, Australia: A self-reported study. *Int J Nurs Pract* 2003;9:261–268.

36 Whittington R, Wykes T. An evaluation of staff training in psychological techniques for the management of patient aggression. *J Clin Nurs* 1996;5(4):257–261.

37 Sun T, Wang S, Wang N, *et al*. Epidemiological characteristics and variance analysis of workplace violence in public hospitals. *Practical Preventive Medicine* 2014;21(4):478-480.

38 Hodgson MJ, Reed R, Craig T, *et al*. Violence in healthcare facilities:lessons from the Veterans Health Administration. *J Occup Environ Med* 2004;46(11):1158–1165.

For peer review only

# A survey on patient and visitor violence experienced by nurses in general hospitals of China

Dear nurses:

Hello! In recent years, nurses suffered suffered more and more serious violence. This study is based on the perspective of the nurses, understanding the nurses in the violence in the process of experience, violence after the occurrence of physical and mental injury to nurses and the views of the hospital violence. In order to safeguard the legitimate rights and interests of nurses , maintain the normal order of the hospital, we will conduct a "hospital workplace Violence "situation investigation.The survey related to any of your personal information, we will be for you to keep confidential, please rest assured!

**Note: Please tick the appropriate box, some problems can select multiple items, part of the problem, please fill in the "_____".**

Name_____________ Contact information_____________

**Part1：Please indicate your information**

Q1.Gender: Male□ Female□

Q2.Age: ≤30years□ 31-40years□ 41-50years□ 51-60years□ ＞60years□

Q3.Level of education: ＜Bachelor□ Bachelor□ ≥Master□

Q4.Marital status: Married□ Single□ Other□

Q5.Professional title: Junior□ Intermediate□ Senior□

Q6.Employment form: formal employee□ temporary employee□

Q7.Monthly income(RMB):
＜3000□ 3000-5000□ 5000-10000□ ＞10000□

Q8.Department: Emergency□ Inter Medical□ Surgical□ Oncology□ Orthopedic□ Gynaecology□ Pediatrics□ Stomatology□ Ophthalmic□ ENT□ Medical Imaging□ Other□

Q9.Years of Working: ＜1years□ 1-4years□ 5-10years□ 11-20years□ ＞20years□

Q10.Treatment Time: 0-2hours□ 2-4hours□ 4-6hours□ 6-8hours□ ＞8hours□

Q11.The contact time with the patient: 0-2□ 2-4□ 4-6□ 6-8□

**Part2：In the past 12 months, have you ever encountered the following in the hospital?**

| Types of violence | | | | |
|---|---|---|---|---|
| Psychological violence | No | 1 times | 2-3 times | ≥4 times |
| 1. Scold, abuse, abusive, demeaning or other detrimental to the dignity of the individual words. (face to face, telephone, letter or leaflets, micro-network, etc). | | | | |

For peer review only

| | | | | |
|---|---|---|---|---|
| 2.Threats: threats related to personal and property safety, threatening complaints, trailing tracking and so on. | | | | |
| 3.Sexual harassment or sexual provocation, including language, movement or exposure of sexual organs. | | | | |
| Physical violence(To physical contact or something to attack, including playing, kick, push, bite, throw objects and so on). | | | | |
| 4.Causing mild injury, such as pain, abrasions, scratches, or ecchymosis. | | | | |
| 5.Resulting in significant injury, such as wounds, fractures, visceral or head injury | | | | |
| 6.Causing serious consequences, such as dysfunction or permanent disability | | | | |
| 7.Sexual assault, such as by the opposite sex to pull, hold, kiss or touch the sensitive parts. | | | | |
| 8.Rape or attempted rape | | | | |

**Δ If you do not have the above experience, please skip to the fourth part of the beginning to continue to answer.**

**Part3: Please describe the most profound event in your memory over the last 12 months.**

1.Location of the incident:

Outpatient department□ Ward□ Doctor's Office□

Nursing station/office□ Treatment room□ Other□

2.Time of occurrence：Day shift□ Night shift□ After work□

3.When the event occurs, you are：

Alone□ There are other colleagues present□

4.The perpetrators are (can choose more than one):

Patient□ Family members□ Visitors□ Other□

5.Perpetrators of gender (If more than one person, can choose more than one): Male□ Female□

6.Perpetrators of age (If more than one person, can choose more than one):

≤20years□ 21-30years□ 31-40years□

41-50years□ 51-60years□ ＞60years□

7. When this event occurs, what do you think the following circumstances compared with the perpetrators? (you can choose more than one)

Patient disability or death□ Drinking and drug abuse□

Patients with dysfunction or complications□ Mental disorders□

Long waiting time for treatment□ Low education level□

Unreasonable demands are rejected□

Not satisfied with treatment effect□

Not satisfied with the service attitude of medical staff□

For peer review only

The hospital service process is cumbersome□
Medical expenses are expensive□
Want to seek economic compensation□ Other_____________
8.How do you choose to respond to such incidents? (you can choose more than one)
Tolerance and avoidance□ Patience and understanding□
Give tit for tat（such as sparring、insults）□
After the first reasonable counterattack□ Ask a colleague for help□
Ask a managers or security for help□ Call the police□
Ask a other patients or family member for help□ Other□
9.The impact of the incident on your psychological and behavioral? (you can choose more than one)
No effect□ Unable to concentrate□ Aggrievement□
Angry□ Anxious□ Insomnia□ Depressed□
Work enthusiasm decline□ Work efficiency decline□
Hate and fear of patients and their families□
Interpersonal communication barriers□ Have turnover intention□
Have suicidal thoughts□ Scene repetition□ Other□
10.Because of this event to see a doctor？
Psychological counseling □Routine treatment□ No□

**Part4: Perceptions and attitudes of workers towards workplace violence**

1.Have you heard of the term " hospital workplace violence "?
Yes□ No□
2.Do you think "hospital workplace violence" is inevitable in medical work? Yes□ No□
3.Do you think the "hospital workplace violence" is not worth the fuss?
Yes□ No□
4.What do you think should be included the content of the prevention of hospital workplace violence in the medical post training?
Yes□ No□
5.Are you willing to take part in the training of the hospital's workplace violence Prevention related knowledge? Yes□ No□
6.Do you think it will benefit you to take part in the prevention of hospital workplace violence? Yes□ No□
7.Do you think the hospital is designed to deal with the "hospital workplace violence " institutions or organizations to prevent hospital violence is effective? Yes□ No□
8.Which of the following measures can be taken to prevent or reduce the occurrence of hospital workplace violence?
(you can choose more than one)
(1)Government for hospital violence legislation□
(2)Participate in medical liability insurance□
(3)Through third-party mediation□ (4)Outpatient and ward installation camera□
(5)Reduce the workload of medical services□
(6)Improve the environment for medical treatment□

For peer review only

(7)Optimize service flow□ (8)Improve service attitude□
(9)Improve interpersonal communication skills□
(10)To carry out targeted training□
(11)Strengthen the ability to deal with violence□
(12)To improve the service level and treatment ability□
(13)Increase security personnel□
(14)Public security institutions stationed in hospital□
(15) Media correct guidance of public opinion□
(16) Advocating social respect for medical workers□
(17) Other__________________

**Part5: Hospital attitudes to workplace violence and intervention strategies.**

1.Does your hospital have staff training related to hospital workplace violence prevention? Yes□ No□

2.What are the measures taken by your hospital to prevent hospital workplace violence?
(1)Regular security patrols throughout the day□
(2)Installation of cameras in Outpatient and ward□
(3)Installation of alarm equipment in Outpatient and ward□
(4)Setting a fixed waiting area seat□ (5) third-party mediation□
(6)The establishment of medical liability insurance□
(7)Public security departments stationed□ (8)Other______________

3.Does your facility encourage employees to report in a timely manner after being exposed to hospital workplace violence? Yes□ No□

4.Does your hospital have specialized departments to handle hospital workplace violence for employees? Yes□ No□

5.Do you think it is reasonable for your hospital to handle hospital workplace violence in the past? Yes□ No□

6.Do you think it is timely for your hospital to handle hospital workplace violence in the past? Yes□ No□

7.The attitude of the hospital to employees who suffered from workplace violence:
(1)Actively protect the interests of employee□ (2)Basing on facts and fair dealing□
(3)Neither stand nor actively□ (4)Punishing employees□

8.The degree of attention of hospital after doctors and nurses exposed to violence:
(1)Paying more attention to the doctor□ (2)Paying more attention to the nurse□
(3)Consistent the degree of attention□

**Thank you very much for your assistance, please check the above items are not missing fill !**

For peer review only

!

1. **the National Natural Science Foundation of China, grant number71473063**

# 国家自然科学基金委员会
## 项 目 批 准 通 知

国科金计项〔2014〕44号

### 关于批准资助2014年度第二批项目的通知

哈尔滨医科大学（单号：2014-44-0330）：

根据《国家自然科学基金条例》有关规定和专家评审意见，国家自然科学基金委员会（以下简称自然科学基金委）决定批准资助你单位2014年度（第2批）国家自然科学基金项目 125 项，金额 5902.0 万元。其中，面上项目 51 项，重点项目 2 项，青年科学基金项目 72 项，上述资助项目清单详见附件。

自评审结果通告发布之日起25日内，项目负责人须按要求填写与提交《国家自然科学基金资助项目计划书》（以下简称计划书）电子版。2014年9月11日16点前，依托单位将审核后的计划书电子版通过科学基金网络信息系统（https://isis.nsfc.gov.cn）提交至自然科学基金委。自然科学基金委同期对计划书电子版进行审核。审核通过的，项目负责人可打印计划书纸质版（建议双面打印）；审核未通过的，退回至项目负责人修改，依托单位须在2014年9月18日16点前，将修改后的计划书电子版及时审核并再次提交至自然科学基金委。2014年9月26日16点前，依托单位须将自然科学基金委审核通过后的计划书纸质版（一式两份，应保证与电子版一致）加盖单位公章，报送至自然科学基金委项目材料接收工作组。采用邮寄方式的，请在截止日前（以发信

邮戳日期为准）以快递方式邮寄，并在信封左下角注明“计划书”。请勿使用包裹，以免延误报送。报送计划书材料时，还应包括本单位报送计划书的公函和计划书清单。材料不完整不予接收。

如在规定期限内未提交电子与纸质计划书且未说明理由的，视为自动放弃接受资助。

邮寄地址：北京市海淀区双清路83号项目材料接收工作组

邮编：100085

联系电话：010-62328591

附件：2014年度国家自然科学基金资助项目清单

国家自然科学基金委员会

2014年8月25日

# 2014年国家自然科学基金资助项目清单（哈尔滨医科大学）

单号：2014-44-0330 资助金额单位：万元

| 序号 | 项目批准号 | 负责人 | 申请代码 | 项目名称 | 资助金额 | 起止日期 | 资助类别/亚类说明/附注说明 |
|---|---|---|---|---|---|---|---|
| 12 | 61403112 | 刘洪波 | F030406 | 基于高通量组学数据的干细胞多能性相关DNA甲基化调控元件识别及其功能分析 | 25 | 2015.01.01-2017.12.31 | 青年科学基金项目 |
| 13 | 61473106 | 李霞 | F030407 | 癌症非编码miRNA-lncRNA协同调控网络重构与功能特性研究 | 82 | 2015.01.01-2018.12.31 | 面上项目/常规面上项目 |
| 14 | 71403071 | 孙涛 | G0308 | 基于生态理论视角下区域医疗联合体生态系统健康评价建模与实证研究 | 23 | 2015.01.01-2017.12.31 | 青年科学基金项目 |
| 15 | 71403072 | 王月枫 | G0308 | 社区高血压患者急症风险预测模型构建及预见性干预与救护管理体系的研究 | 20 | 2015.01.01-2017.12.31 | 青年科学基金项目 |
| 16 | 71403073 | 李叶 | G0308 | 基于CHE方法学比较分析的医保制度经济保护能力影响因素研究 | 23 | 2015.01.01-2017.12.31 | 青年科学基金项目 |
| 17 | 71403074 | 尹慧 | G0308 | 基于结构方程的社区心血管疾病的社会决定因素模型构建及分层干预效果评价 | 20 | 2015.01.01-2017.12.31 | 青年科学基金项目 |
| 18 | 71473063 | 樊立华 | G0308 | 医护人员遭受医院场所暴力PTSD及对职场行为影响机制研究 | 63 | 2015.01.01-2018.12.31 | 面上项目/常规面上项目 |
| 19 | 71473064 | 焦明丽 | G0308 | 基于DEMATEL 法与"破窗理论"的医院暴力影响因素及对策研究 | 60 | 2015.01.01-2018.12.31 | 面上项目/常规面上项目 |
| 20 | 71473065 | 宁宁 | G0308 | 面向公共卫生灾害的城市社区抗逆力理论、方法及策略研究 | 63 | 2015.01.01-2018.12.31 | 面上项目/常规面上项目 |
| 21 | 81400097 | 高春艳 | H0811 | 血管内皮细胞清除活化和凋亡的血小板调控血栓形成的作用与机制 | 23 | 2015.01.01-2017.12.31 | 青年科学基金项目 |
| 22 | 81400115 | 付玥玥 | H0812 | APL分化综合征miRNA标记筛选及功能分析 | 23 | 2015.01.01-2017.12.31 | 青年科学基金项目 |

第2页 / 共12页

## 2. Ethical Approval:

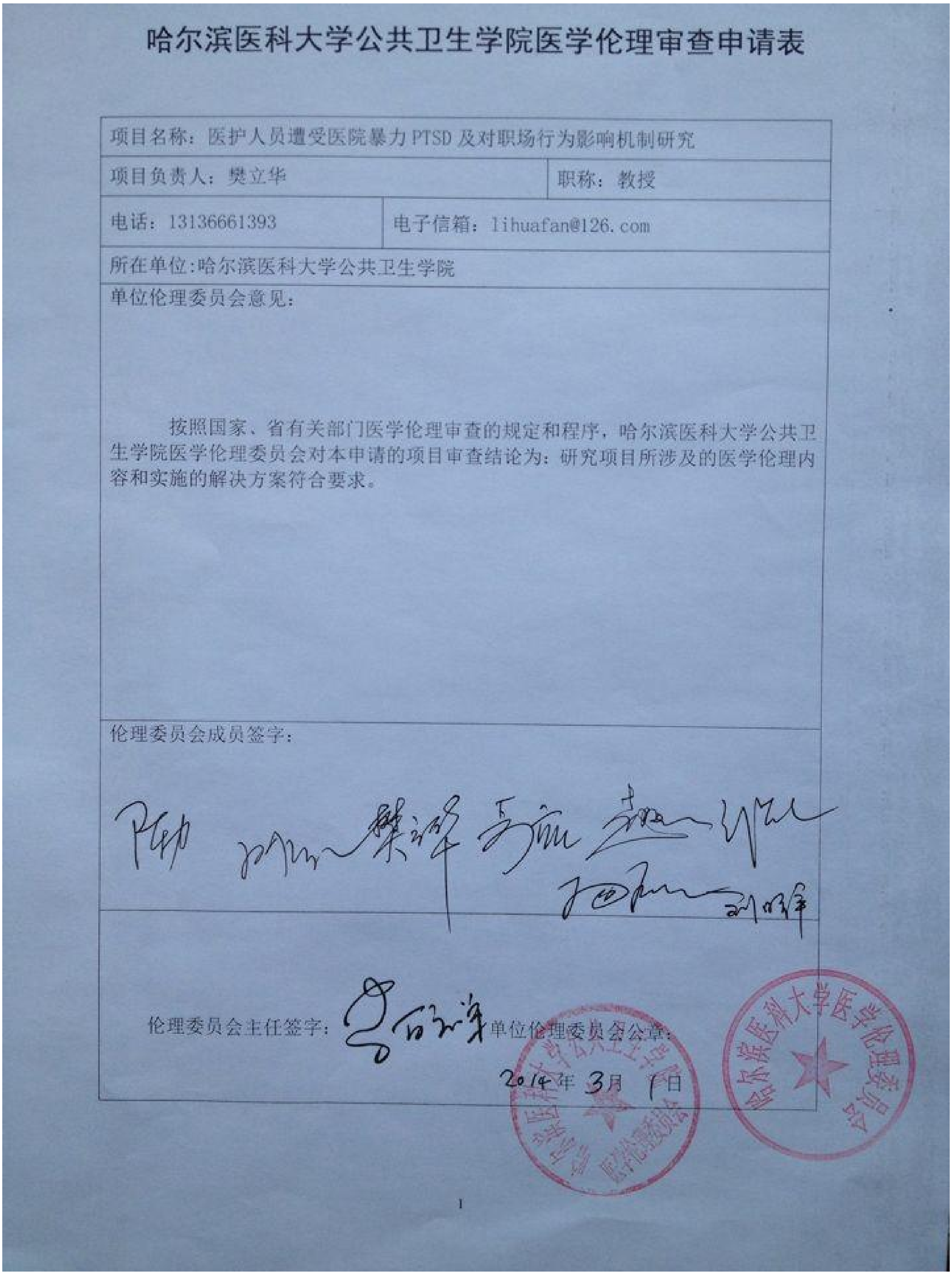

# 哈尔滨医科大学公共卫生学院医学伦理审查申请表

| | |
|---|---|
| 项目名称：医护人员遭受医院暴力 PTSD 及对职场行为影响机制研究 | |
| 项目负责人：樊立华 | 职称：教授 |
| 电话：13136661393 | 电子信箱：lihuafan@126.com |
| 所在单位:哈尔滨医科大学公共卫生学院 | |

单位伦理委员会意见：

按照国家、省有关部门医学伦理审查的规定和程序，哈尔滨医科大学公共卫生学院医学伦理委员会对本申请的项目审查结论为：研究项目所涉及的医学伦理内容和实施的解决方案符合要求。

伦理委员会成员签字：

伦理委员会主任签字： 单位伦理委员会公章：

2014年 3月 1日

1

Checklist of ***cross-sectional studies***

| | Page number, line number |
|---|---|
| **Title** | 1, 4-6 |
| **Abstract** | 2, 4-55 |
| **Strengths and limitations of this study** | 3, 4-24 |
| **Introduction** | |
| Background | 4, 4-57; 5, 4-19 |
| Objectives | 5, 21-29 |
| **Methods** | |
| Study design | 5, 37-52 |
| Setting | 5, 52-57; 6, 4-19 |
| Participants | 6, 21-27 |
| Questionnaire | 6, 47-57; 7, 4-17 |
| Data measurement | 7, 21-47 |
| Study size | 6, 39-42 |
| Quantitative variables | 7, 29-42 |
| Ethical considerations | 7, 52-57; 8,4-6 |
| **Results** | |
| Participants | 8, 14-21 |
| Descriptive data | 8, 22-58; 9, 2-35 |
| Outcome data | 9, 39-58 |
| Main results | 10; 11; 12;14 |
| Other analyses | 10, 16-57; 11, 3-26 |
| **Discussion** | |
| Key results | 13,16-50; 15, 4-54 |
| Interpretation | 16, 29-57; 17, 4-29 |
| Discuss the external validity of the study results | 16, 3-27 |
| **Conclusions** | 17, 54-57; 18, 4-11 |
| **Other information** | |
| Acknowledgements | 18, 16-24 |
| Contributors | 18, 27-32 |
| Funding | 18, 34-36 |
| Competing interests | 18, 42 |
| Ethical approval | 18, 47-52 |
| **References** | 19; 20; 21; 22; 23 |

For peer review only

BMJ Open

# A cross-sectional study on the prevalence and associated risk factors for workplace violence against Chinese nurses



| | |
|---|---|
| Journal: | *BMJ Open* |
| Manuscript ID | bmjopen-2016-013105.R2 |
| Article Type: | Research |
| Date Submitted by the Author: | 03-Feb-2017 |





| | |
|---|---|
| Complete List of Authors: | Shi, Lei; School of Public Health, Harbin Medical University, Department of Health Management<br>Zhang, Danyang; The First Affiliated Hospital of Harbin Medical University, Operating Section<br>Zhou, Chenyu; School of Public Health, Harbin Medical University, Department of Health Management<br>Yang, Libin; School of Public Health, Harbin Medical University, Department of Medical Education<br>Sun, Tao; School of Public Health, Harbin Medical University, Department of Health Management<br>Hao, Tianjun; The Second Affiliated Hospital of Harbin Medical University, Department of Scientific Research<br>Peng, Xiangwen ; The First Affiliated Hospital of Harbin Medical University, Harbin, Operating Section<br>Gao, Lei; School of Public Health, Harbin Medical University, Department of Health Management<br>Liu, Wenhui; School of Public Health, Harbin Medical University, Department of Medical Education<br>Mu, Yi; Beijing Children's Hospital, Capital Medical University, Department of Customer Service<br>Han, Yuzhen; The Fourth Affiliated Hospital of Harbin Medical University, Department of Medical Disputes<br>Fan, Lihua; School of Public Health, Harbin Medical University, Department of Health Management |





| | |
|---|---|
| <b>Primary Subject Heading</b>: | Health services research |
| Secondary Subject Heading: | Health policy, Medical management, Nursing |
| Keywords: | workplace violence (WPV), nurses, general and public hospitals, risk factors |

SCHOLARONE™ Manuscripts

# A cross-sectional study on the prevalence and associated risk factors for workplace violence against Chinese nurses

Lei Shi, [1] Danyang Zhang,[2] Chenyu Zhou,[1] Libin Yang,[3] Tao Sun,[1] Tianjun Hao,[4] Xiangwen Peng,[2] Lei Gao,[1] Wenhui Liu,[3] Yi Mu,[5] Yuzhen Han,[6] Lihua Fan[1]

**Correspondence to:** Lihua Fan, School of Public Health, Harbin Medical University, 157 Baojian Road, Nangang District, Harbin 150081, China; E-mail: lihuafan@126.com

[1]Department of Health Management, School of Public Health, Harbin Medical University, Harbin, China

[2] Operating Section, The First Affiliated Hospital of Harbin Medical University, Harbin, China

[3]Department of Medical Education, School of Public Health, Harbin Medical University, Harbin, China

[4]Department of Scientific Research, The Second Affiliated Hospital of Harbin Medical University, Harbin, China

[5]Department of Customer Service, Beijing Children's Hospital, Capital Medical University, Beijing, China

[6]Department of Medical Disputes, The Fourth Affiliated Hospital of Harbin Medical University, Harbin, China

For peer review only

**Abstract**

**Objectives:** The purpose of the present study was to explore the characteristics of workplace violence that Chinese nurses at tertiary and county-level hospitals encountered in the 12 months from December 2014 to January 2016, to identify and analyze risk factors for workplace violence, and to establish the basis for future preventive strategies.

**Design:** A cross-sectional study.

**Setting:** A total of 44 tertiary hospitals and 90 county-level hospitals in 16 provinces (municipalities or autonomous regions) in China.

**Methods:** We employed stratified random sampling to collect data from December 2014 to January 2016. We distributed 21,360 questionnaires, and 15,970 participants provided valid data (effective response rate = 74.77%). We conducted binary logistic regression analyses on the risk factors for workplace violence among the nurses in our sample and analyzed the reasons for aggression.

**Results:** The prevalence of workplace violence was 65.8%; of this, 64.9% was verbal violence, and physical violence and sexual harassment accounted for 11.8% and 3.9%, respectively. Frequent workplace violence occurred primarily in emergency and pediatric departments. Respondents reported that patients' relatives were the main perpetrators in tertiary and county-level hospitals. Logistic regression analysis showed that respondents' age, department, years of experience, and direct contact with patients were common risk factors at different levels of hospitals.

**Conclusions**: Workplace violence is frequent in China's tertiary and county-level hospitals. The frequent occurrence of WPV in emergency and pediatric departments is also remarkable. It is necessary to cope with workplace violence by developing effective control strategies at individual, hospital, and national levels.

For peer review only

**Strengths and limitations of this study**

- We used a large nurse-based sample in our study.
- Sample selection was reasonable and representative, and was distributed in the eastern, middle, and western regions of China.
- Our study compared differences between nurses who had experienced workplace violence in comprehensive public tertiary and county-level hospitals.
- The study provides the basis for establishing operational guidelines for preventing workplace violence in China.
- The retrospective approach to self-reported workplace violence used by respondents may cause recall bias.

For peer review only

**BACKGROUND**

Workplace violence (WPV) towards health service professionals is recognized as a global public health issue, and it has attracted worldwide attention.[1-4] Previous studies have suggested that health professionals have a higher risk of experiencing WPV than any other professionals.[2-3] Further, the incidence rate of WPV differs among nurses in various countries; for instance, the incidence was 76.0% in Greece, 82% in Pakistan, and 67% in Italy.[5-6]

Several substantial studies have suggested that nurses have a high risk of experiencing WPV.[6-10] During the past 12 months, the incidence rate of physical violence for nurses in Ethiopia,[7] South Korea,[8] Jordan,[9] Germany,[10] and Iran[3] ranged from 18.22% to 56.0%, the verbal abuse rate being from 63.8% to 89.58% and the sexual harassment rate from 4.7% to 19.7%. WPV occurs primarily in the emergency wards and psychiatric departments of hospitals.[11-12] Research into the experience of nurses in these departments in the US,[13] Switzerland,[14] and Jordan[15] has demonstrated that they experience a higher incidence of WPV than do nurses in other departments.

In China, WPV in hospitals has increased gradually over the past few decades. According to the report from the Chinese Hospital Association, the proportion of hospitals experiencing WPV rose from 90% in 2008 to 96% in 2012 and the prevalence of sexual harassment has increased year by year.[16]

Most previous studies of WPV against nurses in China have been conducted in the provinces, and the samples are not sufficiently representative: they do not present an accurate picture of the incidence of WPV against nurses in Chinese general hospitals.[17-19] In this study, WPV is divided into physical violence and psychological violence, which includes verbal violence and sexual harassment in accordance with the definition of WPV in hospitals used by the World Health Organization (WHO), the International Labour Office (ILO), and the specific situation in China.

For peer review only

Although violent incidents in hospitals directly harm the healthcare workers and hospitals, the ultimate victims are the patients.[20] WPV affects the normal functioning and reputation of hospitals and threatens the personal safety of healthcare workers and patients.[21] Moreover, violent incidents have a negative impact on the psychological welfare of the healthcare workers,[22] who do not want their children to be engaged in healthcare.[23]

The purpose of the present study was to explore the distribution and characteristics of WPV experienced by Chinese nurses at tertiary and county-level hospitals in the 12 months from December 2014 to January 2016, to identify and analyze the risk factors for WPV, thus providing a basis for future preventive strategies.

## METHODS

### Sample and procedure

A cross-sectional survey was designed based on the geographical location and level of economic development in the eastern (Beijing, Tianjin, Hebei, Shandong, Guangdong, Liaoning), middle (Shanxi, Henan, Anhui, Hunan, Heilongjiang), and western regions of China (Ningxia, Shannxi, Gansu, Sichuan, Chongqing). In 2015, there were 3,069 general and public hospitals in China and approximately 920,700 registered nurses in public tertiary and county-level hospitals. We selected a sample of 21,360, approximately 2.30% of all nurses. In order to select the same proportion of the workforce from each department, we sought to recruit 120 nurses from the Departments of Internal Medicine and Surgery, 80 nurses from the Departments of Emergency, Neurology, Obstetrics and Gynecology, and Pediatrics, and 40 nurses from the Departments of Stomatology, Ophthalmology, and Ear, Nose and Throat in each tertiary hospital. We distributed 10,560 questionnaires to 44 tertiary hospitals. We sought to recruit

For peer review only

nurses at county-level hospitals in the same proportions: 60 from the Departments of Internal Medicine and Surgery, 40 from the Departments of Emergency, Neurology, Obstetrics and Gynecology, and Pediatrics, and 20 from the Departments of Stomatology, Ophthalmology, and Ear, Nose and Throat at each county-level hospital. We distributed 10,800 questionnaires to the 90 county-level hospitals.

All 134 hospitals agreed to participate in the study. The inclusion criteria for participants were 1) voluntary participation by nurses engaged in clinical work who 2) had at least one year of professional experience in hospitals.

The survey was conducted from December 2014 to January 2016. We obtained permission from the managers and human resources departments of the hospitals. The researchers used a stratified random sampling method to collect the data. Each participant had two days to complete the self-administered questionnaire. Collected data were kept confidential and used only for academic research. We distributed 21,360 questionnaires and received 15,970 valid questionnaires (effective response rate = 74.77%).

**QUESTIONNAIRE**

We developed the questionnaire based on three documents. First, we used the revised Survey of Violence Experienced by Staff (SOVES-G-R) developed by Hahn and colleagues to formulate the questionnaire.[24] The revised SOVES-G-R was used to measure perpetrators' characteristics and the way to deal with violence for nurses. Next, we used items from the Chinese version of the Workplace Violence Scale (WVS) in the literature (the frequency of violence, different levels of the severity of physical violence, and hospital attitudes to WPV and intervention strategies) to develop the questionnaire.[25] Chen and Wang's study incorporated the levels of the severity of physical violence,

For peer review only

and hospital attitudes to WPV and intervention strategies. In addition, we used the questionnaires from the ILO, ICN (International Council of Nurses), WHO, and PSI (Public Services International) joint program for measuring WPV (e.g., types of WPV, time and place the violence occurred, and perceptions and attitudes of nurses towards WPV).[26] We pre-tested the questionnaire with 367 nurses from three public tertiary and two public county-level hospitals in Heilongjiang Province and revised the questionnaire post-test. Further, we invited 18 healthcare-related experts to assess the accuracy, comprehensiveness, and sensitivity of the items in the questionnaire. In order to ensure the reliability of the revised questionnaire according to experts' opinions, we selected 835 nurses from five public tertiary and four public county-level hospitals in Heilongjiang Province to measure the reliability of the questionnaire again. We measured the questionnaire's reliability twice using SPSS19.0; Cronbach's alpha coefficient was 0.84 and 0.86, respectively.

## DATA ANALYSIS

EpiData version 3.1 was used to establish the study's database. To ensure accuracy, the data were checked by trained personnel after all surveys were completed and entered. We used IBM SPSS19.0 and Excel for statistical analysis of the relevant quantitative data. Univariate analysis and chi-square tests ($\chi^2$) were used to analyze the relationship between the demographic characteristics of respondents and the incidence rate of WPV. We employed logistic regression analysis to test the relationship between nurses experiencing hospital WPV as the dependent variable, and the variables with statistical and clinical practice significance as the covariates. We conducted binary logistic regression analyses on the risk factors for WPV among the nurses in our sample. Odds ratios (OR) and 95% confidence intervals (CI) were calculated; $\alpha = 0.05$ was the test and $P < 0.05$ was considered statistically

For peer review only

significant.

## ETHICAL CONSIDERATIONS

Ethical approval to undertake this study was granted by the Research Ethics Committee of Harbin Medical University in March 2014. We obtained consent from each hospital involved in the research processes. All participants gave informed consent to the researchers or to their head nurses before the survey, and participants' personal information was kept confidential.

## RESULTS

### Participants' demographic characteristics

We received 17,865 responses, and 15,970 respondents met our inclusion criteria. Most had completed undergraduate studies; most respondents were less than 30 years old; and there was a large difference in the proportion of gender (Table 1).

**Table 1** Participants' demographic characteristics (N=15970)

| Characteristics | Tertiary hospitals (n=9142) | | County-level hospitals (n=6828) | | Total (n=15970) | |
|---|---|---|---|---|---|---|
| | n | % | n | % | n | % |
| Gender | | | | | | |
| Male | 234 | 2.6 | 143 | 2.1 | 377 | 2.4 |
| Female | 8908 | 97.4 | 6685 | 97.9 | 15593 | 97.6 |
| Age group (years) | | | | | | |
| ≤30 | 5131 | 56.1 | 4033 | 59.1 | 9164 | 57.4 |
| 31–50 | 3785 | 41.4 | 2611 | 38.2 | 6396 | 40.0 |
| ≥51 | 226 | 2.5 | 184 | 2.7 | 410 | 2.6 |
| Education status | | | | | | |
| Below undergraduate | 3129 | 34.2 | 4161 | 60.9 | 7290 | 45.6 |
| Undergraduate | 5910 | 64.6 | 2663 | 39.0 | 8573 | 53.7 |
| Master's or above | 103 | 1.1 | 4 | 0.1 | 107 | 0.7 |
| Marital status | | | | | | |
| Married | 5678 | 62.1 | 4293 | 62.9 | 9971 | 62.4 |
| Unmarried | 3342 | 36.6 | 2415 | 35.4 | 5757 | 36.1 |

For peer review only

| | | | | | | |
|---|---|---|---|---|---|---|
| Divorced or Widowed | 122 | 1.3 | 120 | 1.7 | 242 | 1.5 |
| Professional title | | | | | | |
| Junior | 6249 | 68.4 | 4845 | 71.0 | 11094 | 69.5 |
| Intermediate | 2242 | 24.5 | 1587 | 23.2 | 3829 | 24.0 |
| Senior | 651 | 7.1 | 396 | 5.8 | 1047 | 6.5 |
| Employment form | | | | | | |
| Regular staff | 5077 | 55.5 | 3339 | 48.9 | 8416 | 52.7 |
| Temporary employee | 4065 | 44.5 | 3489 | 51.1 | 7554 | 47.3 |
| Average monthly income (RMB) | | | | | | |
| ≤ 3000 | 3747 | 41.0 | 4916 | 72.0 | 8663 | 54.3 |
| 3000–5000 | 4495 | 49.2 | 1867 | 27.3 | 6362 | 39.8 |
| 5000–10000 | 872 | 9.5 | 42 | 0.6 | 914 | 5.7 |
| > 10000 | 28 | 0.3 | 3 | 0.1 | 31 | 0.2 |
| Department | | | | | | |
| Emergency | 514 | 5.6 | 522 | 7.6 | 1036 | 6.5 |
| Internal medicine | 2850 | 31.2 | 1962 | 28.7 | 4812 | 30.1 |
| Surgery | 1903 | 20.8 | 1386 | 20.3 | 3289 | 20.6 |
| Gynaecology and Obstetrics | 426 | 4.7 | 610 | 8.9 | 1036 | 6.5 |
| Paediatrics | 516 | 5.6 | 627 | 9.2 | 1143 | 7.2 |
| Others | 2933 | 32.1 | 1721 | 25.3 | 4654 | 29.1 |
| Years of experience | | | | | | |
| 1–4 | 3511 | 38.4 | 3124 | 45.7 | 6635 | 41.5 |
| 5–10 | 2803 | 30.7 | 1597 | 23.4 | 4400 | 27.6 |
| 11–20 | 1534 | 16.8 | 1099 | 16.1 | 2633 | 16.5 |
| ≥ 21 | 1294 | 14.1 | 1008 | 14.8 | 2302 | 14.4 |
| Working time | | | | | | |
| 0–2 h | 164 | 1.8 | 52 | 0.8 | 216 | 1.3 |
| 2–4 h | 252 | 2.8 | 120 | 1.8 | 372 | 2.3 |
| 4–6 h | 264 | 2.9 | 113 | 1.6 | 377 | 2.4 |
| 6–8 h | 3713 | 40.6 | 3087 | 45.2 | 6800 | 42.6 |
| > 8 h | 4749 | 51.9 | 3456 | 50.6 | 8205 | 51.4 |
| Direct contact with patients | | | | | | |
| 0–2 h | 180 | 2.0 | 106 | 1.6 | 286 | 1.8 |
| 2–4 h | 237 | 2.6 | 251 | 3.7 | 488 | 3.0 |
| 4–6 h | 747 | 8.2 | 575 | 8.4 | 1322 | 8.3 |
| 6–8 h | 7978 | 87.2 | 5896 | 86.3 | 13874 | 86.9 |

**Incidence of exposure to WPV during the past 12 months**

In the 12-month survey period, 10,502 nurses experienced WPV. Total prevalence was 65.8%, of which verbal violence accounted for 64.9% (10369/15970), physical violence for 11.8% (1892/15970), and

For peer review only

sexual harassment for 3.9% (630/15970). The incidence rate of WPV in the different types of hospitals is shown in Table 2.

**Table 2** Incidence (%) of exposure to WPV

| Type | Tertiary hospitals | | | | | | County-level hospitals | | | | | |
|---|---|---|---|---|---|---|---|---|---|---|---|---|
| | Physical violence | | Verbal violence | | Sexual harassment | | Physical violence | | Verbal violence | | Sexual harassment | |
| | n | % | n | % | n | % | n | % | n | % | n | % |
| | 1047 | 11.5 | 5875 | 64.3 | 402 | 4.4 | 845 | 12.4 | 4494 | 65.8 | 228 | 3.3 |

**Prevalence of WPV for different demographic variables**

Nurses with frequent contact with patients were more likely to experience WPV in both tertiary and county-level hospitals (Table 3). There was a significant difference in the incidence rate of WPV among different professional titles for nurses in the tertiary hospitals ($\chi^2 = 6.6$, $P < 0.05$). In contrast, there was no significant difference in county-level hospitals ($\chi^2 = 1.5$, $P > 0.05$).

**Table 3** Characteristics and frequency distributions for WPV

| Characteristics | Tertiary hospitals (n=9142) | | | | County-level hospitals (n=6828) | | | |
|---|---|---|---|---|---|---|---|---|
| | n | % | $\chi^2$ | *P* | n | % | $\chi^2$ | *P* |
| Gender | | | | | | | | |
| Male | 143 | 61.1 | 1.8 | 0.181 | 85 | 59.4 | 3.2 | 0.072 |
| Female | 5820 | 65.3 | | | 4454 | 66.6 | | |
| Age | | | | | | | | |
| ≤ 30 | 3283 | 64.0 | 15.4 | <0.001 | 2679 | 66.4 | 15.6 | <0.001 |
| 31–50 | 2548 | 67.3 | | | 1762 | 67.5 | | |
| ≥ 51 | 132 | 58.4 | | | 98 | 53.3 | | |
| Education | | | | | | | | |
| Below undergraduate | 1958 | 62.6 | 15.7 | <0.001 | 2707 | 65.1 | 9.7 | 0.008 |
| Undergraduate | 3941 | 66.7 | | | 1829 | 68.7 | | |
| Master's or above | 64 | 62.1 | | | 3 | 75.0 | | |
| Marital status | | | | | | | | |
| Married | 3813 | 67.2 | 27.8 | <0.001 | 2884 | 67.2 | 3.3 | 0.192 |
| Unmarried | 2065 | 61.8 | | | 1581 | 65.5 | | |
| Divorced or Widowed | 85 | 69.7 | | | 74 | 61.7 | | |
| Professional title | | | | | | | | |
| Junior | 4022 | 64.4 | 6.6 | 0.037 | 3217 | 66.4 | 1.5 | 0.482 |
| Intermediate | 1607 | 71.7 | | | 1068 | 67.3 | | |
| Senior | 434 | 66.7 | | | 254 | 64.1 | | |

For peer review only

| | | | | | | | | |
|---|---|---|---|---|---|---|---|---|
| Employment form | | | | | | | | |
| Regular staff | 3290 | 64.8 | 0.9 | 0.341 | 2213 | 66.3 | 0.1 | 0.733 |
| Temporary employee | 2673 | 65.8 | | | 2326 | 66.7 | | |
| Average monthly income (RMB) | | | | | | | | |
| ≤ 3000 | 2418 | 64.5 | 1.4 | 0.710 | 3275 | 66.6 | 0.3 | 0.955 |
| 3000–5000 | 2953 | 65.7 | | | 1233 | 66.0 | | |
| 5000–10000 | 574 | 65.8 | | | 29 | 69.0 | | |
| > 10000 | 18 | 64.3 | | | 2 | 66.7 | | |
| Department | | | | | | | | |
| Emergency | 418 | 81.3 | 101.2 | <0.001 | 429 | 82.2 | 158.9 | <0.001 |
| Internal medicine | 1889 | 66.3 | | | 1294 | 66.0 | | |
| Surgery | 1266 | 66.5 | | | 955 | 68.9 | | |
| Gynaecology & Obstetrics | 284 | 66.7 | | | 391 | 64.1 | | |
| Paediatrics | 351 | 68.0 | | | 483 | 77.0 | | |
| Other | 1755 | 59.8 | | | 987 | 57.4 | | |
| Years of experience | | | | | | | | |
| 1–4 | 2141 | 61.0 | 55.0 | <0.001 | 2006 | 64.2 | 35.2 | <0.001 |
| 5–10 | 1920 | 68.5 | | | 1148 | 71.9 | | |
| 11–20 | 1069 | 69.7 | | | 750 | 68.2 | | |
| ≥ 21 | 833 | 64.4 | | | 635 | 63.0 | | |
| Working time | | | | | | | | |
| 0–2 h | 87 | 53.0 | 26.1 | <0.001 | 32 | 61.5 | 67.9 | <0.001 |
| 2–4 h | 152 | 60.3 | | | 71 | 59.2 | | |
| 4–6 h | 165 | 62.5 | | | 53 | 46.9 | | |
| 6–8 h | 2367 | 63.7 | | | 1941 | 62.9 | | |
| > 8 h | 3192 | 67.2 | | | 2442 | 70.7 | | |
| Direct contact with patients | | | | | | | | |
| 0–2 h | 72 | 40.0 | 69.8 | <0.001 | 52 | 49.1 | 51.7 | <0.001 |
| 2–4 h | 127 | 53.6 | | | 144 | 57.4 | | |
| 4–6 h | 474 | 63.5 | | | 330 | 57.4 | | |
| 6–8 h | 5290 | 66.3 | | | 4013 | 68.1 | | |

**Characteristics of perpetrators and victims' responses**

Patients' relatives were the main perpetrators of WPV in both tertiary hospitals (83.1%) and county-level hospitals (85.2%). Most attacks on nurses occurred in the wards and during the day shift. More than 60% of the victims responded with tolerance, patience, and understanding (Table 4).

For peer review only

**Table 4** Characteristics of perpetrators and victims' responses

| | Tertiary hospitals (n=5963) | | County-level hospitals (n=4539) | |
|---|---|---|---|---|
| | n | % | n | % |
| Attack time | | | | |
| Day shift | 3558 | 59.7 | 2920 | 64.3 |
| Night shift | 1402 | 23.5 | 1195 | 26.4 |
| After work | 1003 | 16.8 | 424 | 9.3 |
| Attack site | | | | |
| Outpatient clinic | 388 | 6.5 | 351 | 7.7 |
| Ward | 2754 | 46.2 | 1933 | 42.6 |
| Doctor's office | 198 | 3.3 | 251 | 5.5 |
| Nurse's office or station | 1372 | 23.0 | 1291 | 28.5 |
| Treatment room | 127 | 2.1 | 162 | 3.6 |
| Others | 1124 | 18.9 | 551 | 12.1 |
| When the violent incident took place: | | | | |
| All alone | 1698 | 28.5 | 1319 | 29.1 |
| Other colleagues on the scene | 4265 | 71.5 | 3220 | 70.9 |
| Perpetrators * | | | | |
| Patients | 1784 | 35.9 | 1212 | 26.7 |
| Patients' relatives | 4131 | 83.1 | 3862 | 85.2 |
| Visitors | 748 | 15.0 | 573 | 12.6 |
| Others | 99 | 2.0 | 96 | 2.1 |
| Gender of the perpetrators* | | | | |
| Male | 3988 | 81.2 | 3380 | 83.1 |
| Female | 2461 | 50.1 | 1917 | 47.1 |
| Age group of the perpetrators (years)* | | | | |
| ≤ 20 | 258 | 5.1 | 258 | 5.7 |
| 21–30 | 1454 | 28.8 | 1624 | 35.9 |
| 31–40 | 2693 | 53.3 | 2263 | 50.0 |
| 41–50 | 2220 | 44.0 | 1529 | 33.8 |
| 51–60 | 917 | 18.2 | 1011 | 22.3 |
| ≥ 61 | 445 | 8.8 | 668 | 14.8 |
| Behavioral response to WPV * | | | | |
| Tolerance and avoidance | 3167 | 64.1 | 2601 | 63.3 |
| Patience and understanding | 2748 | 55.7 | 2415 | 58.8 |
| Give tit for tat | 80 | 1.6 | 36 | 0.9 |
| Try to explain before resorting to force | 438 | 8.9 | 310 | 7.5 |
| Ask colleagues for help | 940 | 19.0 | 537 | 13.1 |
| Turn to the managers or security staff for help | 1899 | 38.5 | 1334 | 32.5 |
| Ask for help from other patients and relatives | 287 | 5.8 | 165 | 4.0 |
| Call the police | 754 | 15.3 | 573 | 14.0 |
| Others | 166 | 3.4 | 72 | 1.8 |

* represents multiple choice

For peer review only

**Risk factors associated with WPV against nurses**

Participants' age, department, years of experience, and length of time in direct contact with patients were risk factors for WPV against nurses. Binary logistic regression analysis results demonstrated clearly that less than eight hours' working time is a protective factor against WPV for nurses in the county-level hospitals. Nurses in emergency departments were almost three times (OR = 2.993, 95%CI, 2.364 to 3.789) more likely to experience WPV in tertiary hospitals, and 3.387 times (95% CI, 2.648 to 4.332) more likely in county-level hospitals than nurses in other departments. Surprisingly, nurses with 5–10 years of experience were at the highest risk of WPV in county-level hospitals, while those with 11–20 years' experience were at the highest risk in tertiary hospitals. Further, our results showed that the likelihood of WPV rises with the length of treatment time in county-level hospitals (Table 5).

**DISCUSSION**

Previous studies have found that WPV exists in all hospitals but differs in some aspects.[27-31] The total incidence rate of WPV was 65.8% over the 12-month period. This is slightly higher than the figure of 64.48% reported for Guangzhou.[32] Compared with previous Chinese studies,[17-19] our study provides a comprehensive depiction of the incidence of WPV in Chinese comprehensive public hospitals. We have also documented the characteristics of the perpetrators and the coping style of nurses who experience WPV. This study demonstrates that WPV in China is higher than other countries. [33-36] Moreover, we found that WPV toward nurses is frequent, including verbal violence (64.9%), physical violence (11.8%), and sexual harassment (3.9%). This may be related to the fact that nurses are frequently in direct contact with patients and their relatives in their daily work. The findings also show that nurses in county-level hospitals are more likely to experience WPV than nurses in tertiary hospitals.

**Table 5** Risk factors associated with WPV against nurses in hospitals, binary logistic results *

| Variable name | | Tertiary hospitals (n=9142) | | | County-level hospitals(n=6828) | | |
|---|---|---|---|---|---|---|---|
| | | Adjusted OR | 95%CI | *P*-value | Adjusted OR | 95%CI | *P*-value |
| Age group (years) | ≥ 51 | 1.0 | reference | 0.044 | 1.0 | reference | 0.027 |
| | ≤ 30 | 1.500 | (1.092–2.061) | 0.012 | 1.606 | (1.115–2.314) | 0.011 |
| | 31–50 | 1.387 | (1.036–1.855) | 0.028 | 1.551 | (1.120–2.146) | 0.008 |
| Department | Other | 1.0 | reference | <0.001 | 1.0 | reference | <0.001 |
| | Emergency department | 2.993 | (2.364–3.789) | <0.001 | 3.387 | (2.648–4.332) | <0.001 |
| | Internal medicine | 1.313 | (1.178–1.463) | <0.001 | 1.408 | (1.227–1.615) | <0.001 |
| | Surgery | 1.341 | (1.187–1.514) | <0.001 | 1.644 | (1.414–1.912) | <0.001 |
| | Gynaecology and Obstetrics | 1.322 | (1.065–1.641) | <0.001 | 1.268 | (1.044–1.539) | 0.016 |
| | Paediatrics department | 1.433 | (1.172–1.753) | <0.001 | 2.391 | (1.934–2.956) | <0.001 |
| Years of experience | 1–4 | 1.0 | reference | <0.001 | 1.0 | reference | <0.001 |
| | 5–10 | 1.426 | (1.268–1.604) | <0.001 | 1.479 | (1.277–1.712) | <0.001 |
| | 11-20 | 1.627 | (1.358–1.951) | <0.001 | 1.300 | (1.045–1.618) | 0.018 |
| | ≥ 21 | 1.368 | (1.131–1.656) | 0.001 | 1.192 | (0.949–1.498) | 0.131 |
| Working time (hours) | > 8h | | | | 1.0 | reference | <0.001 |
| | 0–2h | | | | 0.852 | (0.469–1.548) | 0.599 |
| | 2–4h | | | | 0.762 | (0.507–1.145) | 0.191 |
| | 4–6h | | | | 0.492 | (0.329–0.735) | 0.001 |
| | 6–8h | | | | 0.720 | (0.647–0.800) | <0.001 |
| Direct contact with patients (hours) | 0–2h | 1.0 | reference | <0.001 | 1.0 | reference | <0.001 |
| | 2–4h | 1.832 | (1.230–2.728) | 0.003 | 1.201 | (0.744–1.938) | 0.454 |
| | 4–6h | 2.722 | (1.939–3.819) | <0.001 | 1.220 | (0.785–1.895) | 0.377 |
| | 6–8h | 3.054 | (2.247–4.152) | <0.001 | 1.710 | (1.134–2.580) | 0.011 |

* This analysis used data from 44 public tertiary hospitals and 90 public county-level hospitals in China.

For peer review only

For peer review only - http://bmjopen.bmj.com/site/about/guidelines.xhtml

This may be related to the level of education of patients and their relatives in county-level hospitals. In addition, the significant agricultural population in China is more likely to receive medical treatment at county-level hospitals, increasing the likelihood of WPV. In short, nurses have a high risk of experiencing WPV in the healthcare sector. [37-39]

Our survey also revealed that respondents aged 30 years or younger have a higher risk of WPV than their older colleagues. This phenomenon may be attributable to the fact that they have not been nurses for very long, and they lack work experience and communication skills. If that is the case, they need to practice their skills decrease the odds of making mistakes. Alternatively, because most of the in this age group were single children, they are quick to anger when criticized by patients and their relatives, increasing the possibility of WPV. In contrast, nurses who are aged 51 years or older have a lower risk of WPV. This may be related to inherent respect for the elderly, a traditional virtue of the Chinese nation for thousands of years.[40]

As shown in our survey, the risk of WPV is not the same in different departments. Substantial previous studies have found that emergency departments are likely to have the highest incidence of WPV, followed by pediatric departments.[24 41] This increased risk may be because emergency departments deal with the most serious patients in complex situations, such as traffic accidents, food poisoning, and patients with alcoholism. Meanwhile, patients' relatives may be very worried, and if nurses do not share information with patients and if the nurses' communication skills are inadequate, unnecessary conflicts may emerge. Pediatrics is another specialist department with a high risk of WPV. In China, all patients under the age of 14 are treated in pediatrics departments. The age range is different in countries around the world. The high risk of WPV in pediatrics departments may be because patients are likely to be the parents' only child, and to have been overindulged. This may make

For peer review only

nurses' work more difficult and increase the possibility of WPV. Therefore, hospitals and patients should make joint efforts to reduce violent incidents. On the one hand, hospitals can establish a code green response team, comprising a charge nurse, security personnel, and primary nurse to manage the potentially violent situation. Dilman's study demonstrated that 85% of code green calls resulted in successful resolution of the violent incidents.[42] Preventive measures for WPV among nurses include the following: increase awareness of potentially violent patients; wear suitable clothes; maintain proper positioning when communicating with patients; keep a safe distance; maintain the correct posture; and listen actively. A study by Hill et al. showed a 65% reduction in staff injuries, from 2.2 per week to 0.77 per week, during the 1-year intervention period.[43] On the other hand, patients and their relatives should show respect to others and understand that nurses' work is complex and professional. With mutual understanding, nurses and patients can help to improve the nurse-patient relationship and achieve harmony. Stievano et al.'s study has shown that patient care can be affected when nurses are not respected.[44]

This study also demonstrated that years of experience and extended direct contact with patients are risk factors for WPV. Medical treatment processes, waiting time for patients, nurses' attitudes, and other aspects of hospital procedure need improvement to enhance patients' satisfaction and reduce conflict. Previous studies have shown that strengthening hospital management can improve patient satisfaction.[45]

Perpetrators' characteristics are equally noteworthy. In the present study, perpetrators are primarily patients' family members, followed by patients. Perpetrators are usually males aged 31 to 50, with an inverted "U" shape distribution. The ward is the main site of WPV in hospitals, accounting for 46.2% and 42.6% in tertiary and county-level hospitals, respectively. Ward management needs strengthening:

For peer review only

for example, surveillance cameras and alarms equipment should be installed in hospital ward corridors, lights should be sufficiently bright in work areas during the night, and so on. Fine safety management can significantly improve the quality of nursing and patients' satisfaction, and reduce conflict.[46]

Although most patients and patients' relatives are well behaved, a few have underhand motives. Such people intentionally disrupt the normal medical order, and even pose a threat to the personal safety of medical staff and other patients, in order to seek monetary compensation. In China, a few years ago, the cost of "Yi Nao" (where in people employed by those in dispute with the hospital, together with family members of patients, took various measures to put pressure on the hospital, thereby profiteering) was very low. Occasionally, patients and their relatives may use illegal organizations to ask the hospitals for compensation directly, rather than use the normal legal procedure. Hospitals often make concessions by acquiescing to some unreasonable requirements to avoid trouble, preserve their reputation, and ensure normal medical order. This response has further contributed to the arrogance of those resorting to "Yi Nao." Fortunately, "Yi Nao" was officially criminalized in November 2015. The law now recognizes that "Yi Nao" risks the proper functioning of medical institutions and gives clear legal support to conviction and sentencing. Negative media coverage also has a huge impact on the physician-patient relationship. Therefore, we suggest that the government should supervise the media to ensure the accuracy and authenticity of media coverage. We further advise hospitals to strengthen the training and management of nurses to reduce the physical and psychological damage to them from WPV. For instance, hospitals could provide violence-related training for nurses and provide post-WPV psychological support or a "debriefing room," instruct all staff about the value of nurses and foster nurses' pride in their work, develop an excellent hospital culture, etc. If nurses have the support of managers, they may be more willing to consult psychologists,

For peer review only

psychiatrists, and mental health professionals to identify and treat any sudden disorder.[47]

We were surprised by victims' coping mechanisms, which were tolerance, avoidance, patience, and understanding in both tertiary and county-level hospitals. This response may reflect a culture of tolerance toward WPV in China. Most nurses turned to their managers or hospital security personnel for help when they experienced WPV. Thus, the organization's support and care may have significant potential for reducing the harm inflicted on nurses by WPV. Nurses should be familiar with all the resources in their work and family communities for solving any problems and should keep themselves in good mental health.[47]

The present study has several limitations. First, we collected data about whether nurses had experienced WPV over the previous 12 months, so there may be recall bias in the results. Second, we studied tertiary and county-level hospitals, but we did not study specialized hospitals. However, this study might be effective in preventing WPV in general and public hospitals.

In general, WPV can be prevented, according to the WHO's report on violence and health.[27] Therefore, to reduce violent incidents, we recommend that tertiary and county-level hospitals develop training against violence that is tailored to their particular situation, as well as the other measures we have suggested above. We also suggest improving psychological resilience for at-risk nurses to mitigate the negative impact of WPV and to prevent chronic diseases and reduce the incidence of mental illness.[48]

**CONCLUSIONS**

This study is based on a large sample survey of WPV in tertiary and county-level hospitals in China. The study has demonstrated that there is a high incidence rate of WPV and that occupational safety is

For peer review only

insufficient. The frequent occurrence of WPV in emergency and pediatric departments is also remarkable. The incidence of WPV of hospitals is an occupational health hazard and a serious threat to the wellbeing of nurses.

**Acknowledgements**

The authors thank nurses, managers, and the Chinese Hospital Association for their assistance and support for this project. We also acknowledge Xiaoli Jia and Min Ha for their assistance with the survey.

**Contributors**

LS and LF designed the study. DZ, CZ, TH, XP, YM and YH collected data. LS, LY, TS, LG and WL analysed the data. LS and LF drafted the manuscript. LS, LY, and LF revised the manuscript.

**Funding**

This study was funded by the Natural Science Foundation of China, grant number 71473063.

**Competing interests** None declared.

**Data sharing statement** No additional data are available.

**REFERENCES**

1. Ramacciati N, Ceccagnoli A, Addey B. Violence against nurses in the triage area: An Italian qualitative study. *Int Emerg Nurs* 2015;23(4):274-280.

2. Alkorashy HA, Al Moalad FB. Workplace violence against nursing staff in a Saudi university hospital. *INT NURS REV* 2016;63(2):226–232.

For peer review only

3. Fallahi K M, Oskouie F, Ghazanfari N, *et al.* The Frequency, Contributing and Preventive Factors of Harassment towards Health Professionals in Iran. *IJCBNM* 2015;3(3):156-164.

4. Pai DD, Lautert L, Souza SBCD, *et al*. Violence, Burnout and Minor Psychiatric Disorders in Hospital Work. *Rev Esc Enferm USP* 2015;49(3):457-464.

5. Fafliora E, Bampalis V G, Zarlas G, *et al.* Workplace violence against nurses in three different Greek healthcare settings. *Work* 2015;53(3):551-560.

6. Ferri P, Silvestri M, Artoni C, *et al.* Workplace violence in different settings and among various health professionals in an Italian general hospital: a cross-sectional study. *Psychology Research & Behavior Management* 2016;9:263-275.

7. Fute M, Mengesha ZB, Wakgari N, *et al*. High prevalence of workplace violence among nurses working at public health facilities in Southern Ethiopia. *BMC Nurs* 2015;14:9.

8. Mihyun P, Sung-Hyun C, Hyun-Ja H. Prevalence and Perpetrators of Workplace Violence by Nursing Unit and the Relationship Between Violence and the Perceived Work Environment. *J Nurs Scholarsh* 2014;47(1):87-95.

9. Al-Omari H. Physical and verbal workplace violence against nurses in Jordan. *Int Nurs Rev* 2015;62(1):111–118.

10. Schablon A, Zeh A, Wendeler D, *et al.* Frequency and consequences of violence and aggression towards employees in the German healthcare and welfare system: a cross-sectional study. *BMJ Open* 2011;2(5):421-421.

11. Hogarth KM, Beattie J, Morphet J. Nurses' attitudes towards the reporting of violence in the emergency department. *Australas Emerg Nurs J* Published Online First: 23 May 2015. doi: 10.1016/j.aenj.2015.03.006.


For peer review only

12. Shen HC, Cheng, Y, Tsai PJ, *et al*. Occupational stress in nurses in psychiatric institutions in Taiwan. *J Occup Health* 2005;47:218–225.

13. Pompeii LA, Schoenfisch AL, Lipscomb HJ, *et al*. Physical assault, physical threat, and verbal abuse perpetrated against hospital workers by patients or visitors in six U.S. hospitals. *Am J Ind Med* 2015;58(11):194-204.

14. Hahn S, Muller M, Hantikainen V, *et al*. Risk factors associated with patient and visitor violence in general hospitals: results of a multiple regression analysis. *Int J Nurs Stud* 2013;50(3):374-85.

15. Albashtawy M, Aljezawi M. Emergency nurses' perspective of workplace violence in Jordanian hospitals: A national survey. *Int Emerg Nurs* 2015;24:61-65.

16. Jia XL, Zhou HZ, Zhao Y, *et al*. Investigation on hospital violence during 2003 to 2012 in China. *Chinese Hospitals* 2014;18(3):1-3.

17. Zhao Y, Wang FM, Zhou CY, *et al*. Investigation and analysis of present situation of workplace violence at tertiary hospitals and county hospitals in Heilongjiang Province. *Medicine and Society* 2016;29(9):35-37.

18. Yan JH, Hou J, Lin HQ, *et al*. Workplace violence in hospital setting to nursing staff and its influencing factors. *Journal of Nursing (China)* 2012;19(8A):4-7.

19. Chen CR, Duan XG. The causes of workplace violence on nursing and its corresponding countermeasures. *Chinese Journal of Nursing* 2010;16(5):572-574.

20. Liu L, Yang YL, Wang ZY, *et al*. Prevalence and Positive Correlates of Posttraumatic Stress Symptoms among Chinese Patients with Hematological Malignancies: A Cross-Sectional Study. *Plos One* 2015;10(12).

21. Wang X, Jing ZR, Zhao BL, *et al*. Survey on workplace violence and job burnout among nurses. *J*

For peer review only

*Nurs Sci* 2015;(12):15-17.

22. Itzhaki M, Peles-Bortz A, Kostistky H, *et al.* Exposure of mental health nurses to violence associated with job stress, life satisfaction, staff resilience, and post-traumatic growth. *International Journal of Mental Health Nursing* 2015;24(5):403–412.

23. Yang JJ, Gao LL. A survey on willingness of nurses' children engage in medical work. *Education Teaching Forum* 2015;(3):73-74.

24. Hahn SZA, Needham IEA. Patient and visitor violence in general hospitals: a systematic review of the literature. *Aggression Violent Behav* 2008;13:431–41.

25. Chen ZH, Wang SY. Psychiatric hospital and general hospital workplace violence study comparison. *Chinese Journal of Public Health* 2004;20:1316–1317.

26. ILO/ICN/WHO/PSI. Framework Guidelines for Addressing Workplace Violence in the Health Sector.Geneva: ILO/ICN/WHO/PSI Joint Programme on Workplace Violence in the Health Sector 2002:4-132.

27. Laschinger HK, Grau AL. The influence of personal dispositional factors and organizational resources on workplace violence, burnout, and health outcomes in new graduate nurses: A cross-sectional study. *Int J Nurs Stud* 2012;49:282–291.

28. Kitaneh M, Hamdan M. Workplace violence against physicians and nurses in Palestinian public hospitals: A cross-sectional study. *BMC Health Serv Res* 2012;12:469.

29. Branko G, Ivan E. Prevalence of violence towards nursing staff in Slovenian nursing homes. *Slovenian Journal of Public Health* 55(3):212-217.

30. Merecz D, Rymaszewska J, Moscicka A, *et al*. Violence at the workplace-a questionnaire survey of nurses. *Eur Psychiatry* 2006;21:442–450.

For peer review only

31. Ferns T. Under-reporting of violent incidents against nursing staff. *Nurs Stand* 2006;20(40):41–45.

32. Chen ZH, Wang SY, Jing CX. Investigation on workplace violence phenomenon of two hospitals in Guangzhou. *Chinese Journal of Preventive Medicine* 2003;37:358–360.

33. Zampieron A, Galeazzo M, Turra S, *et al.* Perceived aggression towards nurses: study in two Italian health institutions. *J Clin Nurs* 2010;19:2329–41.

34. Tiruneh BT, Bifftu BB, Tumebo A A, *et al.* Prevalence of workplace violence in Northwest Ethiopia: a multivariate analysis. *BMC Nursing*, 2016;15(1):1-6.

35. Franz S, Zeh A, Schablon A, *et al*. Aggression and violence against health care workers in Germany–a cross sectional retrospective survey. *BMC Health Serv Res* 2010;10:51.

36. Hahn S, Muller M, Needham I, *et al.* Factors associated with patient and visitor violence experienced by nurses in general hospitals in Switzerland: a cross-sectional survey. *J Clin Nurs* 2010; 19:3535–46.

37. Magin PJ, May J, McElduff P. Occupational violence in general practice: a whole-of-practice problem. Results of a cross-sectional study. *Australian Health Review* 2011;35(1):75–80.

38. Kwok RP, Law YK, Li KE, *et al*. Prevalence of workplace violence against nurses in Hong Kong. *Hong Kong Med J* 2006;12:6–9.

39. Tiruneh BT, Bifftu BB, Tumebo AA, *et al.* Prevalence of workplace violence in Northwest Ethiopia: a multivariate analysis. *BMC Nursing* 2016;15(1):1-6.

40. Sun T, Wang S, Wang N, *et al*. Epidemiological characteristics and variance analysis of workplace violence in public hospitals. *Practical Preventive Medicine* 2014;21(4):478-480.

41. Shaw J. Staff perceptions of workplace violence in a pediatric emergency department. *Work*

For peer review only

2015;51(1):39-49.

42. Dilman Y. Code green for workplace violence. *Critical Care Nurse* 2015;35:34-35.

43. Hill AK, Lind MA, Tucker D, *et al*. Measurable results: Reducing staff injuries on a specialty psychiatric unit for patients with developmental disabilities. *Work* (*Reading, Mass.*) 2015;51(1): 99-111.

44. Stievano A, Bellass S, Rocco G, *et al*. Nursing's professional respect as experienced by hospital and community nurses. *Nursing Ethics* 2016.

45. Al Fraihi KJ, Latif SA. Evaluation of Outpatient Service Quality in Eastern Saudi Arabia. Patient's expectations and perceptions. *Saudi Medical Journal* 2016;37(4):420-428.

46. Mei XM. Application of Fine Management in Safety Management in Geriatric Ward. *Hospital Management Forum* 2016.

47. Teris C, Yip PSF. Depression, anxiety and symptoms of stress among Hong Kong nurses: a cross-sectional study. *Inter J Env Res Pub Heal* 2015;12(9):11072-11100.

48. Teris C, Yip PSF. Lifestyle and Depression among Hong Kong Nurses. *Inter J Env Res Pub Heal*, 2016;13(1):135.

For peer review only

# A survey on patient and visitor violence experienced by nurses in general hospitals of China

Dear nurses:

Hello! In recent years, nurses suffered suffered more and more serious violence. This study is based on the perspective of the nurses, understanding the nurses in the violence in the process of experience, violence after the occurrence of physical and mental injury to nurses and the views of the hospital violence. In order to safeguard the legitimate rights and interests of nurses , maintain the normal order of the hospital, we will conduct a "hospital workplace Violence "situation investigation.The survey related to any of your personal information, we will be for you to keep confidential, please rest assured!

**Note: Please tick the appropriate box, some problems can select multiple items, part of the problem, please fill in the "_____".**

Name_____________ Contact information_____________

**Part1：Please indicate your information**

Q1.Gender: Male□ Female□

Q2.Age: ≤30years□ 31-40years□ 41-50years□
51-60years□ ＞60years□

Q3.Level of education: ＜Bachelor□ Bachelor□ ≥Master□

Q4.Marital status: Married□ Single□ Other□

Q5.Professional title: Junior□ Intermediate□ Senior□

Q6.Employment form: formal employee□ temporary employee□

Q7.Monthly income(RMB):
＜3000□ 3000-5000□ 5000-10000□ ＞10000□

Q8.Department: Emergency□ Inter Medical□ Surgical□
Oncology□ Orthopedic□ Gynaecology□
Pediatrics□ Stomatology□ Ophthalmic□
ENT□ Medical Imaging□ Other□

Q9.Years of Working: ＜1years□ 1-4years□ 5-10years□
11-20years□ ＞20years□

Q10.Treatment Time: 0-2hours□ 2-4hours□ 4-6hours□
6-8hours□ ＞8hours□

Q11.The contact time with the patient: 0-2□ 2-4□ 4-6□ 6-8□

**Part2：In the past 12 months, have you ever encountered the following in the hospital?**

| Types of violence | | | | |
|---|---|---|---|---|
| Psychological violence | No | 1 times | 2-3 times | ≥4 times |
| 1. Scold, abuse, abusive, demeaning or other detrimental to the dignity of the individual words. (face to face, telephone, letter or leaflets, micro-network, etc). | | | | |

| | | | | |
|---|---|---|---|---|
| 2.Threats: threats related to personal and property safety, threatening complaints, trailing tracking and so on. | | | | |
| 3.Sexual harassment or sexual provocation, including language, movement or exposure of sexual organs. | | | | |
| Physical violence(To physical contact or something to attack, including playing, kick, push, bite, throw objects and so on). | | | | |
| 4.Causing mild injury, such as pain, abrasions, scratches, or ecchymosis. | | | | |
| 5.Resulting in significant injury, such as wounds, fractures, visceral or head injury | | | | |
| 6.Causing serious consequences, such as dysfunction or permanent disability | | | | |
| 7.Sexual assault, such as by the opposite sex to pull, hold, kiss or touch the sensitive parts. | | | | |
| 8.Rape or attempted rape | | | | |

**Δ If you do not have the above experience, please skip to the fourth part of the beginning to continue to answer.**

**Part3: Please describe the most profound event in your memory over the last 12 months.**

1.Location of the incident:

Outpatient department□ Ward□ Doctor's Office□

Nursing station/office□ Treatment room□ Other□

2.Time of occurrence：Day shift□ Night shift□ After work□

3.When the event occurs, you are：

Alone□ There are other colleagues present□

4.The perpetrators are (can choose more than one):

Patient□ Family members□ Visitors□ Other□

5.Perpetrators of gender (If more than one person, can choose more than one): Male□ Female□

6.Perpetrators of age (If more than one person, can choose more than one):

≤20years□ 21-30years□ 31-40years□

41-50years□ 51-60years□ ＞60years□

7. When this event occurs, what do you think the following circumstances compared with the perpetrators? (you can choose more than one)

Patient disability or death□ Drinking and drug abuse□

Patients with dysfunction or complications□ Mental disorders□

Long waiting time for treatment□ Low education level□

Unreasonable demands are rejected□

Not satisfied with treatment effect□

Not satisfied with the service attitude of medical staff□

The hospital service process is cumbersome□
Medical expenses are expensive□
Want to seek economic compensation□ Other______________
8.How do you choose to respond to such incidents? (you can choose more than one)
Tolerance and avoidance□ Patience and understanding□
Give tit for tat（such as sparring、insults）□
After the first reasonable counterattack□ Ask a colleague for help□
Ask a managers or security for help□ Call the police□
Ask a other patients or family member for help□ Other□
9.The impact of the incident on your psychological and behavioral? (you can choose more than one)
No effect□ Unable to concentrate□ Aggrievement□
Angry□ Anxious□ Insomnia□ Depressed□
Work enthusiasm decline□ Work efficiency decline□
Hate and fear of patients and their families□
Interpersonal communication barriers□ Have turnover intention□
Have suicidal thoughts□ Scene repetition□ Other□
10.Because of this event to see a doctor？
Psychological counseling □Routine treatment□ No□

**Part4: Perceptions and attitudes of workers towards workplace violence**

1.Have you heard of the term " hospital workplace violence "?
Yes□ No□
2.Do you think "hospital workplace violence" is inevitable in medical work? Yes□ No□
3.Do you think the "hospital workplace violence" is not worth the fuss?
Yes□ No□
4.What do you think should be included the content of the prevention of hospital workplace violence in the medical post training?
Yes□ No□
5.Are you willing to take part in the training of the hospital's workplace violence Prevention related knowledge? Yes□ No□
6.Do you think it will benefit you to take part in the prevention of hospital workplace violence? Yes□ No□
7.Do you think the hospital is designed to deal with the "hospital workplace violence " institutions or organizations to prevent hospital violence is effective? Yes□ No□
8.Which of the following measures can be taken to prevent or reduce the occurrence of hospital workplace violence? (you can choose more than one)
(1)Government for hospital violence legislation□
(2)Participate in medical liability insurance□
(3)Through third-party mediation□ (4)Outpatient and ward installation camera□
(5)Reduce the workload of medical services□
(6)Improve the environment for medical treatment□

For peer review only

(7)Optimize service flow□ (8)Improve service attitude□
(9)Improve interpersonal communication skills□
(10)To carry out targeted training□
(11)Strengthen the ability to deal with violence□
(12)To improve the service level and treatment ability□
(13)Increase security personnel□
(14)Public security institutions stationed in hospital□
(15) Media correct guidance of public opinion□
(16) Advocating social respect for medical workers□
(17) Other__________________

**Part5: Hospital attitudes to workplace violence and intervention strategies.**

1.Does your hospital have staff training related to hospital workplace violence prevention? Yes□ No□

2.What are the measures taken by your hospital to prevent hospital workplace violence?
(1)Regular security patrols throughout the day□
(2)Installation of cameras in Outpatient and ward□
(3)Installation of alarm equipment in Outpatient and ward□
(4)Setting a fixed waiting area seat□ (5) third-party mediation□
(6)The establishment of medical liability insurance□
(7)Public security departments stationed□ (8)Other______________

3.Does your facility encourage employees to report in a timely manner after being exposed to hospital workplace violence? Yes□ No□

4.Does your hospital have specialized departments to handle hospital workplace violence for employees? Yes□ No□

5.Do you think it is reasonable for your hospital to handle hospital workplace violence in the past? Yes□ No□

6.Do you think it is timely for your hospital to handle hospital workplace violence in the past? Yes□ No□

7.The attitude of the hospital to employees who suffered from workplace violence:
(1)Actively protect the interests of employee□ (2)Basing on facts and fair dealing□
(3)Neither stand nor actively□ (4)Punishing employees□

8.The degree of attention of hospital after doctors and nurses exposed to violence:
(1)Paying more attention to the doctor□ (2)Paying more attention to the nurse□
(3)Consistent the degree of attention□

**Thank you very much for your assistance, please check the above items are not missing fill !**

For peer review only

!

1. the National Natural Science Foundation of China, grant number71473063

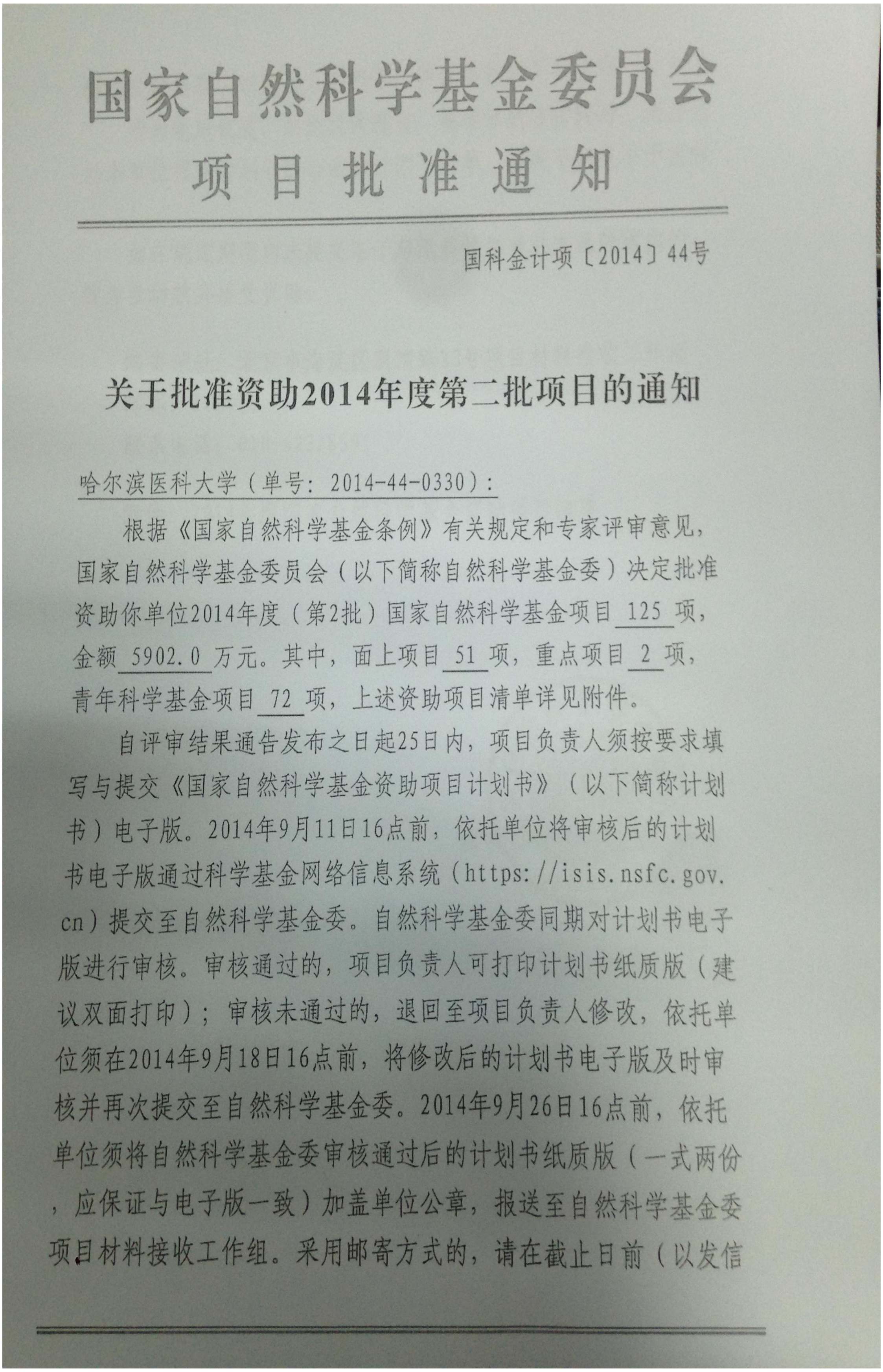

# 国家自然科学基金委员会

## 项目批准通知

国科金计项〔2014〕44号

### 关于批准资助2014年度第二批项目的通知

哈尔滨医科大学（单号：2014-44-0330）：

根据《国家自然科学基金条例》有关规定和专家评审意见，国家自然科学基金委员会（以下简称自然科学基金委）决定批准资助你单位2014年度（第2批）国家自然科学基金项目 125 项，金额 5902.0 万元。其中，面上项目 51 项，重点项目 2 项，青年科学基金项目 72 项，上述资助项目清单详见附件。

自评审结果通告发布之日起25日内，项目负责人须按要求填写与提交《国家自然科学基金资助项目计划书》（以下简称计划书）电子版。2014年9月11日16点前，依托单位将审核后的计划书电子版通过科学基金网络信息系统（https://isis.nsfc.gov.cn）提交至自然科学基金委。自然科学基金委同期对计划书电子版进行审核。审核通过的，项目负责人可打印计划书纸质版（建议双面打印）；审核未通过的，退回至项目负责人修改，依托单位须在2014年9月18日16点前，将修改后的计划书电子版及时审核并再次提交至自然科学基金委。2014年9月26日16点前，依托单位须将自然科学基金委审核通过后的计划书纸质版（一式两份，应保证与电子版一致）加盖单位公章，报送至自然科学基金委项目材料接收工作组。采用邮寄方式的，请在截止日前（以发信

邮戳日期为准）以快递方式邮寄，并在信封左下角注明“计划书”。请勿使用包裹，以免延误报送。报送计划书材料时，还应包括本单位报送计划书的公函和计划书清单。材料不完整不予接收。

如在规定期限内未提交电子与纸质计划书且未说明理由的，视为自动放弃接受资助。

邮寄地址：北京市海淀区双清路83号项目材料接收工作组
邮编：100085
联系电话：010-62328591

附件：2014年度国家自然科学基金资助项目清单

国家自然科学基金委员会
2014年8月25日

# 2014年国家自然科学基金资助项目清单（哈尔滨医科大学）

单号：2014-44-0330

资助金额单位：万元

| 序号 | 项目批准号 | 负责人 | 申请代码 | 项目名称 | 资助金额 | 起止日期 | 资助类别/亚类说明/附注说明 |
|---|---|---|---|---|---|---|---|
| 12 | 61403112 | 刘洪波 | F030406 | 基于高通量组学数据的干细胞多能性相关DNA甲基化调控元件识别及其功能分析 | 25 | 2015.01.01-2017.12.31 | 青年科学基金项目 |
| 13 | 61473106 | 李霞 | F030407 | 癌症非编码miRNA-lncRNA协同调控网络重构与功能特性研究 | 82 | 2015.01.01-2018.12.31 | 面上项目/常规面上项目 |
| 14 | 71403071 | 孙涛 | G0308 | 基于生态理论视角下区域医疗联合体生态系统健康评价建模与实证研究 | 23 | 2015.01.01-2017.12.31 | 青年科学基金项目 |
| 15 | 71403072 | 王月枫 | G0308 | 社区高血压患者急症风险预测模型构建及预见性干预与救护管理体系的研究 | 20 | 2015.01.01-2017.12.31 | 青年科学基金项目 |
| 16 | 71403073 | 李叶 | G0308 | 基于CHE方法学比较分析的医保制度经济保护能力影响因素研究 | 23 | 2015.01.01-2017.12.31 | 青年科学基金项目 |
| 17 | 71403074 | 尹慧 | G0308 | 基于结构方程的社区心血管疾病的社会决定因素模型构建及分层干预效果评价 | 20 | 2015.01.01-2017.12.31 | 青年科学基金项目 |
| 18 | 71473063 | 樊立华 | G0308 | 医护人员遭受医院场所暴力PTSD及对职场行为影响机制研究 | 63 | 2015.01.01-2018.12.31 | 面上项目/常规面上项目 |
| 19 | 71473064 | 焦明丽 | G0308 | 基于DEMATEL 法与"破窗理论"的医院暴力影响因素及对策研究 | 60 | 2015.01.01-2018.12.31 | 面上项目/常规面上项目 |
| 20 | 71473065 | 宁宁 | G0308 | 面向公共卫生灾害的城市社区抗逆力理论、方法及策略研究 | 63 | 2015.01.01-2018.12.31 | 面上项目/常规面上项目 |
| 21 | 81400097 | 高春艳 | H0811 | 血管内皮细胞清除活化和凋亡的血小板调控血栓形成的作用与机制 | 23 | 2015.01.01-2017.12.31 | 青年科学基金项目 |
| 22 | 81400115 | 付玥玥 | H0812 | APL分化综合征miRNA标记筛选及功能分析 | 23 | 2015.01.01-2017.12.31 | 青年科学基金项目 |

第2页 / 共12页

**2. Ethical Approval:**

## 哈尔滨医科大学公共卫生学院医学伦理审查申请表

| | |
|---|---|
| 项目名称：医护人员遭受医院暴力 PTSD 及对职场行为影响机制研究 | |
| 项目负责人：樊立华 | 职称：教授 |
| 电话：13136661393 | 电子信箱：lihuafan@126.com |
| 所在单位:哈尔滨医科大学公共卫生学院 | |

单位伦理委员会意见：

按照国家、省有关部门医学伦理审查的规定和程序，哈尔滨医科大学公共卫生学院医学伦理委员会对本申请的项目审查结论为：研究项目所涉及的医学伦理内容和实施的解决方案符合要求。

伦理委员会成员签字：

伦理委员会主任签字： 单位伦理委员会公章：

2014年 3月 1日

哈尔滨医科大学医学伦理委员会

1

Checklist of ***cross-sectional studies***

| | Page number, line number |
|---|---|
| **Title** | 1, 4-6 |
| **Abstract** | 2, 4-58 |
| **Strengths and limitations of this study** | 3, 4-27 |
| **Introduction** | |
| Background | 4, 4-57; 5, 4-14 |
| Objectives | 5, 16-24 |
| **Methods** | |
| Study design | 5, 37-52 |
| Setting | 5, 52-57; 6, 4-19 |
| Participants | 6, 21-27 |
| Questionnaire | 6, 41-57; 7, 4-29 |
| Data measurement | 7, 36-57; 8, 4 |
| Study size | 6, 28-32 |
| Quantitative variables | 7, 36-42 |
| Ethical considerations | 8, 11-19 |
| **Results** | |
| Participants | 8, 26-34 |
| Descriptive data | 8, 36-58; 9, 3-48 |
| Outcome data | 9, 52-57 |
| Main results | 10; 11; 12;14 |
| Other analyses | 10, 8-15; 12 |
| **Discussion** | |
| Key results | 13,32-57; 15, 4-56 |
| Interpretation | 16, 3-32; 17, 9-57 |
| Discuss the external validity of the study results | 17, 9-27 |
| **Conclusions** | 18, 51-57; 19, 4-7 |
| **Other information** | |
| Acknowledgements | 18, 14-19 |
| Contributors | 18, 24-27 |
| Funding | 18, 34-36 |
| Competing interests | 18, 34 |
| Ethical approval | 18, 47-52 |
| **References** | 19; 20; 21; 22; 23; 24 |

For peer review only

BMJ Open

# A cross-sectional study on the prevalence and associated risk factors for workplace violence against Chinese nurses

| | |
|---|---|
| Journal: | *BMJ Open* |
| Manuscript ID | bmjopen-2016-013105.R3 |
| Article Type: | Research |
| Date Submitted by the Author: | 14-Mar-2017 |
| Complete List of Authors: | Shi, Lei; School of Public Health, Harbin Medical University, Department of Health Management<br>Zhang, Danyang; The First Affiliated Hospital of Harbin Medical University, Operating Section<br>Zhou, Chenyu; School of Public Health, Harbin Medical University, Department of Health Management<br>Yang, Libin; School of Public Health, Harbin Medical University, Department of Medical Education<br>Sun, Tao; School of Public Health, Harbin Medical University, Department of Health Management<br>Hao, Tianjun; The Second Affiliated Hospital of Harbin Medical University, Department of Scientific Research<br>Peng, Xiangwen ; The First Affiliated Hospital of Harbin Medical University, Harbin, Operating Section<br>Gao, Lei; School of Public Health, Harbin Medical University, Department of Health Management<br>Liu, Wenhui; School of Public Health, Harbin Medical University, Department of Medical Education<br>Mu, Yi; Beijing Children's Hospital, Capital Medical University, Department of Customer Service<br>Han, Yuzhen; The Fourth Affiliated Hospital of Harbin Medical University, Department of Medical Disputes<br>Fan, Lihua; School of Public Health, Harbin Medical University, Department of Health Management |
| <b>Primary Subject Heading</b>: | Health services research |
| Secondary Subject Heading: | Health policy, Medical management, Nursing |
| Keywords: | workplace violence (WPV), nurses, general and public hospitals, risk factors |
| | |

SCHOLARONE™ Manuscripts

# A cross-sectional study on the prevalence and associated risk factors for workplace violence against Chinese nurses

Lei Shi,[1] Danyang Zhang,[2] Chenyu Zhou,[1] Libin Yang,[3] Tao Sun,[1] Tianjun Hao,[4] Xiangwen Peng,[2] Lei Gao,[1] Wenhui Liu,[3] Yi Mu,[5] Yuzhen Han,[6] Lihua Fan[1]

**Correspondence to:** Lihua Fan, School of Public Health, Harbin Medical University, 157 Baojian Road, Nangang District, Harbin 150081, China; E-mail: lihuafan@126.com

[1]Department of Health Management, School of Public Health, Harbin Medical University, Harbin, China

[2] Operating Section, The First Affiliated Hospital of Harbin Medical University, Harbin, China

[3]Department of Medical Education, School of Public Health, Harbin Medical University, Harbin, China

[4]Department of Scientific Research, The Second Affiliated Hospital of Harbin Medical University, Harbin, China

[5]Department of Customer Service, Beijing Children's Hospital, Capital Medical University, Beijing, China

[6]Department of Medical Disputes, The Fourth Affiliated Hospital of Harbin Medical University, Harbin, China

For peer review only

**Abstract**

**Objectives:** The purpose of the present study was to explore the characteristics of workplace violence that Chinese nurses at tertiary and county-level hospitals encountered in the 12 months from December 2014 to January 2016, to identify and analyze risk factors for workplace violence, and to establish the basis for future preventive strategies.

**Design:** A cross-sectional study.

**Setting:** A total of 44 tertiary hospitals and 90 county-level hospitals in 16 provinces (municipalities or autonomous regions) in China.

**Methods:** We employed stratified random sampling to collect data from December 2014 to January 2016. We distributed 21,360 questionnaires, and 15,970 participants provided valid data (effective response rate = 74.77%). We conducted binary logistic regression analyses on the risk factors for workplace violence among the nurses in our sample and analyzed the reasons for aggression.

**Results:** The prevalence of workplace violence was 65.8%; of this, 64.9% was verbal violence, and physical violence and sexual harassment accounted for 11.8% and 3.9%, respectively. Frequent workplace violence occurred primarily in emergency and pediatric departments. Respondents reported that patients' relatives were the main perpetrators in tertiary and county-level hospitals. Logistic regression analysis showed that respondents' age, department, years of experience, and direct contact with patients were common risk factors at different levels of hospitals.

**Conclusions**: Workplace violence is frequent in China's tertiary and county-level hospitals; its occurrence is especially frequent in the emergency and pediatric departments. It is necessary to cope with workplace violence by developing effective control strategies at individual, hospital, and national levels.

For peer review only

**Strengths and limitations of this study**

- We used a large nurse-based sample in our study.
- Sample selection was reasonable and representative, and was distributed in the eastern, middle, and western regions of China.
- Our study compared differences between nurses who had experienced workplace violence in comprehensive public tertiary and county-level hospitals.
- The study provides the basis for establishing operational guidelines for preventing workplace violence in China.
- The retrospective approach to self-reported workplace violence used by respondents may cause recall bias.

For peer review only

**BACKGROUND**

Workplace violence (WPV) towards health service professionals is recognized as a global public health issue, and it has attracted worldwide attention.[1-4] Previous studies have suggested that health professionals have a higher risk of experiencing WPV than any other professionals.[2-3] Further, the incidence rate of WPV differs among nurses in various countries; for instance, the incidence was 76.0% in Greece, 82% in Pakistan, and 67% in Italy.[5-6]

Several substantial studies have suggested that nurses have a high risk of experiencing WPV.[6-10] During the past 12 months, the incidence rate of physical violence for nurses in Ethiopia,[7] South Korea,[8] Jordan,[9] Germany,[10] and Iran[3] ranged from 18.22% to 56.0%, the verbal abuse rate being from 63.8% to 89.58% and the sexual harassment rate from 4.7% to 19.7%. WPV occurs primarily in the emergency wards and psychiatric departments of hospitals.[11-12] Research into the experience of nurses in these departments in the US,[13] Switzerland,[14] and Jordan[15] has demonstrated that they experience a higher incidence of WPV than do nurses in other departments.

In China, WPV in hospitals has increased gradually over the past few decades. According to the report from the Chinese Hospital Association, the proportion of hospitals experiencing WPV rose from 90% in 2008 to 96% in 2012 and the prevalence of sexual harassment has increased year by year.[16]

Most previous studies of WPV against nurses in China have been conducted in the provinces, and the samples are not sufficiently representative: they do not present an accurate picture of the incidence of WPV against nurses in Chinese general hospitals.[17-19] In this study, WPV is divided into physical violence and psychological violence, which includes verbal violence and sexual harassment in accordance with the definition of WPV in hospitals used by the World Health Organization (WHO), the International Labour Office (ILO), and the specific situation in China.

For peer review only

Although violent incidents in hospitals directly harm the healthcare workers and hospitals, the ultimate victims are the patients.[20] WPV affects the normal functioning and reputation of hospitals and threatens the personal safety of healthcare workers and patients.[21] Moreover, violent incidents have a negative impact on the psychological welfare of the healthcare workers,[22] who do not want their children to be engaged in healthcare.[23]

The purpose of the present study was to explore the distribution and characteristics of WPV experienced by Chinese nurses at tertiary and county-level hospitals in the 12 months from December 2014 to January 2016, to identify and analyze the risk factors for WPV, thus providing a basis for future preventive strategies.

## METHODS

### Sample and procedure

A cross-sectional survey was designed based on the geographical location and level of economic development in the eastern (Beijing, Tianjin, Hebei, Shandong, Guangdong, Liaoning), middle (Shanxi, Henan, Anhui, Hunan, Heilongjiang), and western regions of China (Ningxia, Shannxi, Gansu, Sichuan, Chongqing). In 2015, there were 3,069 general and public hospitals in China and approximately 920,700 registered nurses in public tertiary and county-level hospitals. We selected a sample of 21,360, approximately 2.30% of all nurses. In order to select the same proportion of the workforce from each department, we sought to recruit 120 nurses from the Departments of Internal Medicine and Surgery, 80 nurses from the Departments of Emergency, Neurology, Obstetrics and Gynecology, and Pediatrics, and 40 nurses from the Departments of Stomatology, Ophthalmology, and Ear, Nose and Throat in each tertiary hospital. We distributed 10,560 questionnaires to 44 tertiary hospitals. We sought to recruit

For peer review only

nurses at county-level hospitals in the same proportions: 60 from the Departments of Internal Medicine and Surgery, 40 from the Departments of Emergency, Neurology, Obstetrics and Gynecology, and Pediatrics, and 20 from the Departments of Stomatology, Ophthalmology, and Ear, Nose and Throat at each county-level hospital. We distributed 10,800 questionnaires to the 90 county-level hospitals.

All 134 hospitals agreed to participate in the study. The inclusion criteria for participants were 1) voluntary participation by nurses engaged in clinical work who 2) had at least one year of professional experience in hospitals.

The survey was conducted from December 2014 to January 2016. We obtained permission from the managers and human resources departments of the hospitals. The researchers used a stratified random sampling method to collect the data. Each participant had two days to complete the self-administered questionnaire. Collected data were kept confidential and used only for academic research. We distributed 21,360 questionnaires and received 15,970 valid questionnaires (effective response rate = 74.77%).

## QUESTIONNAIRE

We developed the questionnaire based on three documents. First, we used the revised Survey of Violence Experienced by Staff (SOVES-G-R) developed by Hahn and colleagues to formulate the questionnaire.[24] The revised SOVES-G-R was used to measure perpetrators' characteristics and the way to deal with violence for nurses. Next, we used items from the Chinese version of the Workplace Violence Scale (WVS) in the literature (the frequency of violence, different levels of the severity of physical violence, and hospital attitudes to WPV and intervention strategies) to develop the questionnaire.[25] Chen and Wang's study incorporated the levels of the severity of physical violence,

For peer review only

and hospital attitudes to WPV and intervention strategies. In addition, we used the questionnaires from the ILO, ICN (International Council of Nurses), WHO, and PSI (Public Services International) joint program for measuring WPV (e.g., types of WPV, time and place the violence occurred, and perceptions and attitudes of nurses towards WPV).[26] We pre-tested the questionnaire with 367 nurses from three public tertiary and two public county-level hospitals in Heilongjiang Province and revised the questionnaire post-test. Further, we invited 18 healthcare-related experts to assess the accuracy, comprehensiveness, and sensitivity of the items in the questionnaire. In order to ensure the reliability of the revised questionnaire according to expert opinion, we selected 835 nurses from five public tertiary and four public county-level hospitals in Heilongjiang Province to measure the reliability of the questionnaire again. We measured the questionnaire's reliability twice using SPSS19.0; Cronbach's alpha coefficient was 0.84 and 0.86, respectively.

**DATA ANALYSIS**

EpiData version 3.1 was used to establish the study's database. To ensure accuracy, the data were checked by trained personnel after all surveys were completed and entered. We used IBM SPSS19.0 and Excel for statistical analysis of the relevant quantitative data. Univariate analysis and chi-square tests ($\chi^2$) were used to analyze the relationship between the demographic characteristics of respondents and the incidence rate of WPV. We employed logistic regression analysis to test the relationship between nurses experiencing hospital WPV as the dependent variable, and the variables with statistical and clinical practice significance as the covariates. We conducted binary logistic regression analyses on the risk factors for WPV among the nurses in our sample. Odds ratios (OR) and 95% confidence intervals (CI) were calculated; $\alpha = 0.05$ was the test and $P < 0.05$ was considered statistically

For peer review only

significant.

## ETHICAL CONSIDERATIONS

Ethical approval to undertake this study was granted by the Research Ethics Committee of Harbin Medical University in March 2014. We obtained consent from each hospital involved in the research processes. All participants gave informed consent to the researchers or to their head nurses before the survey, and participants' personal information was kept confidential.

## RESULTS

**Participants' demographic characteristics**

We received 17,865 responses, and 15,970 respondents met our inclusion criteria. Most respondents had completed undergraduate studies and were less than 30 years old; however, the number of female respondents was significantly higher compared to male respondents (Table 1).

**Table 1** Participants' demographic characteristics (N=15970)

| Characteristics | Tertiary hospitals (n=9142) | | County-level hospitals (n=6828) | | Total (n=15970) | |
|---|---|---|---|---|---|---|
| | n | % | n | % | n | % |
| Gender | | | | | | |
| Male | 234 | 2.6 | 143 | 2.1 | 377 | 2.4 |
| Female | 8908 | 97.4 | 6685 | 97.9 | 15593 | 97.6 |
| Age (years) | | | | | | |
| ≤30 | 5131 | 56.1 | 4033 | 59.1 | 9164 | 57.4 |
| 31–50 | 3785 | 41.4 | 2611 | 38.2 | 6396 | 40.0 |
| ≥51 | 226 | 2.5 | 184 | 2.7 | 410 | 2.6 |
| Educational level | | | | | | |
| Below undergraduate | 3129 | 34.2 | 4161 | 60.9 | 7290 | 45.6 |
| Undergraduate | 5910 | 64.6 | 2663 | 39.0 | 8573 | 53.7 |
| Master's or above | 103 | 1.1 | 4 | 0.1 | 107 | 0.7 |
| Marital status | | | | | | |
| Married | 5678 | 62.1 | 4293 | 62.9 | 9971 | 62.4 |
| Unmarried | 3342 | 36.6 | 2415 | 35.4 | 5757 | 36.1 |

For peer review only

| | | | | | | |
|---|---|---|---|---|---|---|
| Divorced or Widowed | 122 | 1.3 | 120 | 1.7 | 242 | 1.5 |
| Professional title | | | | | | |
| Junior | 6249 | 68.4 | 4845 | 71.0 | 11094 | 69.5 |
| Intermediate | 2242 | 24.5 | 1587 | 23.2 | 3829 | 24.0 |
| Senior | 651 | 7.1 | 396 | 5.8 | 1047 | 6.5 |
| Employment form | | | | | | |
| Regular staff | 5077 | 55.5 | 3339 | 48.9 | 8416 | 52.7 |
| Temporary employee | 4065 | 44.5 | 3489 | 51.1 | 7554 | 47.3 |
| Average monthly income (RMB) | | | | | | |
| ≤ 3000 | 3747 | 41.0 | 4916 | 72.0 | 8663 | 54.3 |
| 3000–5000 | 4495 | 49.2 | 1867 | 27.3 | 6362 | 39.8 |
| 5000–10000 | 872 | 9.5 | 42 | 0.6 | 914 | 5.7 |
| > 10000 | 28 | 0.3 | 3 | 0.1 | 31 | 0.2 |
| Department | | | | | | |
| Emergency | 514 | 5.6 | 522 | 7.6 | 1036 | 6.5 |
| Internal medicine | 2850 | 31.2 | 1962 | 28.7 | 4812 | 30.1 |
| Surgery | 1903 | 20.8 | 1386 | 20.3 | 3289 | 20.6 |
| Gynaecology and Obstetrics | 426 | 4.7 | 610 | 8.9 | 1036 | 6.5 |
| Paediatrics | 516 | 5.6 | 627 | 9.2 | 1143 | 7.2 |
| Other | 2933 | 32.1 | 1721 | 25.3 | 4654 | 29.1 |
| Years of experience | | | | | | |
| 1–4 | 3511 | 38.4 | 3124 | 45.7 | 6635 | 41.5 |
| 5–10 | 2803 | 30.7 | 1597 | 23.4 | 4400 | 27.6 |
| 11–20 | 1534 | 16.8 | 1099 | 16.1 | 2633 | 16.5 |
| ≥ 21 | 1294 | 14.1 | 1008 | 14.8 | 2302 | 14.4 |
| Working time | | | | | | |
| 0–2 h | 164 | 1.8 | 52 | 0.8 | 216 | 1.3 |
| 2–4 h | 252 | 2.8 | 120 | 1.8 | 372 | 2.3 |
| 4–6 h | 264 | 2.9 | 113 | 1.6 | 377 | 2.4 |
| 6–8 h | 3713 | 40.6 | 3087 | 45.2 | 6800 | 42.6 |
| > 8 h | 4749 | 51.9 | 3456 | 50.6 | 8205 | 51.4 |
| Direct contact with patients | | | | | | |
| 0–2 h | 180 | 2.0 | 106 | 1.6 | 286 | 1.8 |
| 2–4 h | 237 | 2.6 | 251 | 3.7 | 488 | 3.0 |
| 4–6 h | 747 | 8.2 | 575 | 8.4 | 1322 | 8.3 |
| 6–8 h | 7978 | 87.2 | 5896 | 86.3 | 13874 | 86.9 |

**Incidence of exposure to WPV during the past 12 months**

In the 12-month survey period, 10,502 nurses experienced WPV. Total prevalence was 65.8%, of which verbal violence accounted for 64.9% (10369/15970), physical violence for 11.8% (1892/15970), and

For peer review only

sexual harassment for 3.9% (630/15970). The incidence rate of WPV in the different types of hospitals is shown in Table 2.

**Table 2** Incidence (%) of exposure to WPV

| Type | Tertiary hospitals | | | | | | County-level hospitals | | | | | |
|---|---|---|---|---|---|---|---|---|---|---|---|---|
| | Physical violence | | Verbal violence | | Sexual harassment | | Physical violence | | Verbal violence | | Sexual harassment | |
| | n | % | n | % | n | % | n | % | n | % | n | % |
| | 1047 | 11.5 | 5875 | 64.3 | 402 | 4.4 | 845 | 12.4 | 4494 | 65.8 | 228 | 3.3 |

**Prevalence of WPV for different demographic variables**

Nurses with frequent contact with patients were more likely to experience WPV in both tertiary and county-level hospitals (Table 3). There was a significant difference in the incidence rate of WPV among different professional titles for nurses in the tertiary hospitals ($\chi^2 = 6.6$, $P < 0.05$). In contrast, there was no significant difference in county-level hospitals ($\chi^2 = 1.5$, $P > 0.05$).

**Table 3** Characteristics and frequency distributions for WPV

| Characteristics | Tertiary hospitals (n=9142) | | | | County-level hospitals (n=6828) | | | |
|---|---|---|---|---|---|---|---|---|
| | n | % | $\chi^2$ | $P$ | n | % | $\chi^2$ | $P$ |
| Gender | | | | | | | | |
| Male | 143 | 61.1 | 1.8 | 0.181 | 85 | 59.4 | 3.2 | 0.072 |
| Female | 5820 | 65.3 | | | 4454 | 66.6 | | |
| Age (years) | | | | | | | | |
| ≤ 30 | 3283 | 64.0 | 15.4 | <0.001 | 2679 | 66.4 | 15.6 | <0.001 |
| 31–50 | 2548 | 67.3 | | | 1762 | 67.5 | | |
| ≥ 51 | 132 | 58.4 | | | 98 | 53.3 | | |
| Educational level | | | | | | | | |
| Below undergraduate | 1958 | 62.6 | 15.7 | <0.001 | 2707 | 65.1 | 9.7 | 0.008 |
| Undergraduate | 3941 | 66.7 | | | 1829 | 68.7 | | |
| Master's or above | 64 | 62.1 | | | 3 | 75.0 | | |
| Marital status | | | | | | | | |
| Married | 3813 | 67.2 | 27.8 | <0.001 | 2884 | 67.2 | 3.3 | 0.192 |
| Unmarried | 2065 | 61.8 | | | 1581 | 65.5 | | |
| Divorced or Widowed | 85 | 69.7 | | | 74 | 61.7 | | |
| Professional title | | | | | | | | |
| Junior | 4022 | 64.4 | 6.6 | 0.037 | 3217 | 66.4 | 1.5 | 0.482 |
| Intermediate | 1607 | 71.7 | | | 1068 | 67.3 | | |
| Senior | 434 | 66.7 | | | 254 | 64.1 | | |

For peer review only

| | | | | | | | | |
|---|---|---|---|---|---|---|---|---|
| Employment form | | | | | | | | |
| Regular staff | 3290 | 64.8 | 0.9 | 0.341 | 2213 | 66.3 | 0.1 | 0.733 |
| Temporary employee | 2673 | 65.8 | | | 2326 | 66.7 | | |
| Average monthly income (RMB) | | | | | | | | |
| ≤ 3000 | 2418 | 64.5 | 1.4 | 0.710 | 3275 | 66.6 | 0.3 | 0.955 |
| 3000–5000 | 2953 | 65.7 | | | 1233 | 66.0 | | |
| 5000–10000 | 574 | 65.8 | | | 29 | 69.0 | | |
| > 10000 | 18 | 64.3 | | | 2 | 66.7 | | |
| Department | | | | | | | | |
| Emergency | 418 | 81.3 | 101.2 | <0.001 | 429 | 82.2 | 158.9 | <0.001 |
| Internal medicine | 1889 | 66.3 | | | 1294 | 66.0 | | |
| Surgery | 1266 | 66.5 | | | 955 | 68.9 | | |
| Gynaecology & Obstetrics | 284 | 66.7 | | | 391 | 64.1 | | |
| Paediatrics | 351 | 68.0 | | | 483 | 77.0 | | |
| Other | 1755 | 59.8 | | | 987 | 57.4 | | |
| Years of experience | | | | | | | | |
| 1–4 | 2141 | 61.0 | 55.0 | <0.001 | 2006 | 64.2 | 35.2 | <0.001 |
| 5–10 | 1920 | 68.5 | | | 1148 | 71.9 | | |
| 11–20 | 1069 | 69.7 | | | 750 | 68.2 | | |
| ≥ 21 | 833 | 64.4 | | | 635 | 63.0 | | |
| Working time | | | | | | | | |
| 0–2 h | 87 | 53.0 | 26.1 | <0.001 | 32 | 61.5 | 67.9 | <0.001 |
| 2–4 h | 152 | 60.3 | | | 71 | 59.2 | | |
| 4–6 h | 165 | 62.5 | | | 53 | 46.9 | | |
| 6–8 h | 2367 | 63.7 | | | 1941 | 62.9 | | |
| > 8 h | 3192 | 67.2 | | | 2442 | 70.7 | | |
| Direct contact with patients | | | | | | | | |
| 0–2 h | 72 | 40.0 | 69.8 | <0.001 | 52 | 49.1 | 51.7 | <0.001 |
| 2–4 h | 127 | 53.6 | | | 144 | 57.4 | | |
| 4–6 h | 474 | 63.5 | | | 330 | 57.4 | | |
| 6–8 h | 5290 | 66.3 | | | 4013 | 68.1 | | |

**Characteristics of perpetrators and victims' responses**

Patients' relatives were the main perpetrators of WPV in both tertiary (83.1%) and county-level hospitals (85.2%). Most attacks on nurses occurred in the wards and during the day shift. More than 60% of the victims responded with tolerance, patience, and understanding (Table 4).

**Table 4** Characteristics of perpetrators and victims' responses

| | Tertiary hospitals (n=5963) | County-level hospitals (n=4539) |
|---|---|---|

For peer review only

| | n | % | n | % |
|---|---|---|---|---|
| Attack time | | | | |
| Day shift | 3558 | 59.7 | 2920 | 64.3 |
| Night shift | 1402 | 23.5 | 1195 | 26.4 |
| After work | 1003 | 16.8 | 424 | 9.3 |
| Attack site | | | | |
| Outpatient clinic | 388 | 6.5 | 351 | 7.7 |
| Ward | 2754 | 46.2 | 1933 | 42.6 |
| Doctor's office | 198 | 3.3 | 251 | 5.5 |
| Nurse's office or station | 1372 | 23.0 | 1291 | 28.5 |
| Treatment room | 127 | 2.1 | 162 | 3.6 |
| Other | 1124 | 18.9 | 551 | 12.1 |
| When the violent incident took place: | | | | |
| All alone | 1698 | 28.5 | 1319 | 29.1 |
| Other colleagues on the scene | 4265 | 71.5 | 3220 | 70.9 |
| Perpetrators * | | | | |
| Patients | 1784 | 35.9 | 1212 | 26.7 |
| Patients' relatives | 4131 | 83.1 | 3862 | 85.2 |
| Visitors | 748 | 15.0 | 573 | 12.6 |
| Other | 99 | 2.0 | 96 | 2.1 |
| Gender of the perpetrators* | | | | |
| Male | 3988 | 81.2 | 3380 | 83.1 |
| Female | 2461 | 50.1 | 1917 | 47.1 |
| Age group of the perpetrators (years)* | | | | |
| ≤ 20 | 258 | 5.1 | 258 | 5.7 |
| 21–30 | 1454 | 28.8 | 1624 | 35.9 |
| 31–40 | 2693 | 53.3 | 2263 | 50.0 |
| 41–50 | 2220 | 44.0 | 1529 | 33.8 |
| 51–60 | 917 | 18.2 | 1011 | 22.3 |
| ≥ 61 | 445 | 8.8 | 668 | 14.8 |
| Behavioral response to WPV * | | | | |
| Tolerance and avoidance | 3167 | 64.1 | 2601 | 63.3 |
| Patience and understanding | 2748 | 55.7 | 2415 | 58.8 |
| Give tit for tat | 80 | 1.6 | 36 | 0.9 |
| Try to explain before resorting to force | 438 | 8.9 | 310 | 7.5 |
| Ask colleagues for help | 940 | 19.0 | 537 | 13.1 |
| Turn to the managers or security staff for help | 1899 | 38.5 | 1334 | 32.5 |
| Ask for help from other patients and relatives | 287 | 5.8 | 165 | 4.0 |
| Call the police | 754 | 15.3 | 573 | 14.0 |
| Other | 166 | 3.4 | 72 | 1.8 |

* represents multiple choice

**Risk factors associated with WPV against nurses**

Participants' age, department, years of experience, and length of time in direct contact with patients were risk factors for WPV against nurses. Binary logistic regression analysis results demonstrated clearly that less than eight hours' working time is a protective factor against WPV for nurses in the county-level hospitals. Nurses in emergency departments were almost three times (OR = 2.993, 95%CI, 2.364 to 3.789) more likely to experience WPV in tertiary hospitals, and 3.387 times (95% CI, 2.648 to 4.332) more likely in county-level hospitals than nurses in other departments. Surprisingly, nurses with 5–10 years' of experience were at the highest risk of WPV in county-level hospitals, while those with 11–20 years' experience were at the highest risk in tertiary hospitals. Further, our results showed that the likelihood of WPV rises with the length of treatment time in county-level hospitals (Table 5).

## DISCUSSION

Previous studies have found that WPV exists in all hospitals but differs in some aspects.[27-31] The total incidence rate of WPV was 65.8% over the 12-month period. This is slightly higher than the figure of 64.48% reported for Guangzhou.[32] Compared with previous Chinese studies,[17-19] our study provides a comprehensive depiction of the incidence of WPV in Chinese comprehensive public hospitals. We have also documented the characteristics of the perpetrators and the coping style of nurses who experience WPV. This study demonstrates that WPV in China is higher than other countries.[33-36] Moreover, we found that WPV toward nurses is frequent, including verbal violence (64.9%), physical violence (11.8%), and sexual harassment (3.9%). This may be related to the fact that nurses are frequently in direct contact with patients and their relatives in their daily work. The findings also show that nurses in county-level hospitals are more likely to experience WPV than nurses in tertiary hospitals. This may be related to the level of education of patients and their relatives in county-level hospitals. In

For peer review only

**Table 5** Risk factors associated with WPV against nurses in hospitals, binary logistic results *

| Variable name | | Tertiary hospitals (n=9142) | | | County-level hospitals(n=6828) | | |
|---|---|---|---|---|---|---|---|
| | | Adjusted OR | 95%CI | *P*-value | Adjusted OR | 95%CI | *P*-value |
| Age group (years) | ≥ 51 | 1.0 | reference | 0.044 | 1.0 | reference | 0.027 |
| | ≤ 30 | 1.500 | (1.092–2.061) | 0.012 | 1.606 | (1.115–2.314) | 0.011 |
| | 31–50 | 1.387 | (1.036–1.855) | 0.028 | 1.551 | (1.120–2.146) | 0.008 |
| Department | Other | 1.0 | reference | <0.001 | 1.0 | reference | <0.001 |
| | Emergency department | 2.993 | (2.364–3.789) | <0.001 | 3.387 | (2.648–4.332) | <0.001 |
| | Internal medicine | 1.313 | (1.178–1.463) | <0.001 | 1.408 | (1.227–1.615) | <0.001 |
| | Surgery | 1.341 | (1.187–1.514) | <0.001 | 1.644 | (1.414–1.912) | <0.001 |
| | Gynaecology and Obstetrics | 1.322 | (1.065–1.641) | <0.001 | 1.268 | (1.044–1.539) | 0.016 |
| | Paediatrics department | 1.433 | (1.172–1.753) | <0.001 | 2.391 | (1.934–2.956) | <0.001 |
| Years of experience | 1–4 | 1.0 | reference | <0.001 | 1.0 | reference | <0.001 |
| | 5–10 | 1.426 | (1.268–1.604) | <0.001 | 1.479 | (1.277–1.712) | <0.001 |
| | 11-20 | 1.627 | (1.358–1.951) | <0.001 | 1.300 | (1.045–1.618) | 0.018 |
| | ≥ 21 | 1.368 | (1.131–1.656) | 0.001 | 1.192 | (0.949–1.498) | 0.131 |
| Working time (hours) | > 8h | | | | 1.0 | reference | <0.001 |
| | 0–2h | | | | 0.852 | (0.469–1.548) | 0.599 |
| | 2–4h | | | | 0.762 | (0.507–1.145) | 0.191 |
| | 4–6h | | | | 0.492 | (0.329–0.735) | 0.001 |
| | 6–8h | | | | 0.720 | (0.647–0.800) | <0.001 |
| Direct contact with patients (hours) | 0–2h | 1.0 | reference | <0.001 | 1.0 | reference | <0.001 |
| | 2–4h | 1.832 | (1.230–2.728) | 0.003 | 1.201 | (0.744–1.938) | 0.454 |
| | 4–6h | 2.722 | (1.939–3.819) | <0.001 | 1.220 | (0.785–1.895) | 0.377 |
| | 6–8h | 3.054 | (2.247–4.152) | <0.001 | 1.710 | (1.134–2.580) | 0.011 |

* This analysis used data from 44 public tertiary hospitals and 90 public county-level hospitals in China.

For peer review only

addition, the significant agricultural population in China is more likely to receive medical treatment at county-level hospitals, increasing the likelihood of WPV. In short, nurses have a high risk of experiencing WPV in the healthcare sector. [37-39]

Our survey also revealed that respondents aged 30 years or younger have a higher risk of WPV than their older colleagues. This phenomenon may be attributable to the fact that they have not been nurses for very long, and they lack work experience and communication skills. If that is the case, they need to practice their skills decrease the odds of making mistakes. Alternatively, because most of the nurses in this age group were single children, they are quick to anger when criticized by patients and their relatives, increasing the possibility of WPV. In contrast, nurses who are aged 51 years or older have a lower risk of WPV. This may be related to inherent respect for the elderly, a traditional virtue of the Chinese nation for thousands of years.[40]

As shown in our survey, the risk of WPV is not the same in different departments. Substantial previous studies have found that emergency departments are likely to have the highest incidence of WPV, followed by pediatric departments.[24 41] This increased risk may be because emergency departments deal with the most serious patients in complex situations, such as traffic accidents, food poisoning, and patients with alcoholism. Further, patients' relatives may be very worried and if nurses do not share information with patients and if the nurses' communication skills are inadequate, unnecessary conflicts may emerge. Pediatrics is another specialist department with a high risk of WPV. In China, all patients under the age of 14 are treated in pediatrics departments. The age range is different in countries around the world. The high risk of WPV in pediatric departments may be because patients are likely to be the parents' only child, and to have been overindulged. This may make nurses' work more difficult and increase the possibility of WPV. Therefore, hospitals and patients should make

For peer review only

joint efforts to reduce violent incidents. On the one hand, hospitals can establish a code green response team, comprising a charge nurse, security personnel, and primary nurse to manage the potentially violent situation. Dilman's study demonstrated that 85% of code green calls resulted in successful resolution of the violent incidents.[42] Preventive measures for WPV among nurses include the following: increase awareness of potentially violent patients; wear suitable clothes; maintain proper positioning when communicating with patients; keep a safe distance; maintain the correct posture; and listen actively. A study by Hill et al. showed a 65% reduction in staff injuries, from 2.2 per week to 0.77 per week, during the 1-year intervention period.[43] On the other hand, patients and their relatives should show respect to others and understand that nurses' work is complex and professional. With mutual understanding, nurses and patients can help to improve the nurse-patient relationship and achieve harmony. Stievano et al.'s study has shown that patient care can be affected when nurses are not respected.[44]

This study also demonstrated that years of experience and extended direct contact with patients are risk factors for WPV. Medical treatment processes, waiting time for patients, nurses' attitudes, and other aspects of hospital procedure need improvement to enhance patients' satisfaction and reduce conflict. Previous studies have shown that strengthening hospital management can improve patient satisfaction.[45]

Perpetrators' characteristics are equally noteworthy. In the present study, perpetrators are primarily patients' family members, followed by patients. Perpetrators are usually males aged 31 to 50, with an inverted "U" shape distribution. The ward is the main site of WPV in hospitals, accounting for 46.2% and 42.6% in tertiary and county-level hospitals, respectively. Ward management needs strengthening; for example, surveillance cameras and alarms should be installed in hospital ward corridors, lights

For peer review only

should be sufficiently bright in work areas during the night, and so on. Fine safety management can significantly improve the quality of nursing and patients' satisfaction, and reduce conflict.[46]

Although most patients and patients' relatives are well behaved, a few have underhand motives. Such people intentionally disrupt the normal medical order, and even pose a threat to the personal safety of medical staff and other patients, in order to seek monetary compensation. In China, a few years ago, the cost of "Yi Nao" (where in people employed by those in dispute with the hospital, together with family members of patients, took various measures to put pressure on the hospital, thereby profiteering) was very low. Occasionally, patients and their relatives may use illegal organizations to ask the hospitals for compensation directly, rather than use the normal legal procedure. Hospitals often make concessions by acquiescing to some unreasonable requirements to avoid trouble, preserve their reputation, and ensure normal medical order. This response has further contributed to the arrogance of those resorting to "Yi Nao." Fortunately, "Yi Nao" was officially criminalized in November 2015. The law now recognizes that "Yi Nao" risks the proper functioning of medical institutions and gives clear legal support to conviction and sentencing. Negative media coverage also has a huge impact on the nurse-patient relationship. Therefore, we suggest that the government should supervise the media to ensure the accuracy and authenticity of media coverage. We further advise hospitals to strengthen the training and management of nurses to reduce the physical and psychological damage to them from WPV. For instance, hospitals could provide violence-related training for nurses and provide post-WPV psychological support or a "debriefing room," instruct all staff about the value of nurses and foster nurses' pride in their work, develop an excellent hospital culture, etc. If nurses have the support of managers, they may be more willing to consult psychologists, psychiatrists, and mental health professionals to identify and treat any sudden disorder.[47]

For peer review only

We were surprised by victims' coping mechanisms, which were tolerance, avoidance, patience, and understanding in both tertiary and county-level hospitals. This response may reflect a culture of tolerance toward WPV in China. Most nurses turned to their managers or hospital security personnel for help when they experienced WPV. Thus, the organization's support and care may have significant potential for reducing the harm inflicted on nurses by WPV. Nurses should be familiar with all the resources in their work and family communities for solving any problems and should keep themselves in good mental health.[47]

The present study has several limitations. First, we collected data about whether nurses had experienced WPV over the previous 12 months, so there may be recall bias in the results. Second, we studied tertiary and county-level hospitals, but we did not study specialized hospitals. However, this study might be effective in preventing WPV in general and public hospitals.

In general, WPV can be prevented, according to the WHO's report on violence and health.[27] Therefore, to reduce violent incidents, we recommend that tertiary and county-level hospitals develop training against violence that is tailored to their particular situation, as well as the other measures we have suggested above. We also suggest improving psychological resilience for at–risk nurses to mitigate the negative impact of WPV and to prevent chronic diseases and reduce the incidence of mental illness.[48]

**CONCLUSIONS**

This study is based on a large sample survey of WPV in tertiary and county-level hospitals in China. The study has demonstrated that there is a high incidence rate of WPV and that occupational safety is insufficient. The frequent occurrence of WPV in emergency and pediatric departments is also

For peer review only

remarkable. The incidence of WPV of hospitals is an occupational health hazard and a serious threat to the wellbeing of nurses.

**Acknowledgements**

The authors thank nurses, managers, and the Chinese Hospital Association for their assistance and support for this project. We also acknowledge Xiaoli Jia and Min Ha for their assistance with the survey.

**Contributors**

LS and LF designed the study. DZ, CZ, TH, XP, YM and YH collected data. LS, LY, TS, LG and WL analysed the data. LS and LF drafted the manuscript. LS, LY, and LF revised the manuscript.

**Funding**

This study was funded by the National Natural Science Foundation of China, grant number 71473063.

**Competing interests** None declared.

**Data sharing statement** No additional data are available.

**REFERENCES**

1. Ramacciati N, Ceccagnoli A, Addey B. Violence against nurses in the triage area: An Italian qualitative study. *Int Emerg Nurs* 2015;23(4):274-280.

2. Alkorashy HA, Al Moalad FB. Workplace violence against nursing staff in a Saudi university hospital. *INT NURS REV* 2016;63(2):226–232.

3. Fallahi K M, Oskouie F, Ghazanfari N, *et al.* The Frequency, Contributing and Preventive Factors of Harassment towards Health Professionals in Iran. *IJCBNM* 2015;3(3):156-164.

For peer review only

4. Pai DD, Lautert L, Souza SBCD, *et al*. Violence, Burnout and Minor Psychiatric Disorders in Hospital Work. *Rev Esc Enferm USP* 2015;49(3):457-464.

5. Fafliora E, Bampalis V G, Zarlas G, *et al.* Workplace violence against nurses in three different Greek healthcare settings. *Work* 2015;53(3):551-560.

6. Ferri P, Silvestri M, Artoni C, *et al.* Workplace violence in different settings and among various health professionals in an Italian general hospital: a cross-sectional study. *Psychology Research & Behavior Management* 2016;9:263-275.

7. Fute M, Mengesha ZB, Wakgari N, *et al*. High prevalence of workplace violence among nurses working at public health facilities in Southern Ethiopia. *BMC Nurs* 2015;14:9.

8. Mihyun P, Sung-Hyun C, Hyun-Ja H. Prevalence and Perpetrators of Workplace Violence by Nursing Unit and the Relationship Between Violence and the Perceived Work Environment. *J Nurs Scholarsh* 2014;47(1):87-95.

9. Al-Omari H. Physical and verbal workplace violence against nurses in Jordan. *Int Nurs Rev* 2015;62(1):111–118.

10. Schablon A, Zeh A, Wendeler D, *et al.* Frequency and consequences of violence and aggression towards employees in the German healthcare and welfare system: a cross-sectional study. *BMJ Open* 2011;2(5):421-421.

11. Hogarth KM, Beattie J, Morphet J. Nurses' attitudes towards the reporting of violence in the emergency department. *Australas Emerg Nurs J* Published Online First: 23 May 2015. doi: 10.1016/j.aenj.2015.03.006.

12. Shen HC, Cheng, Y, Tsai PJ, *et al*. Occupational stress in nurses in psychiatric institutions in Taiwan. *J Occup Health* 2005;47:218–225.

For peer review only

13. Pompeii LA, Schoenfisch AL, Lipscomb HJ, *et al*. Physical assault, physical threat, and verbal abuse perpetrated against hospital workers by patients or visitors in six U.S. hospitals. *Am J Ind Med* 2015;58(11):194-204.

14. Hahn S, Muller M, Hantikainen V, *et al*. Risk factors associated with patient and visitor violence in general hospitals: results of a multiple regression analysis. *Int J Nurs Stud* 2013;50(3):374-85.

15. Albashtawy M, Aljezawi M. Emergency nurses' perspective of workplace violence in Jordanian hospitals: A national survey. *Int Emerg Nurs* 2015;24:61-65.

16. Jia XL, Zhou HZ, Zhao Y, *et al*. Investigation on hospital violence during 2003 to 2012 in China. *Chinese Hospitals* 2014;18(3):1-3.

17. Zhao Y, Wang FM, Zhou CY, *et al*. Investigation and analysis of present situation of workplace violence at tertiary hospitals and county hospitals in Heilongjiang Province. *Medicine and Society* 2016;29(9):35-37.

18. Yan JH, Hou J, Lin HQ, *et al*. Workplace violence in hospital setting to nursing staff and its influencing factors. *Journal of Nursing (China)* 2012;19(8A):4-7.

19. Chen CR, Duan XG. The causes of workplace violence on nursing and its corresponding countermeasures. *Chinese Journal of Nursing* 2010;16(5):572-574.

20. Liu L, Yang YL, Wang ZY, *et al*. Prevalence and Positive Correlates of Posttraumatic Stress Symptoms among Chinese Patients with Hematological Malignancies: A Cross-Sectional Study. *Plos One* 2015;10(12).

21. Wang X, Jing ZR, Zhao BL, *et al*. Survey on workplace violence and job burnout among nurses. *J Nurs Sci* 2015;(12):15-17.

22. Itzhaki M, Peles-Bortz A, Kostistky H, *et al.* Exposure of mental health nurses to violence

For peer review only

associated with job stress, life satisfaction, staff resilience, and post-traumatic growth. *International Journal of Mental Health Nursing* 2015;24(5):403–412.

23. Yang JJ, Gao LL. A survey on willingness of nurses' children engage in medical work. *Education Teaching Forum* 2015;(3):73-74.

24. Hahn SZA, Needham IEA. Patient and visitor violence in general hospitals: a systematic review of the literature. *Aggression Violent Behav* 2008;13:431–41.

25. Chen ZH, Wang SY. Psychiatric hospital and general hospital workplace violence study comparison. *Chinese Journal of Public Health* 2004;20:1316–1317.

26. ILO/ICN/WHO/PSI. Framework Guidelines for Addressing Workplace Violence in the Health Sector.Geneva: ILO/ICN/WHO/PSI Joint Programme on Workplace Violence in the Health Sector 2002:4-132.

27. Laschinger HK, Grau AL. The influence of personal dispositional factors and organizational resources on workplace violence, burnout, and health outcomes in new graduate nurses: A cross-sectional study. *Int J Nurs Stud* 2012;49:282–291.

28. Kitaneh M, Hamdan M. Workplace violence against physicians and nurses in Palestinian public hospitals: A cross-sectional study. *BMC Health Serv Res* 2012;12:469.

29. Branko G, Ivan E. Prevalence of violence towards nursing staff in Slovenian nursing homes. *Slovenian Journal of Public Health* 55(3):212-217.

30. Merecz D, Rymaszewska J, Moscicka A, *et al*. Violence at the workplace-a questionnaire survey of nurses. *Eur Psychiatry* 2006;21:442–450.

31. Ferns T. Under-reporting of violent incidents against nursing staff. *Nurs Stand* 2006;20(40): 41–45.

For peer review only

32. Chen ZH, Wang SY, Jing CX. Investigation on workplace violence phenomenon of two hospitals in Guangzhou. *Chinese Journal of Preventive Medicine* 2003;37:358–360.

33. Zampieron A, Galeazzo M, Turra S, *et al.* Perceived aggression towards nurses: study in two Italian health institutions. *J Clin Nurs* 2010;19:2329–41.

34. Tiruneh BT, Bifftu BB, Tumebo A A, *et al.* Prevalence of workplace violence in Northwest Ethiopia: a multivariate analysis. *BMC Nursing*, 2016;15(1):1-6.

35. Franz S, Zeh A, Schablon A, *et al*. Aggression and violence against health care workers in Germany–a cross sectional retrospective survey. *BMC Health Serv Res* 2010;10:51.

36. Hahn S, Muller M, Needham I, *et al.* Factors associated with patient and visitor violence experienced by nurses in general hospitals in Switzerland: a cross-sectional survey. *J Clin Nurs* 2010; 19:3535–46.

37. Magin PJ, May J, McElduff P. Occupational violence in general practice: a whole-of-practice problem. Results of a cross-sectional study. *Australian Health Review* 2011;35(1):75–80.

38. Kwok RP, Law YK, Li KE, *et al*. Prevalence of workplace violence against nurses in Hong Kong. *Hong Kong Med J* 2006;12:6–9.

39. Tiruneh BT, Bifftu BB, Tumebo AA, *et al.* Prevalence of workplace violence in Northwest Ethiopia: a multivariate analysis. *BMC Nursing* 2016;15(1):1-6.

40. Sun T, Wang S, Wang N, *et al*. Epidemiological characteristics and variance analysis of workplace violence in public hospitals. *Practical Preventive Medicine* 2014;21(4):478-480.

41. Shaw J. Staff perceptions of workplace violence in a pediatric emergency department. *Work* 2015;51(1):39-49.

42. Dilman Y. Code green for workplace violence. *Critical Care Nurse* 2015;35:34-35.

For peer review only

43. Hill AK, Lind MA, Tucker D, *et al*. Measurable results: Reducing staff injuries on a specialty psychiatric unit for patients with developmental disabilities. *Work* (*Reading, Mass.*) 2015;51(1): 99-111.

44. Stievano A, Bellass S, Rocco G, *et al*. Nursing's professional respect as experienced by hospital and community nurses. *Nursing Ethics* 2016.

45. Al Fraihi KJ, Latif SA. Evaluation of Outpatient Service Quality in Eastern Saudi Arabia. Patient's expectations and perceptions. *Saudi Medical Journal* 2016;37(4):420-428.

46. Mei XM. Application of Fine Management in Safety Management in Geriatric Ward. *Hospital Management Forum* 2016.

47. Teris C, Yip PSF. Depression, anxiety and symptoms of stress among Hong Kong nurses: a cross-sectional study. *Inter J Env Res Pub Heal* 2015;12(9):11072-11100.

48. Cheung T, Yip PS. Lifestyle and Depression among Hong Kong Nurses. *Inter J Env Res Pub Heal*, 2016;13(1):135.

For peer review only

Checklist of ***cross-sectional studies***

| | Page number, line number |
|---|---|
| **Title** | 1, 4-6 |
| **Abstract** | 2, 4-58 |
| **Strengths and limitations of this study** | 3, 4-27 |
| **Introduction** | |
| Background | 4, 4-57; 5, 4-14 |
| Objectives | 5, 16-24 |
| **Methods** | |
| Study design | 5, 37-52 |
| Setting | 5, 52-57; 6, 4-19 |
| Participants | 6, 21-27 |
| Questionnaire | 6, 41-57; 7, 4-29 |
| Data measurement | 7, 36-57; 8, 4 |
| Study size | 6, 28-32 |
| Quantitative variables | 7, 36-42 |
| Ethical considerations | 8, 11-19 |
| **Results** | |
| Participants | 8, 26-34 |
| Descriptive data | 8, 36-58; 9, 3-48 |
| Outcome data | 9, 52-57 |
| Main results | 10; 11; 12;14 |
| Other analyses | 10, 8-15; 12 |
| **Discussion** | |
| Key results | 13,32-57; 15, 4-56 |
| Interpretation | 16, 3-32; 17, 9-57 |
| Discuss the external validity of the study results | 17, 9-27 |
| **Conclusions** | 18, 51-57; 19, 4-7 |
| **Other information** | |
| Acknowledgements | 18, 14-19 |
| Contributors | 18, 24-27 |
| Funding | 18, 34-36 |
| Competing interests | 18, 34 |
| Ethical approval | 18, 47-52 |
| **References** | 19; 20; 21; 22; 23; 24 |

For peer review only
